# Disparities in pediatric cancer survivorship care: A systematic review

**DOI:** 10.1002/cam4.6426

**Published:** 2023-08-08

**Authors:** Erin M. Mobley, Diana J. Moke, Joel Milam, Carol Y. Ochoa‐Dominguez, Julia Stal, Halle Mitchell, Naghmeh Aminzadeh, Maria Bolshakova, Raymond B. Mailhot Vega, Jennifer Dinalo, Aneesa Motala, Susanne Hempel

**Affiliations:** ^1^ Department of Surgery, College of Medicine University of Florida Jacksonville Florida USA; ^2^ Department of Hematology, Oncology, and Blood and Marrow Transplantation Children's Hospital Los Angeles Los Angeles California USA; ^3^ Department of Pediatrics, Keck School of Medicine University of Southern California Los Angeles California USA; ^4^ Southern California Center for Young Adult Cancer Survivorship Research Los Angeles and Irvine California USA; ^5^ Department of Epidemiology and Biostatistics, Program in Public Health University of California Irvine Irvine California USA; ^6^ Department of Radiation Medicine and Applied Sciences, School of Medicine University of California San Diego San Diego California USA; ^7^ Department of Preventive Medicine, Keck School of Medicine University of Southern California Los Angeles California USA; ^8^ Southern California Evidence Review Center, Keck School of Medicine University of Southern California Los Angeles California USA; ^9^ Department of Radiation Oncology, College of Medicine University of Florida Jacksonville Florida USA

**Keywords:** cancer survivors, health equity, health services accessibility, healthcare disparities

## Abstract

**Background:**

Childhood cancer survivors (CCS) experience many long‐term health problems that can be mitigated with recommended survivorship care. However, many CCS do not have access to survivorship care nor receive recommended survivorship care. We reviewed the empirical evidence of disparities in survivorship care for CCS.

**Methods:**

This systematic review searched PubMed, CINAHL, and PsycINFO for studies on survivorship care for CCS (PROSPERO: CRD42021227965) and abstracted the reported presence or absence of disparities in care. We screened 7945 citations, and of those, we reviewed 2760 publications at full text.

**Results:**

A total of 22 studies reported in 61 publications met inclusion criteria. Potential disparities by cancer treatment (*N* = 14), diagnosis (*N* = 13), sex (*N* = 13), and current age (*N* = 13) were frequently studied. There was high quality of evidence (QOE) of survivorship care disparities associated with non‐White race, Hispanic ethnicity, and being uninsured. Moderate QOE demonstrated disparities among CCS who were unemployed and older. Lower QOE was found for disparities based on cancer diagnosis, cancer treatment, age at diagnosis, time since diagnosis, sex, insurance type, income, educational attainment, and geographic area.

**Conclusions:**

We found strong empirical evidence of disparities in survivorship care for CCS associated with race, ethnicity, and insurance status. Multiple other disparate groups, such as those by employment, income, insurance type, education, cancer diagnosis, age at diagnosis, time since diagnosis, cancer treatment, geographic area, sex, and self‐identified gender warrant further investigation. Prospective, multilevel research is needed to examine the role of other patient characteristics as potential disparities hindering adequate survivorship care in CCS.

## Graphical abstract

Across identified empirical research, high quality of evidence indicated that childhood cancer survivors of non‐White race or Hispanic ethnicity and who lack of insurance were associated with disparities in survivorship care engagement. These findings underscore the importance of filling the identified gaps in the literature with future prospective studies to improve our understanding of disparities faced by childhood cancer survivors, particularly using a multilevel approach.

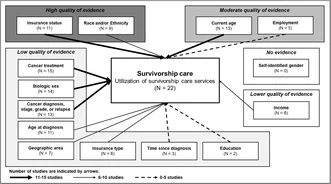

## 1 INTRODUCTION

In the United States (U.S.), there are more than half a million survivors of childhood cancer diagnosed before 21 years of age.^1^ The 5‐year overall survival rate for childhood cancer has improved significantly from the 1970s, when it was about 58%–85% now.^2^ This increase in survival is attributed to improved treatment options for patients with risk‐stratification, including single and multimodality treatment with surgery, chemotherapy, radiation, immunotherapy, and/or stem cell transplantation, as well as improvements in supportive care. Childhood cancer survivors (CCS) face many challenges regarding long‐term health outcomes due to their cancer diagnosis and treatment (known as “late effects”). Late effects may range in aquity and complexity, and frequently result in multimorbidity.^3^ Late effects for CCS may manifest as cardiovascular disease, heart failure, decreased pulmonary function, fertility challenges, hormonal changes, kidney failure, osteopenia, osteoporosis, neurocognitive deficits, secondary malignancies, and other comorbidities.^3^ As a result of these late effects, CCS are at risk for adverse physical, psychosocial, functional, and behavioral outcomes. Furthermore, CCS experience inequities in their social, economic, and health‐related quality of life compared to their non‐cancer surviving peers.^4^, ^5^ These nonclinical inequities can lead to disparities independently from biological factors.^6^

Put simply, health disparities are not just differences in health—they are differences that are inequitable, unjust, or unacceptable, according to the National Academies.^7^ The National Institute of Minority Health and Health Disparities defines populations that experience disparities as people belonging to racial or ethnic minority groups, people with lower socioeconomic status, those from underserved areas, or those who identify as sexual or gender minorities.^8^ Broadly, the National Institutes of Health and National Cancer Institute use the term disparities in legislation, operational and research definitions, and in the strategic plan.^7^, ^9^, ^10^, ^11^

Survivorship care is a clinical approach to support survivors using risk‐based methods (e.g., due to treatment exposure to potentially harmful therapies) of surveillance, screening, management, and prevention of late effects, in conjunction with coordination of care with both specialty and primary care providers to improve the health and well‐being of cancer survivors.^1^ The Children's Oncology Group (COG) recommends risk‐based long‐term follow‐up care for survivors of pediatric malignancies to mitigate late effects, increase quality of life, and decrease healthcare costs.^12^ However, the majority of CCS do not receive recommended survivorship care, particularly following the transition into adulthood, resulting in disparities in survivorship care engagement among this population.^1^ Despite the increasing awareness regarding the importance of survivorship care, we lack understanding of subgroups of CCS who experience disparities in survivorship care engagement.

This systematic review fills the gaps described above and builds on a larger project commissioned by the National Cancer Institute through the Agency for Healthcare Research and Quality's Evidence‐based Practice Center program to inform the research agenda following the implementation of the U.S. Childhood Cancer Survivorship, Treatment, Access, and Research (STAR) Act.^11^ The objective of this systematic review was to examine the empirical evidence of disparities in the survivorship care of CCS. The review was guided by the key question: *What are the disparities in survivorship care for childhood cancer survivors?*

## 2 METHODS

We registered the systematic review in PROSPERO (CRD42021227965) and followed a detailed review protocol.^13^ The work was supported by nine subject matter experts with expertise in patient, family, and caregiver perspective; clinical implications, patient care, and disparities research; health services research and access to care for populations that experience disparities; and administrative and payer perspectives. The key informants reviewed the key question, our definition of disparities, and possible sources of care disparities in CCS. This work is part of a larger project on disparities and barriers to care in CCS and interventions to support CCS.^14^

### 2.1 Sources and searches

A librarian specializing in systematic reviews planned, completed, and documented the search strategy shown in Appendix S1. We searched the research databases PubMed, CINAHL, and PsycINFO for published research on survivorship care. We identified studies through two search strategies: (1) we identified publications that focused on disparities directly (e.g., addressed disparities in the title, abstract, or key word), and (2) we searched for empirical research studies on CCS patients (i.e., which did not address disparities in the title, abstract, or key word). We reference‐mined identified literature, and subject matter experts provided additional input to confirm that relevant studies were included.

### 2.2 Screening and abstraction

Table A‐1 in the Appendix S1 displays the framework of PICOTSS (population, intervention, or independent variables, comparison groups, outcomes, timing, study setting, and study design, or other limiters). Study populations eligible for inclusion were required to have at least 50% CCS. We defined CCS as those diagnosed before age 21, who had received primary acute treatment for any cancer, were currently in remission, and currently receiving or eligible to receive survivorship care services, care plans, and/or models of follow‐up care at time of study. We conducted both the literature screening and data abstraction steps in a database designed for systematic reviews using a web‐based interface. Literature reviewers first screened all identified citations, and those determined to be potentially relevant by at least one reviewer were obtained and screened at the full‐text level. Citations that were excluded were later screened with a machine learning algorithm to avoid missing any potentially relevant citations. Two reviewers screened full text publications independently with the prespecified eligibility criteria (i.e., PICOTSS), and we resolved any inconsistent decisions by group discussion and consensus at a team meeting.

We used the definition of populations or groups that experience health disparities endorsed by the National Institute of Minority Health and Health Disparities and the National Academies: racial/ethnic minorities (including those who are Black/African American, Hispanic/Latino, American Indian/Alaska Native, Asian American, Native Hawaiian, and other Pacific Islander); socioeconomic status; underserved urban or rural areas; sexual and gender minorities; and educational attainment.^7^, ^8^ We also used disparity categories suggested by key informants as additional potential sources of disparities, such as employment, age at study, age at diagnosis, time since diagnosis, insurance status, cancer diagnosis (e.g., type, grade, stage, relapse), and cancer treatment (e.g., type, intensity). We included all studies that evaluated potential disparities regardless of the result of the evaluation, meaning that we included all studies reporting on the presence or the absence of disparities in care in studies that addressed patient characteristics potentially associated with disparities.

For abstraction of data from included studies, one reviewer abstracted applicable data and an experienced content expert checked the data for accuracy. We considered all available publications relevant to a study (the included participants define a study) and consolidated information in the data abstraction form. This avoided counting the same study multiple times and instead provided a complete record of the results of the overarching, most pertinent data regarding disparities. In addition to abstracting results on disparities, we also recorded the study design, country of origin, study participant characteristics (i.e., cancer type, proportion of CCS compared to adult survivors), and type of analysis and outcome(s) assessed. We abstracted the results of assessments of potential disparities and documented reported effect size estimates, the direction of effects, and the statistical significance of results, documenting the presence or the absence of disparities reported in the study.

### 2.3 Critical appraisal and synthesis

To assess risk of bias in individual studies, we used the Quality In Prognostic Studies (QUIPS) tool for prognostic factor research addressing six domains: study participation, study attrition, prognostic factor measurement, outcome measurement, study confounding, and statistical analysis and reporting.^15^ Comprehensive evidence and risk of bias tables document the presence and absence of disparities for each content category and report the risk of bias for the included studies.

We documented findings across studies in a Summary of Findings table. We assessed the quality of evidence (QOE) across studies using the Grading of Recommendations Assessment, Development and Evaluation (GRADE) framework for prognostic research.^16^ In brief, although based exclusively on observational research designs, we did not use *low* QOE as the starting point to avoid floor effects and instead started at *high* and downgraded where findings were exclusively based on retrospective rather than prospective studies for study limitations. In addition, we downgraded for inconsistency of results across studies, indirectness of outcome measures, imprecision of estimates, and potential reporting bias. The quality could be upgraded for large effects, exposure‐response gradient, and where plausible confounding would mask an association. We differentiated *high*, *moderate*, *low*, and *very low* evidence corresponding to the level of uncertainty associated with the summary evidence statement.

## 3 RESULTS

**FIGURE 1:**
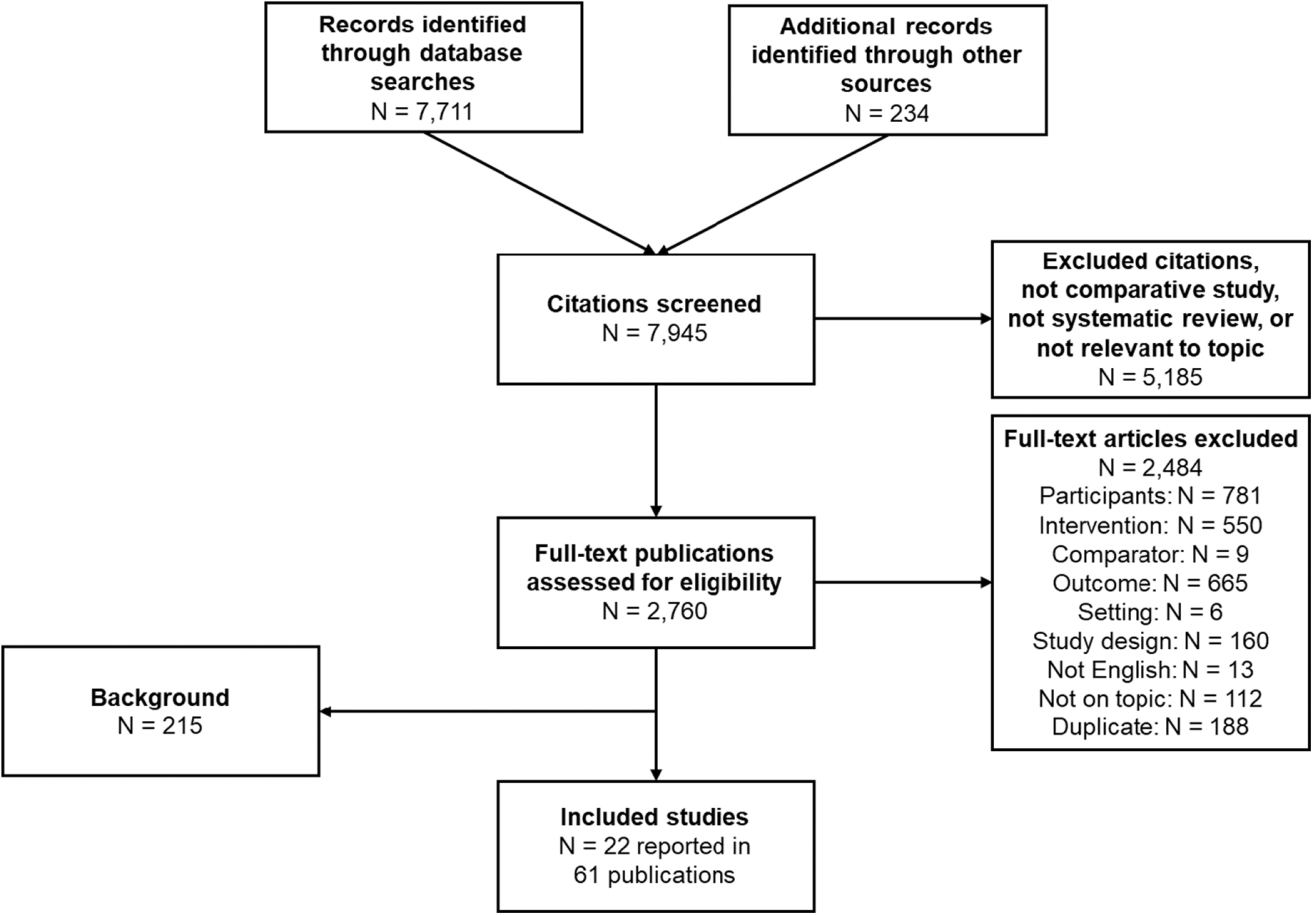
PRISMA flow diagram.

We screened 7945 citations for relevance, we screened 2760 publications as full text against the prespecified eligibility criteria, and we identified 22 studies (as reported in 61 individual publications) examining disparities in survivorship care for CCS.^17^, ^18^, ^19^, ^20^, ^21^, ^22^, ^23^, ^24^, ^25^, ^26^, ^27^, ^28^, ^29^, ^30^, ^31^, ^32^, ^33^, ^34^, ^35^, ^36^, ^37^, ^38^, ^39^, ^40^, ^41^, ^42^, ^43^, ^44^, ^45^, ^46^, ^47^, ^48^, ^49^, ^50^, ^51^, ^52^, ^53^, ^54^, ^55^, ^56^, ^57^, ^58^, ^59^, ^60^, ^61^, ^62^, ^63^, ^64^, ^65^, ^66^, ^67^, ^68^, ^69^ Figure 1 presents the PRISMA flow diagram that visually depicts the number of studies that were both included and excluded at each stage of the review process and the reasons for exclusion.

### 3.1 Study characteristics

**FIGURE 2:**
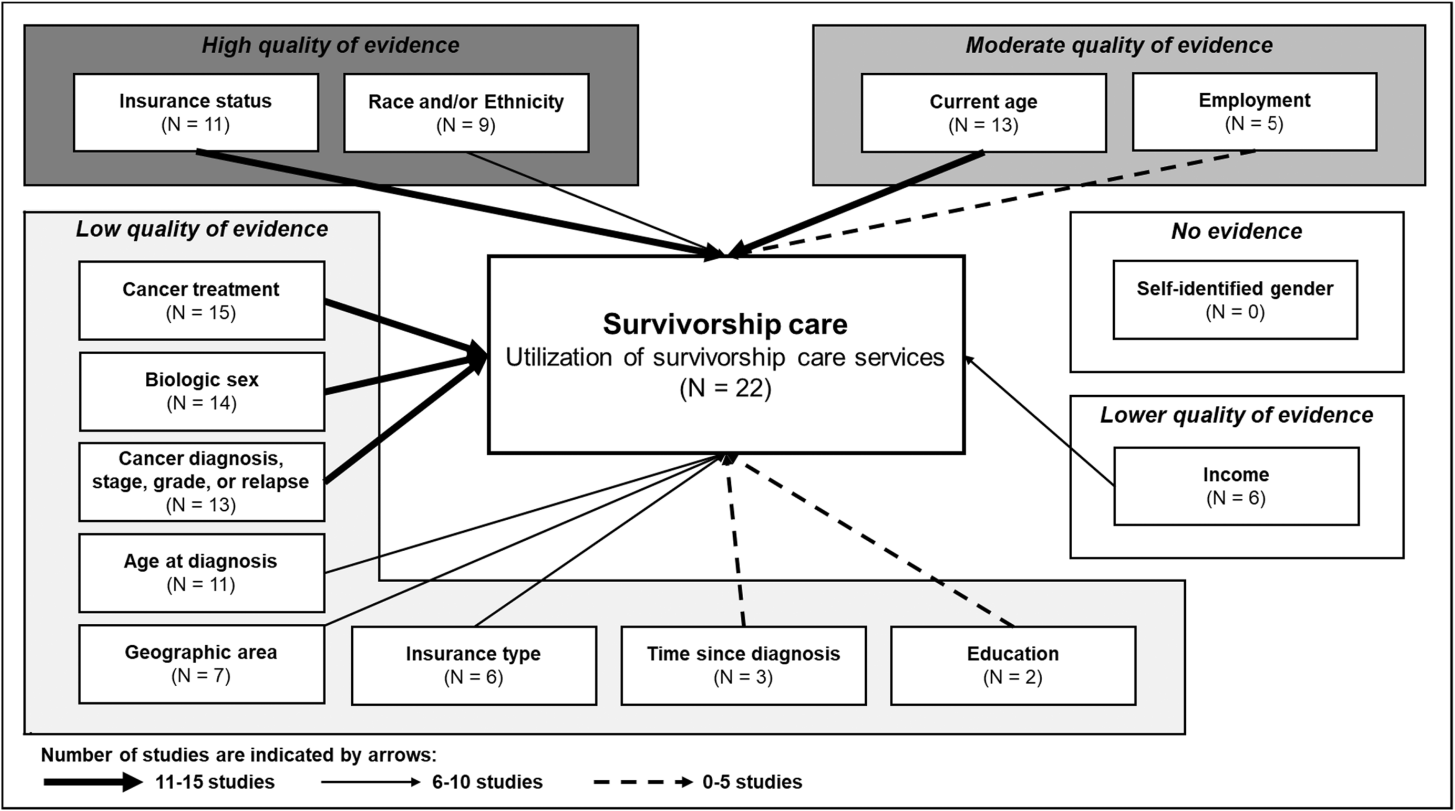
Variables mapped to survivorship care disparities by quality of evidence and number of studies.

**FIGURE 3:**
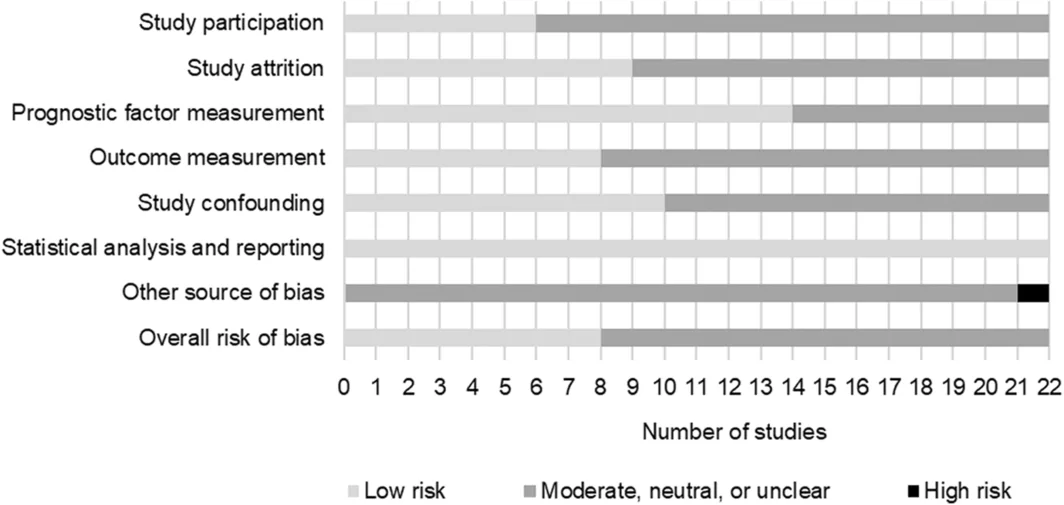
Risk of bias.

The identified studies described CCS in the United States, United Kingdom, Canada, Switzerland, and Netherlands. The most frequently studied areas of potential disparities (see Table 1) were cancer treatment (*N* = 14), cancer diagnosis (*N* = 13), biological sex (*N* = 13), current age (*N* = 13), insurance status (*N* = 11), age at diagnosis (*N* = 10), and race or ethnicity (*N* = 8). Less frequently studied potential disparity sources were geographic area (*N* = 7), income (*N* = 6), insurance type (*N* = 6), employment (*N* = 5), and education (*N* = 2). Figure 2 depicts assessed disparity categories mapped to survivorship care disparities in the included studies. Figure 3 displays the risk of bias in the included studies. In the Appendix S1, Table A‐2 provides the evidence table for included studies and Table A‐3 displays the risk of bias assessment. All studies had 100% CCS in their evaluated sample.

**TABLE 1:** Summary of findings.

Variable	Number of studies by type: citation	Disparities assessment findings: significant	Disparities assessment findings: not significant	Summary across studies	Quality of evidence Reason for downgrading
Insurance status	1 Prospective study: Crom, 2007^17^ 10 Retrospective observational studies: Berg, 2016^21^ Berkman, 2019^22^ Daly, 2019^23^ Gardner, 2014^24^ May, 2017^26^ Milam, 2015^29^ Oeffinger, 2004^31^ Szalda, 2016^34^ Welch, 2017^36^ Zheng, 2016^38^	Favors those who are insured: Those who were currently insured experienced a decrease in the odds of difficulty obtaining care (OR 0.18; CI 0.12, 0.26) compared to those who were uninsured, after adjusting for sex, age at survey, marriage, employment status, age at diagnosis, cancer diagnosis, and radiation therapy.^17^ In multivariable analysis, lack of health insurance was associated with less than annual healthcare provider visits (OR 0.04; CI 0.01, 0.33), after adjusting for current age, sex, and chemotherapy exposure.^21^ In a multivariable analysis, participants with self‐pay status or uninsured were significantly more likely to have missed visits (+8.46%, *p* = 0.005) when adjusting for tumor grade and race.^22^ In multivariable analyses and in comparison with those with insurance, those who were uninsured (OR 3.4; CI 1.2, 9.2) were more likely to be lost to follow‐up for more than 1000 days and not attend survivorship clinic, when adjusting for diagnosis, treatment exposure, SCT exposure, age group at diagnosis, and race (global overall *p*‐value = 0.02).^26^ Survivors who had any health insurance were more likely (OR 3.40; CI 1.10, 8.41) to access survivorship care in comparison with those who were uninsured, after adjusting for sex, race/ethnicity, treatment intensity, family influence on healthcare decisions, having a regular doctor, health care self‐efficacy, and post‐traumatic growth.^29^ Those who were uninsured (in comparison with those who were privately covered) were less likely to have survivorship care (OR 0.28; CI 0.16, 0.49), after adjusting for socioeconomic status at diagnosis, socioeconomic status at survey, age at diagnosis, education, treatment intensity, sex, race/ethnicity, and age at survey. In comparison with those who were privately insured at diagnosis, survivors who were uninsured were less likely to access survivorship care (OR 0.26; CI 0.14, 0.48), after adjusting for average socioeconomic status from diagnosis to survey, age at diagnosis, treatment intensity, sex, race/ethnicity, and age at survey.^29^, ^50^ Survivors who were insured were more likely to have had a recent survivorship visit (OR 4.20; CI 1.65, 10.67), after adjusting for current age, time since diagnosis, sex, treatment intensity, Spanish‐speaking Hispanic parent, English‐speaking Hispanic parent, and English‐speaking non‐Hispanic parent.^29^, ^52^ In the multivariable model and in comparison with survivors who were not insured, those who were insured (OR 2.06; CI 1.28, 3.32) were more likely to access survivorship care, after adjusting for years since diagnosis, current age, sex, race and ethnicity, socioeconomic status, high levels of depressive symptoms, number of late effects, treatment intensity, received a written treatment summary, reported having a doctor for regular (non‐cancer) care, discussed cancer‐related follow‐up care with a doctor in the last 2 years, knowledge of the need for life‐long survivorship care, health care self‐efficacy, and family influence of health care decisions.^29^, ^46^ In comparison with survivors who were insured, those who were uninsured experienced an increased odds (OR 1.7; CI 1.3, 2.2) of having general care (rather than risk‐based survivorship care), after adjusting for sex, race/ethnicity, current age, age at interview, annual household income, educational attainment, employment status, poor emotional health, cancer‐related anxiety, cancer‐related pain, poor physical health, and grade of chronic disease.^31^, ^58^ In univariate analysis, having insurance was the only significant characteristic associated with engagement in adult‐oriented follow‐up care (*p* < 0.005); no survivors who were uninisured had follow‐up care.^34^, ^69^ At 5 years from diagnosis, patients were more likely to participate in follow‐up if they had insurance at diagnosis (OR 3.4; CI 1.2, 9.9), controlling for institutional clustering. At 10 years after diagnosis, insurance status at diagnosis remained a strong predictor (OR 3.7; CI 1.0, 9.1) for follow‐up care, controlling for institutional clustering. At 5‐ and 10‐year post‐diagnosis, being uninsured was negatively associated with follow‐up care (5‐year follow‐up: OR 0.24; CI 0.026, 0.46; 10‐year follow‐up: OR 0.28; CI 0.11, 0.71), when compared to those with private or military insurance coverage.^36^ After adjusting for sex and age at diagnosis, survivors covered by private insurance (HR 2.90; CI 1.75, 4.81) or public insurance (HR 2.05; CI 1.14, 3.71) were more likely to attend survivorship clinic in comparison with those without insurance.^38^ Does not favor those who were insured: In univariable analysis, use of a mental health professional was less likely to be associated with private insurance coverage, in comparison with those with public insurance or uninsured (*p* = 0.040).^24^ Does not favor those who experienced a change in their insurance coverage: In the multivariable model, survivors who experienced a change in insurance coverage from diagnosis to survivorship resulting in a gain in coverage (*b*: −0.05, SE: 0.02) or a loss in coverage (*b*: −0.15; SE: 0.04) had a decrease in the predicted probability of having a survivorship care visit in the prior 2 years; however, those covered by Medicaid and/or Medicare at diagnosis had an increase in the probability of having a survivorship care visit in the prior 2 years (*b*: 0.05; SE: 0.02); all models were adjusted for current age, sex, race and ethnicity, marital status, educational attainment, receipt of supplemental income, insurance at diagnosis, insurance understanding, socioeconomic status, treatment intensity, children, employment status, difficulty with a referral to a specialist, ability to see a doctor when needed, friend/family influence of health decisions, cancer diagnosis, and self‐rated health.^29^, ^46^	There was not a significant relationship for who were uninsured in predicting an initial survivorship visit, after adjusting for sex, race/ethnicity, therapeutic modalities, current age, and distance from the clinic, in comparison with those who were privately insured.^23^	Being uninsured is associated with a disparity in survivorship care.	High Reason for downgrading: not applicable
Race and ethnicity	1 Prospective study: Crom, 2007^17^ 8 Retrospective observational studies: Baedke, 2022^18^ Barakat, 2012^19^ Berkman, 2019^22^ Daly, 2019^23^ May, 2017^26^ Milam, 2015^29^ Oeffinger, 2004^31^ Welch, 2017^36^ Zheng, 2016^38^	Favors those of non‐Hispanic White race and ethnicity: Publicly insured non‐Hispanic White survivors were more likely to report a cancer‐related visit (OR 1.20; CI 1.08, 1.34) and a cancer center visit (OR 1.31; CI 1.06, 1.62) compared to privately insured non‐Hispanic White survivors. Publicly insured Hispanic survivors were more likely to report a cancer‐related visit (OR 1.41; CI 1.12, 1.78) and a cancer center visit (OR 1.88; CI 1.28, 2.77) compared to privately insured Hispanic survivors. All models adjusted for age, sex, household income, highest level of educational attainment, and Grade 3 or 4 chronic condition.^31^, ^61^ Does not favor those of non‐Hispanic White race and ethnicity: Uninsured non‐Hispanic White survivors were less likely to report survivor focused‐care for a cancer‐related visit (OR 0.82; CI 0.74, 0.90) and a cancer center visit (OR 0.79; CI 0.66, 0.94) compared to privately insured non‐Hispanic White survivors.^31^, ^61^ Does not favor those of Hispanic ethnicity: In multivariable models and in comparison with non‐Hispanic White survivors, those who were Hispanic (OR 0.69, 0.51–0.95) were less likely to access survivorship care, after adjusting for years since diagnosis, current age, sex, socioeconomic status, health insurance coverage, high levels of depressive symptoms, number of late effects, treatment intensity, received a written treatment summary, reported having a doctor for regular (non‐cancer) care, discussed cancer‐related follow‐up care with a doctor in the last 2 years, knowledge of the need for life‐long survivorship care, health care self‐efficacy, and family influence of health care decisions.^29^, ^46^ Does not favor those of non‐White race: Non‐Whites were nearly two times more likely to be non‐attenders at survivorship clinic (OR 1.88; CI 1.19, 2.97) compared to Whites, after adjusting for age, socioeconomic status, race, years from diagnosis, additional cancer event, insurance status, means of travel, distance from hospital, scheduled social work consultation, and type of clinic visit.^17^, ^39^ In univariable analysis, patients of non‐White race were more likely to have no shows for survivorship visits (OR 0.27, *p* = 0.001).^19^ In the multivariable model, Black race was a significant predictor of missed visits (+6.90%, *p* < 0.001) when adjusting for tumor grade and insurance status.^22^ As compared to White survivors, those of Black race (HR 0.64, 0.52–0.79) or other races (HR 0.70; CI 0.49, 0.99) were less likely to have an initial survivorhip visit, when adjusting for sex, therapeutic modalities, current age, insurance status, and distance from the clinic.^23^ In multivariable analyses and in comparison with White participants, individuals who were Black (OR 2.0; CI 0.9, 4.4) or of other races (OR 2.0; CI 0.4, 9.2) were more likely to be lost to follow‐up for more than 1000 days and not attend survivorship clinic, where Hispanic individuals (OR 0.7; CI 0.4, 1.1) were less likely to be lost to follow‐up for more than 1000 days and not attend survivorship clinic, when adjusting for diagnosis, treatment exposure, stem cell transplant exposure, age group at diagnosis, and insurance status (global overall *p* = 0.03).^26^ In a multivariable model and in comparison with non‐Hispanic White survivors, those who were other races or ethnicities (OR 0.69, 0.48–0.99) were less likely to access survivorship care, after adjusting for years since diagnosis, current age, sex, socioeconomic status, health insurance coverage, high levels of depressive symptoms, number of late effects, treatment intensity, received a written treatment summary, reported having a doctor for regular (non‐cancer) care, discussed cancer‐related follow‐up care with a doctor in the last 2 years, knowledge of the need for life‐long survivorship care, health care self‐efficacy, and family influence of health care decisions.^29^, ^46^ In comparison with non‐Hispanic White survivors, minority survivors were less likely (OR 0.79; CI 0.64, 0.96) to report absence of a cancer center medical visit, after adjusting for age at study, sex, educational attainment, health insurance, health status, concern for future heatlh, and high‐risk treatment.^31^ In comparison with White non‐Hispanic CCS, survivors who were Black (OR 2.1; CI 1.3, 3.3) were more likely to report general care rather than risk‐based survivorship care and those who were of other races (OR 0.8; CI 0.7, 1.0) were less likely to report general care versus risk‐based survivorship care, after adjusting for sex, current age, age at interview, annual household income, educational attainment, employment status, insurance coverage, poor emotional health, cancer‐related anxiety, cancer‐related pain, poor physical health, and grade of chronic disease.^31^, ^58^ Survivors who were from the other race/ethnicity groups who reported some form of medical care at baseline (either survivor‐focused or general) experienced an increased risk of reporting no care at follow‐up (OR 2.1; CI 1.2, 3.7) compared to non‐Hispanic White CCS, after adjusting for sex, annual household income, chronic disease status, and education.^31^, ^59^ In multivariable analysis and in comparison with privately insured Whites, privately insured Blacks (OR 1.43; CI 1.01, 2.01), uninsured Blacks (OR 4.41; CI 2.93, 6.64) and uninsured Whites (OR 3.53; CI 2.75, 4.55) were more likely to forgo needed care, and publicly insured Whites (OR 0.69; CI 0.53, 0.89) were less likely to forgo needed care, after adjusting for current age, sex, treatment era, cancer diagnosis, treatment received, major surgery, self‐reported health status, income, and education.^18^ In multivariable analysis, patients who identified as Black (OR 0.47; CI 0.23, 0.90) were associated with greater likelihood of nonadherence to survivorship care guidelines, after adjusting for insurance type, age, sex, and cost of recommended procedures.^20^	There was not a significant association for those of Hispanic ethnicity in predicting an initial survivorship visit, after adjusting for sex, therapeutic modalities, current age, insurance status, and distance from the clinic.^23^ Race was not a significant predictor of follow‐up at 5 and 10 years (adjusting for clustering of institutions).^36^ Survivors who were non‐Hispanic Black, Hispanic, or other races/ethnicities (in comparison with those who were non‐Hispanic White) did not experience a significant association with survivorship clinic attendence, after adjusting for sex, age at diagnosis, and insurance.^38^ In multivariable analysis and in comparison with privately insured Whites, publicly insured Blacks and uninsured, privately insured, or publicly insured Hispanic/Latinxs were not significantly associated with forgoing care, after adjusting for current age, sex, treatment era, cancer diagnosis, treatment received, major surgery, and self‐reported health status.^18^	There evidence of disparities in survivorship care engagement for survivors of non‐White race or Hispanic ethnicity.	High Reason for downgrading: Not applicable
Current age	1 Prospective study: Crom, 2007^17^ 12 Retrospective observational studies: Benedict, 2021^20^ Berg, 2016^21^ Daly, 2019^23^ Johnson, 2004^25^ McBride, 2011^27^ Michel, 2011^28^ Milam, 2015^29^ Oeffinger, 2004^31^ Reppucci, 2017^32^ Streefkerk, 2019^33^ vanLaar, 2013^35^ Welch, 2017^36^	Favors older current age: Those who were ages 6–11 (HR 1.55; CI 1.24, 1.93) or 12–17 (HR 1.44; CI 1.14, 1.83) were more likely to have an initial survivor clinic visit compared to those who were between the ages of 2 and 5, after adjusting for sex, race/ethnicity, therapeutic modalities, insurance status, and distance from the clinic.^23^ In comparison with those <20 years, survivors aged 20 years or older had a significantly higher number of contacts with their PCP (20–29 years contact rate ratio 1.23; CI 1.00, 1.53; 30–39 years 1.53; 1.23, 1.90; ≥40 years 1.58; CI 1.23, 2.04), after adjusting for sex, attained age, and treatment received.^33^ In multivariable analysis, survivors ages 20–34 (RR 1.40, CI 1.2, 1.7) and those ≥35 years of age (RR 1.40, CI 1.1, 1.8) were more likely to have 10 or more PCP visits than younger survivors ages 5–19 were, with a significant trend with increasing age (*p* = 0.003). In multivariable analysis, survivors ages 20–34 (RR 4.57, CI 3.3, 6.1) and those ≥35 years of age (RR 3.01, CI 1.9, 4.6) were more likely to have a visit with an oncologist than younger survivors ages 5–19 were, with a significant trend with increasing age (*p* < 0.001). Both models controlled for sex, socioeconomic status, residence in a metropolitan/large community/small community/rural area, time since diagnosis, diagnosis, age at diagnosis, relapse status, second malignancy, and treatment.^27^ Does not favor older current age: In multivariable analysis, older age (OR 0.97; CI 0.94, 1.0) was related to greater likelihood of nonadherence, after adjusting for race, insurance type, sex, and cost of recommended procedures.^20^ The odds of difficulty obtaining care increased 3% (OR 1.03; CI 1.00, 1.06) for every year of age at survey, after adjusting for sex, marital status, employment, age at diagnosis, cancer diagnosis, and radiation therapy.^17^ In the multivariable model, older current age was associated with less than annual healthcare provider visits (OR 1.35; CI 1.11, 1.63), after adjusting for chemotherapy exposure, sex, and insurance coverage.^21^ In comparison with those currently under the age of 25, those of ages 25–29 (OR 0.66; CI 0.42, 1.03), 30–34 (OR 0.27; CI 0.15, 0.49), and 35+ (OR 0.24; CI 0.12, 0.49) were less likely to attend survivorship care, after adjusting for health beliefs (susceptibility, severity, benefits, barriers, health value, cues to action), demographics (sex, living in a relationship, education, employment, immigration status, language spoken), and medical variables (age at diagnosis, diagnostic category, treatment, SCT, relapse, medical report received, follow‐up checklist received).^28^ For each year increase in current age at survey, survivors were less likely (OR 0.83; CI 0.79, 0.86) to access survivorship care, after adjusting for socioeconomic status at diagnosis, socioeconomic status at survey, age at diagnosis, education, treatment intensity, sex, race/ethnicity, insurance.^29^, ^50^ Survivors 21 years of age or older were associated with decreased odds of having a recent survivorship visit (OR 0.32; CI 0.13, 0.79), after adjusting for treatment intensity, time since diagnosis, sex, health insurance status, Spanish‐speaking Hispanic parent, English‐speaking Hispanic parent, and English‐speaking non‐Hispanic parent.^29^, ^52^ In the multivariable model and in comparison with those currently 18–20 years of age, survivors ages 21–25 (OR 0.65, 0.50–0.85), 26–30 (OR 0.32, 0.22–0.48), and 31–39 (OR 0.35, 0.24–0.50) were less likely to access survivorship care, after adjusting for years since diagnosis, sex, race and ethnicity, socioeconomic status, health insurance coverage, high levels of depressive symptoms, number of late effects, treatment intensity, received a written treatment summary, reported having a doctor for regular (non‐cancer) care, discussed cancer‐related follow‐up care with a doctor in the last 2 years, knowledge of the need for life‐long survivorship care, health care self‐efficacy, and family influence of health care decisions.^29^, ^46^ In comparison with survivors 18–19 years of age at study, those who were ages 25–29 (OR 1.67; CI 1.38, 2.02), 30–34 (OR 1.74; CI 1.42, 2.14), and 35 years of age or more (OR 2.29; CI 1.83, 2.87) were more likely to report absence of a cancer‐related medical visit, after adjusting for sex, ethnicity, age at interview, educational attainment, health insurance, health status, concern for future heatlh, and high‐risk treatment. In comparison with survivors 18–19 years of age at study, those who were ages 20–24 (OR 1.31; CI 1.07, 1.61), 25–29 (OR 1.88; CI 1.51, 2.34), 30–34 (OR 2.79; CI 2.19, 3.56), and 35 years of age or more (OR 3.43; CI 2.61, 4.51) were more likely to report absence of a cancer center medical visit, after adjusting for sex, ethnicity, educational attainment, health insurance, health status, concern for future heatlh, and high‐risk treatment.^31^	There was not a significant relationship for those age 18 or older in predicting an initial survivorship visit, after adjusting for sex, race/ethnicity, therapeutic modalities, insurance status, and distance from the clinic, in comparison with those who were between the ages of 2 and 5.^23^ In the multivariable analysis, current age was not significantly associated with attending survivorship visits, when adjusting for current age, employment, and socioeconomic status.^25^ Current age at the time of follow‐up was not significantly associated with 10‐year follow‐up (adjusting for clustering of institutions).^36^	Most of the evidence indicated that older age is associated with a disparity in survivorship care and general care engagement.	Moderate Reason for downgrading: Inconsistency^a^
		For each 1‐year increase in age at study, survivors experienced an increased odds (OR 1.03; CI 1.02, 1.04) of having general care (rather than risk‐based survivorship care), after adjusting for sex, race/ethnicity, annual household income, educational attainment, employment status, insurance coverage, poor emotional health, cancer‐related anxiety, cancer‐related pain, poor physical health, and grade of chronic disease.^31^, ^58^ Those of older current age had reduced odds of adherence as compared with younger subjects (10‐year increase: OR 0.66; CI 0.50, 0.89) and those <18 years of age had greater odds of adherence as compared with subjects 18 years of age or older (OR 1.53; CI 1.04, 2.25). Survivors younger than 18 years of age had greater odds of echocardiogram adherence as compared with those 18 years and over (OR 2.06, CI 1.02, 4.16). Both used multivariable models adjusting for sex, treatment with radiation, treatment with an anthracycline, treatment with a transplant, and age at diagnosis.^32^ Older participants were less likely to attend all clinic appointments (*p* < 0.05).^35^ At 5 years from diagnosis, patients were less likely to participate in follow‐up if they were over age 18 at the time of follow‐up (OR 0.37, CI 0.15, 0.91; controlling for clustering of institutions).^36^			
Employment	1 Prospective study: Crom, 2007^17^ 4 Retrospective observational studies: Berg, 2016^21^ Johnson, 2004^25^ Michel, 2011^28^ Oeffinger, 2004^31^	Favors those currently employed: Those who were currently employed experienced a decrease in the odds of difficulty obtaining care (OR 0.52; CI 0.36, 0.75) compared to those who were unemployed, after adjusting for sex, age at survey, marriage, age at diagnosis, cancer diagnosis, and radiation therapy.^17^ In comparison with employed survivors, unemployed survivors were less likely to report general care rather than risk‐based survivorship care (0.7; CI 0.6, 0.8), after adjusting for sex, race/ethnicity, current age, age at interview, annual household income, educational attainment, insurance coverage, poor emotional health, cancer‐related anxiety, cancer‐related pain, poor physical health, and grade of chronic disease.^31^, ^58^	Employment status was not significantly associated with annual healthcare provider visits, after adjusting for current age, insurance coverage, and chemotherapy exposure.^21^ In the multivariable analysis, employment status was not significantly associated with attending survivorship visits in the multivariable model adjusting for current age, time since treatment completion, and socioeconomic status.^25^ Employment status was not significantly associated with survivorship care, after adjusting for health beliefs (susceptibility, severity, benefits, barriers, health value, cues to action), demographics (sex, age at study, living in a relationship, education, immigration status, language spoken, age at study), and medical variables (age at diagnosis, diagnostic category, treatment, SCT, relapse, medical report received, follow‐up checklist received).^28^	Being unemployed was not associated with a disparity in survivorship care engagement and there were mixed results for other care outcomes.	Moderate Reason for downgrading: Inconsistency^a^ ^,^ ^d^
Cancer treatment (e.g., type, intensity)	1 Prospective study: Crom, 2007^17^ 14 Retrospective observational studies: Barakat, 2012^19^ Berg, 2016^21^ Daly, 2019^23^ Johnson, 2004^25^ May, 2017^26^ McBride, 2011^27^ Michel, 2011^28^ Milam, 2015^29^ Nathan, 2016^30^ Oeffinger, 2004^31^ Reppucci, 2017^32^ Streefkerk, 2019^33^ Welch, 2017^36^ Zheng, 2016^38^	Favors those treated with chemotherapy: In multivariable analysis, survivors who received any chemotherapy (RR 1.85; 1.0, 3.3), chemotherapy/surgery (RR 2.30; CI 1.3, 4.1), chemotherapy/radiation (RR 2.43, CI 1.3, 4.0), radiation/surgery (RR 3.5; CI 2.0, 6.0), or chemotherapy/radiation/surgery (RR 3.95; CI 2.2, 7.1) were more likely to have an oncologist visit compared with survivors who received only surgery (adjusting for sex, socioeconomic status, residence in a metropolitan/large community/small community/rural area, current age, time since diagnosis, diagnosis, age at diagnosis, relapse status, and second malignancy).^27^ Does not favor those treated with chemotherapy: In the multivariable model, exposure to chemotherapy was associated with less than annual healthcare provider visits (OR 5.73; CI 0.98, 40.30), after adjusting for current age, sex, and insurance coverage.^21^ Favors those treated with surgery: Those treated with surgery only (contact RR 1.23; CI 1.00, 1.51) had more PCP contact during follow‐up compared with those treated with chemotherapy only, when adjusting for sex and attained age.^33^ Survivors who received treatment with surgery only (HR 0.02; CI 0.00, 0.13) were less likely to attend survivorship clinic in comparison with those who received treatment with surgery, chemotherapy, and radiation, after adjusting for sex, age at diagnosis, and insurance status.^38^ Does not favor those treated with surgery: Those who received surgery only (HR 0.04; CI 0.02, 0.08) were less likely to have an initial survivor clinic visit compared to those who received chemotherapy only, after adjusting for sex, race/ethnicity, therapeutic modalities, current age, insurance status, and distance from the clinic.^23^ In comparison with those treated with chemotherapy only, those treated with surgery only (OR 0.44; CI 0.20, 0.99) were less likely to attend survivorship care visits, after adjusting for health beliefs (susceptibility, severity, benefits, barriers, health value, cues to action), demographics (sex, age at study, living in a relationship, education, employment, immigration status, language spoken), and medical variables (age at diagnosis, diagnostic category, treatment, SCT, relapse, medical report received, follow‐up checklist received).^28^ In multivariable analyses, survivors treated with surgery alone (in comparison with chemotherapy alone) were more likely to be lost to follow‐up for more than 1000 days and not attend survivorship clinic (OR 6.70; CI 3.10, 14.90), after adjusting for cancer diagnosis, race, insurance, and age at diagnosis.^26^ Favors those treated with radiation: In comparison with survivors who did not receive radiation, those who did receive radiation to the brain (OR 0.5; CI 0.4, 0.6), chest (OR 0.3; CI 0.2, 0.4), other (OR 0.5; CI 0.4, 0.6), or unknown sites (OR 0.5; CI 0.4, 0.8) were less likely to report general care versus risk‐based survivorship care, after adjusting for sex, current age, and age at interview.^31^, ^58^ Those who received radiation therapy had greater odds of DXA scan adherence (OR 2.60; CI 1.39, 4.88) as compared with those who did not receive radiation, after adjusting for sex, anthracycline exposure, transplant, age at diagnosis, and age at procedure.^32^ Those treated with radiotherapy only (contact RR 1.49; CI 1.15, 1.93) had more PCP contact during follow‐up compared with those treated with chemotherapy only, when adjusting for sex and attained age.^33^ Survivors who received radiation to the head/neck (HR 1.88; CI 1.28, 2.77), chest (HR 2.72; CI 1.71, 4.34), or abdomen/pelvis (HR 1.78, CI 1.01, 3.13) were more likely to attend survivorship clinic in comparison with those who received no radiation, after adjusting for sex, age at diagnosis, and insurance status.^38^ Does not favor those treated with radiation: Those who received radiation only (HR 0.24; CI 0.15, 0.39) were less likely to have an initial survivor clinic visit compared to those who received chemotherapy only, after adjusting for sex, race/ethnicity, therapeutic modalities, current age, insurance status, and distance from the clinic.^23^	Treatment with radiation did not have a significant association with difficulty obtaining care, after adjusting for sex, age at survey, marriage, employment status, insurance coverage, cancer diagnosis, and age at diagnosis.^17^ In the multivariable analysis, time since treatment completion was not significantly associated with attending survivorship visits, when adjusting for current age, employment, and socioeconomic status.^25^ In multivariable analysis, type of treatment received was not significantly associated with ≥10 PCP visits or specialist visits, when adjusting for sex, socioeconomic status, residence in a metropolitan/large community/small community/rural area, time since diagnosis, current age, age at diagnosis, cancer diagnosis, relapse status, and second malignancy.^27^ Treatment with anthracycline or transplant were not significantly associated with recommendation adherence, after adjusting for sex, anthracycline exposure, transplant, age at diagnosis, and age at procedure recommendation.^32^ Clinical trial participation (enrolled on a treatment study) was not significantly associated with 5‐ or 10‐year follow‐up (adjusting for clustering of institutions).^36^	Across studies, there was evidence that survivors who received less intense treatment (guided by the Intensity of Treatment Rating Scale) or single‐modality regimens were less likely to be engaged in survivorship care and general care; however, there were some conflicting evidence with studies finding no effect of treatment.	Low Reason for downgrading: Inconsistency^a^ Study limitation^b^
		Favors those treated with SCT: In comparison with those who did not have a SCT, those who did have a SCT (OR 2.83; CI 1.21, 6.58) were more likely to attend survivorship care visits, after adjusting for health beliefs (susceptibility, severity, benefits, barriers, health value, cues to action), demographics (sex, age at study, living in a relationship, education, employment, immigration status, language spoken), and medical variables (age at diagnosis, diagnostic category, treatment, relapse, medical report received, follow‐up checklist received).^28^ Does not favor those treated with SCT: In multivariable analyses, those who received treatment with a SCT (in comparison with no transplant) were more likely to be lost to follow‐up for more than 1000 days and not attend survivorship clinic (OR 2.0; CI 1.04, 3.70), after adjusting for diagnosis, race, insurance, and age at diagnosis.^26^ Favors those treated with high intensity regimens according to the Intensity of Treatment Rating Scale: For each increase in the level of treatment intensity (from 1 to 4), survivors were more likely (OR 1.83; CI 1.09, 3.06) to access survivorship care, after adjusting for sex, race/ethnicity, health insurance, family influence on healthcare decisions, having a regular doctor, healthcare self‐efficacy, and post‐traumatic growth.^29^ Greater treatment intensity was associated with increased odds of having a recent survivorship visit (OR 1.89; CI 1.07, 3.31), after adjusting for current age, time since diagnosis, sex, health insurance status, Spanish‐speaking Hispanic parent, English‐speaking Hispanic parent, and English‐speaking non‐Hispanic parent.^29^, ^52^ Increased survivorship clinic attendance was associated with treatment intensity score of 2 (RR 1.68, CI 1.26, 2.25), score of 3 (RR 2.21; CI 1.65, 2.96), or score of 4 (RR 1.69; CI 1.10, 2.58), compared to those with a score of 1 (least intense treatment); higher cyclophosphamide equivalent doses as those who received 4000–7999 mg/m^2^ (RR 1.29; CI 1.08, 1.54) or those who received >8000 mg/m^2^ (RR 1.47; CI 1.25, 1.73), compared to those who did not receive cyclophosphamide; and radiation to the brain (RR 1.44; CI 1.23, 1.67), chest (RR 1.59; CI 1.28, 1.97), or other sites (RR 1.31; CI 1.13, 1.53), compared to those who did not receive radiation. Models were adjusted for sex, age at diagnosis, socioeconoomic status, cancer diagnosis, cyclophosphamide equivalent dose, doxorubicin equivalent dose, radiation, secondary malignancy, or relapse before index date, survivorship clinic model, distance to survivorship clinic, and complete history/physical exam by a PCP.^30^ In comparison with survivors who did not receive high‐risk treatment, those who did receive high‐risk treatment were less likely to report absence of cancer‐related medical visits (OR 0.59; CI 0.52, 0.65) or cancer center medical visits (OR 0.45, CI 0.39, 0.51), after adjusting for age at study, sex, ethnicity, educational attainment, insurance coverage, health status, and concern for future health.^31^ In comparison with survivors who did not receive treatment with anthracyclines, those who received chest radiation (OR 0.4; CI 0.3, 0.6) and those who received anthracyclines and chest radiation (OR 0.5; CI 0.4, 0.6) were less likely to report general care versus risk‐based survivorship care, after adjusting for sex, current age, and age at interview. In comparison with survivors who did not receive treatment with alkylating agents, those who received the highest dose in the third tertile (OR 0.6; CI 0.4, 0.9) were less likely to report general care versus risk‐based survivorship care, after adjusting for sex, current age, and age at interview.^31^ Survivors who received surgery and chemotherapy (HR 0.54; CI 0.30, 0.97) were less likely to attend survivorship clinic in comparison with those who received treatment with surgery, chemotherapy, and radiation; however, survivors who received alkylating agents (HR 2.28; CI 1.58, 3.30), anthracyclines (HR 3.05; CI 2.09, 4.44), or lung toxic therapies (HR 1.89; CI 1.19, 3.00) were more likely to attend survivorship clinic in comparison with those who received no chemotherapy; after adjusting for sex, age at diagnosis, and insurance status.^38^ Does not favor those with more time lapsed since treatment completion: Patients who were off treatment for a longer period of time were about half as likely (OR 0.44, *p* = 0.004) to attend a follow‐up or survivorship visit in comparison with those who more recently completed treatment.^19^	Survivors who enrolled on a clinical trial (in comparison with did not enroll) or who did not experience a relapse (in comparison with those who did relapse) did not experience a significant association with survivorship clinic attendence, after adjusting for sex, age at diagnosis, and insurance status.^38^		
Sex	1 Prospective study: Crom, 2007^17^ 13 Retrospective observational studies: Barakat, 2012^19^ Benedict, 2021^20^ Berg, 2016^21^ Daly, 2019^23^ McBride, 2011^27^ Michel, 2011^28^ Milam, 2015^29^ Nathan, 2016^30^ Oeffinger, 2004^31^ Streefkerk, 2019^33^ Welch, 2017^36^ Zanetti, 2022^37^ Zheng, 2016^38^	Favors females: In univariable analysis, females were twice as likely as males to attend a survivorship clinic visit 5 years post‐diagnosis (OR 2.20, *p* = 0.077).^19^ In multivariate regression, male sex (OR 0.09; CI 0.03, 0.35) was associated with less than annual healthcare provider visits, after adjusting for current age, insurance coverage, and chemotherapy exposure.^21^ In multivariable analysis, female survivors were more likely to have 10 or more PCP visits (RR 1.78; CI 1.5, 2.1), more likely to visit specialists (RR 1.15; CI 1.0, 1.3), and more likely to have seen an oncologist (RR 1.40; CI 1.1, 1.7) than male survivors (controlling for socioeconomic status, residence in a metropolitan/large community/small community/rural area, current age, time since diagnosis, diagnosis, age at diagnosis, relapse status, second malignancy, and treatment).^27^ Female survivors were more likely (OR 1.34; CI 1.11, 1.62) to access survivorship care, after adjusting for socioeconomic status at diagnosis, socioeconomic status at survey, age at diagnosis, education, treatment intensity, race/ethnicity, age at survey, and insurance.^29^, ^50^ Female survivors were more likely to have a recent survivorship visit (OR 4.26; CI 1.60, 11.40), after adjusting for current age, time since diagnosis, treatment intensity, health insurance status, Spanish‐speaking Hispanic parent, English‐speaking Hispanic parent, and English‐speaking non‐Hispanic parent.^29^, ^52^ Females were more likely to attend survivorship clinic (RR 1.18; CI 1.07, 1.31), after adjusting for age at diagnosis, socioeconoomic status, cancer diagnosis, diagnosis prior to 1999, treatment intensity, cyclophosphamide equivalent dose, doxorubicin equivalent dose, radiation, secondary malignancy or relapse before index date, survivorship clinic model, distance to survivorship clinic, and complete history/physical exam by a PCP.^30^ Females had more contacts with their PCP (contact ratio 1.96; CI 1.72, 2.23) in comparison with males, after adjusting for sex, attained age, and treatment received.^33^ Does not favor females: The odds of difficulty obtaining care decreased for males in the prior year (OR 0.59; CI 0.41, 0.85) compared to females, after adjusting for age at survey completion, marital status, employment status, age at diagnosis, cancer diagnosis, and radiation therapy.^17^	In multivariable analysis, male sex was not significantly related to nonadherence, after adjusting for race, age, insurance type, and cost of recommended procedures.^20^ There was not a significant relationship by sex in predicting an initial survivorship visit, after adjusting for race/ethnicity, therapeutic modalities, current age, insurance status, and distance from the clinic.^23^ Sex was not significantly associated with survivorship care, after adjusting for health beliefs (susceptibility, severity, benefits, barriers, health value, cues to action), demographics (age at study, living in a relationship, education, employment, immigration status, language spoken), and medical variables (age at diagnosis, diagnostic category, treatment, SCT, relapse, medical report received, follow‐up checklist received).^28^ Multivariable model predicting general care (in comparison with risk‐based survivor‐focused care) was not significantly associated with male sex, after adjusting for race/ethnicitiy, current age, annual household income, educational attainment, employment status, health insurance, poor emotional health, cancer‐related anxiety, cancer‐related pain, poor physical health, and chronic disease status.^31^, ^58^	There is evidence that male sex is associated with a disparity in survivorship care engagement.	Low Reason for downgrading: Inconsistency^a^ ^,^ ^e^ Study limitation^b^
			Sex was not a significant predictor of follow‐up at 5 and 10 years (adjusting for clustering of institutions).^36^ In multivariable analysis, sex was not a significant predictor of echocardiogram screening or mental health visit, after adjusting for age, sponsor rank group, and type of care (military vs. civilian).^37^ There was not a significant association with survivorship clinic attendance by sex, after adjusting for age at diagnosis and insurance.^38^		
Cancer diagnosis, grade, stage, or relapse	1 Prospective study: Crom, 2007^17^ 12 Retrospective observational studies: Barakat, 2012^19^ Berkman, 2019^22^ Daly, 2019^23^ May, 2017^26^ McBride, 2011^27^ Michel, 2011^28^ Nathan, 2016^30^ Oeffinger, 2004^31^ Reppucci, 2017^32^ Streefkerk, 2019^33^ Welch, 2017^36^ Zheng, 2016^38^	Favors those who experienced a relapse: In multivariable analysis, survivors who had relapsed (RR 2.11; CI 1.4, 3.3) had an increased likelihood of oncologist visits compared with survivors of acute lymphoblastic leukemia; controlled for sex, socioeconomic status, residence in a metropolitan/large community/small community/rural area, time since diagnosis, current age, age at diagnosis, relapse status, second malignancy, and treatment).^27^ In comparison with those who did not experience a relapse, those who did experience a relapse (OR 2.78; CI 1.70, 4.56) were more likely to attend survivorship care, after adjusting for health beliefs (susceptibility, severity, benefits, barriers, health value, cues to action), demographics (sex, age at study, living in a relationship, education, employment, immigration status, language spoken), and medical variables (age at diagnosis, diagnostic category, treatment, SCT, medical report received, follow‐up checklist received).^28^ Favors those who were diagnosed with a bone tumor or sarcoma: In multivariable analysis, survivors of bone tumors (RR 3.89; CI 1.9, 7.8) had an increased likelihood of oncologist visits compared with survivors of acute lymphoblastic leukemia; controlled for sex, socioeconomic status, residence in a metropolitan/large community/small community/rural area, time since diagnosis, current age, age at diagnosis, relapse status, second malignancy, and treatment).^27^ After adjusting for sex, age at diagnosis, and insurance status, survivors diagnosed sarcoma (HR 3.30; CI 1.47, 7.42) were more likely to attend survivorship clinic in comparison with those diagnosed with central nervous system tumors.^38^ Does not favor those diagnosed with bone cancer: In comparison with survivors' who were diagnosed with bone cancer, survivors who were diagnosed with central nervous system (7.25; 1.25, 42.23), Wilms tumor (6.67; 1.24, 36.02), or neuroblastoma (8.30; 1.33, 51.96) experienced an increased likelihood of reporting physician‐based skin exam, after adjusting for sex, education, age (years), race/ethnicity, skin type, age at diagnosis, diagnosis, chemotherapy (yes/no), highest CTCAE grade chronic condition, maximum radiotherapy dose, patient activation.^31^, ^74^ Favors those who were diagnosed with leukemia or lymphoma: In comparison with those who were diagnosed with a hematologic malignancy, those with a solid tumor experienced a increase in the odds of difficulty obtaining care (OR 1.84, CI 1.25, 2.71) compared to those who were diagnosed with a hematologic malignancy, after adjusting for sex, age at survey, marriage, employment status, insurance coverage, age at diagnosis, and radiation therapy.^17^ After adjusting for sex, age at diagnosis, and insurance status, survivors diagnosed with leukemia (HR 3.36; CI 1.65, 6.83) or lymphoma (HR 3.99; CI 1.87, 8.53) were more likely to attend survivorship clinic in comparison with those diagnosed with central nervous system tumors.^38^ Does not favor those who were diagnosed with leukemia or lymphoma: At 10 years post‐diagnosis, leukemia with CNS involvement was negatively associated (OR 0.26; CI 0.081, 0.82) with follow‐up care, after controlling for clustering of institutions.^36^	Tumor grade was not significantly associated with missed visits, when adjusting for race and insurance.^22^ Type of cancer diagnosis and an additional cancer event (e.g., any recorded relapse, progression, or subsequent malignancy following initial cancer diagnosis) were not significantly associated with an initial survivorship visit, after adjusting for sex, race/ethnicity, therapeutic modalities, insurance status, distance from the clinic, and current age.^23^ Cancer diagnosis (leukemia, lymphoma, CNS tumor, solid tumor) was not significantly associated with lack of survivorship care in the multivariable model, which controlled for treatment (chemotherapy, radiation, surgery, chemotherapy/radiation, chemotherapy/surgery, radiation/surgery, chemotherapy/radiation/surgery), SCT, race/ethnicity (White, Black, Hispanic, other), and lack of insurance at last visit, which were all significant.^26^		
		Does not favor those diagnosed with a brain tumor or other cancer: In univariate analysis, those diagnosed with brain tumors (OR 0.24, *p* = 0.055) were less likely to attend a survivorship visit compared to patients with leukemia or lymphoma.^19^ Decreased survivorship clinic attendance was associated with a diagnosis of a brain tumor (RR 0.63; CI 0.50, 0.77) or other cancer (RR 0.67; CI 0.54, 0.84) compared to ALL, after adjusting for sex, age at diagnosis, socioeconomic status, diagnosis prior to 1999, treatment intensity, cyclophosphamide equivalent dose, doxorubicin equivalent dose, radiation, secondary malignancy or relapse before index date, survivorship clinic model, distance to survivorship clinic, and complete history/physical exam by a PCP.^30^	Cancer diagnosis was not significantly associated with survivorship care, after adjusting for health beliefs (susceptibility, severity, benefits, barriers, health value, cues to action), demographics (sex, age at study, living in a relationship, education, employment, immigration status, language spoken), and medical variables (age at diagnosis, treatment, SCT, relapse, medical report received, follow‐up checklist received).^28^ Cancer diagnosis was not associated with ≥10 PCP visits or specialist visits, when adjusting for sex, socioeconomic status, residence in a metropolitan/large community/small community/rural area, time since diagnosis, current age, age at diagnosis, relapse status, second malignancy, and treatment. Second malignancy was not associated with ≥10 PCP visits, specialist visits, or oncologists visits, when adjusting for sex, socioeconomic status, residence in a metropolitan/large community/small community/rural area, time since diagnosis, current age, age at diagnosis, cancer diagnosis, relapse status, and treatment.^27^ Cancer diagnosis was not significantly associated with adherence, after adjusting for sex, radiation exposure, anthracycline exposure, transplant, age at procedure recommendation, age at diagnosis.^32^	There was no clear pattern for disparities in cancer diagnoses and experiencing a relapse. Cancer diagnosis (e.g., leukemia vs. solid tumors) may be associated with disparities but the exact pattern remains unclear.	Low Reason for downgrading: Inconsistency^a^
			Cancer diagnosis was not significantly associated with the number of contacts with the PCP, when adjusting for sex and current age.^33^ Survivorship clinic attendance was not statistically associated with diagnosis with a secondary malignancy or relapse for index date, after adjusting for sex, age at diagnosis, socioeconoomic status, cancer diagnosis, cyclophosphamide equivalent dose, doxorubicin equivalent dose, radiation, secondary malignancy or relapse before index date, survivorship clinic model, distance to survivorship clinic, and complete history/physical exam by a PCP.^30^ CNS involvement of leukemia was not significantly associated with 5‐year follow‐up. High‐risk leukemia and relapsed leukemia were not significantly associated with 5‐ or 10‐ year follow‐up (after controlling for clustering of institutions).^36^ Those diagnosed with thyroid cancer/melanoma or other solid tumors did not have a significant association with attendence at survivorship clinic, after adjusting for sex, age at diagnosis, and insurance, in comparison with those diagnosed with central nervous system tumors.^38^		
Age at diagnosis	1 Prospective study: Crom, 2007^17^ 10 Retrospective observational studies: May, 2017^26^ McBride, 2011^27^ Michel, 2011^28^ Milam, 2015^29^ Nathan, 2016^30^ Oeffinger, 2004^31^ Reppucci, 2017^32^ Szalda, 2016^34^ Zanetti, 2022^37^ Zheng, 2016^38^	Favors older age at diagnosis: For each 1‐year increase in age at diagnosis, survivors experienced a decreased odds (OR 0.97; CI 0.95, 0.97) of having general care (thus more likely to have risk‐based survivorship care), after adjusting for sex, race/ethnicity, current age, age at interview, annual household income, educational attainment, employment status, insurance coverage, poor emotional health, cancer‐related anxiety, cancer‐related pain, poor physical health, and grade of chronic disease.^31^, ^58^ Does not favor older age at diagnosis: In multivariable analyses, older age at diagnosis (ages 5–9 OR 1.8; CI 1.1, 3.0; ages 10–14 OR 3.3; 1.8, 6.1; and ages 15 and above: OR 4.8; 2.1, 11.7; in comparison with those ages 0–4) was more likely to be lost to follow‐up for more than 1000 days and not attend survivorship clinic, when adjusting for diagnosis, race, insurance, treatment exposure, and SCT exposure (global overall *p*‐value <0.001).^26^ In comparison with those ages 0–4 at diagnosis, those of ages 8–11 (OR 2.06; CI 1.18, 3.61) or 12+ years of age (OR 4.41; 2.56, 7.60) were more likely to attend survivorship care, after adjusting for health beliefs (susceptibility, severity, benefits, barriers, health value, cues to action), demographics (sex, age at study, living in a relationship, education, employment, immigration status, language spoken), and medical variables (diagnostic category, treatment, SCT, relapse, medical report received, follow‐up checklist received).^28^ For each year increase in age at diagnosis, survivors were more likely (OR 1.12; CI 1.08, 1.16) to access survivorship care, after adjusting for socioeconomic status at diagnosis, socioeconomic status at survey, education, treatment intensity, sex, race/ethnicity, age at survey, insurance.^29^, ^50^ For each year increase in age at diagnosis, survivors were less likely (OR 0.88; CI 0.84, 0.92) to access survivorship care, after adjusting for current age, sex, race and ethnicity, socioeconomic status, health insurance coverage, high levels of depressive symptoms, number of late effects, treatment intensity, received a written treatment summary, reported having a doctor for regular (non‐cancer) care, discussed cancer‐related follow‐up care with a doctor in the last 2 years, knowledge of the need for life‐long survivorship care, health care self‐efficacy, and family influence of health care decisions.^29^, ^46^ As age at diagnosis increased, adherence with echocardiogram recommendations decreased (10‐year increase: OR 0.59; CI 0.37, 0.95), after adjusting for sex, radiation exposure, anthracycline exposure, transplant, and age at procedure recommendation.^32^ Survivors who were older ages at diagnosis (OR 0.90; CI 0.82, 0.99) were less likely to access survivorship care for each year of age increase, after adjusting for knowledge of risk for second cancer, comfort discussing concerns, and motivation to take care of health.^34^, ^69^ In multivariable analysis, being age 10–19 years at diagnosis was associated with decreased odds of receiving a dual‐energy x‐ray absorptiometry (DEXA) scan (OR 0.32; CI 0.11, 0.95) and mental health visits (OR 0.28; CI 0.11–0.70), respectively, after adjusting for sex, sponsor rank group, and type of care (military vs. civilian).^37^	Age at diagnosis did not have a significant association with difficulty obtaining care, after adjusting for sex, age at survey, marriage, employment status, insurance coverage, cancer diagnosis, and radiation therapy.^17^ Survivorship clinic attendance was not statistically associated with age at diagnosis, after adjusting for sex, socioeconoomic status, cancer diagnosis, cyclophosphamide equivalent dose, doxorubicin equivalent dose, radiation, secondary malignancy or relapse before index date, survivorship clinic model, distance to survivorship clinic, and complete history/physical exam by a PCP.^30^ Age at diagnosis was not significantly associated with adherence, after adjusting for sex, radiation exposure, anthracycline exposure, transplant, and age at procedure recommendation.^32^ In multivariable analysis, age group was not a significant predictor of echocardiogram screening, after adjusting for sex, sponsor rank group, and type of care (military vs. civilian).^37^ There was not a significant association with survivorship clinic attendence by age at diagnosis, after adjusting for sex and insurance.^38^ Age at diagnosis was not associated with ≥10 PCP, specialist, or oncologists visits, when adjusting for sex, socioeconomic status, residence in a metropolitan/large community/small community/rural area, time since diagnosis, current age, age at diagnosis, cancer diagnosis, relapse status, second cancer diagnosis, and treatment.^27^	The evidence indicated that older age at diagnosis is associated with a disparity in survivorship care and general care engagement.	Low Reason for downgrading: Inconsistency^a^
Geographic area (e.g., underserved, nonmetropolitan, rural)	1 Prospective study: Crom, 2007^17^ 6 Retrospective observational studies: Barakat, 2012^19^ Daly, 2019^23^ McBride, 2011^27^ Nathan, 2016^30^ Oeffinger, 2004^31^ Zheng, 2016^38^	Favors those living in non‐metropolitan areas: In multivariable analysis, survivors from a small community (in comparison with a metropolitan designated area) were more likely to have a visit with an oncologist (RR 1.45, CI 1.0, 2.0; controlling for sex, socioeconomic status, sex, current age, time since diagnosis, diagnosis, age at diagnosis, relapse status, second malignancy, and treatment).^27^ Does not favor those who travel far distances: In univariable analysis, patients who lived farther than 57.6 km to the hospital were less likely (OR 0.24, *p* = 0.003) to attend a follow‐up or survivorship visit as patients who lived closer.^19^ Those who lived 25–50 miles from the clinic (HR 0.76; CI 0.63, 0.93) or those who lived more than 50 miles from the clinic (HR 0.67; CI 0.54, 0.82) were significantly less likely to have an initial survivor clinic visit compared to those who lived less than 25 miles from the clinic, after adjusting for sex, race/ethnicity, therapeutic modalities, current age, and insurance status.^23^ Those who traveled 25–49 km (RR 0.88; CI 0.76, 1.01), 50–99 km (RR 0.77, CI 0.65–0.91), >100 km (RR 0.48; CI 0.39, 0.60) had a decreased likelihood of survivorship clinic attendance, after adjusting for sex, age at diagnosis, socioeconomic status, cancer diagnosis, diagnosis prior to 1999, treatment intensity, cyclophosphamide equivalent dose, doxorubicin equivalent dose, radiation, secondary malignancy or relapse before index date, survivorship clinic model, and complete history/physical exam by a PCP.^30^ In multivariable regression and after adjusting for individual factors, the number of Childhood Cancer Survivor Study centers (OR 1.12; CI 1.04, 1.20) and the number of physicians/surgeons (OR 1.06; CI 1.01, 1.11) within the geographic area was associated with greater odds of receiving risk‐based survivor‐focused medical care among U.S. residents.^31^, ^60^ Does not favor those who travel by car: Those who traveled by a car were more likely to be non‐attenders at survivorship clinic (OR 12.74; CI 3.97, 40.86) compared to those who traveled by bus, after adjusting for age, socioeconomic status, race, years from diagnosis, additional cancer event, insurance coverage, distance from hospital, scheduled social work consultation, and type of clinic visit.^17^, ^39^	Survivors who traveled more than 15 min to the hospital (in comparison with those with travel times less than 15 min) did not experience a significant association with survivorship clinic attendence, after adjusting for sex, age at diagnosis, and insurance.^38^	Overall, there seems to be an association between being from an underserved or rural area and experiencing a disparity in survivorship care engagement.	Low Reason for downgrading: Inconsistency^a^
Insurance type	6 Retrospective observational studies: Benedict, 2021^20^ Daly, 2019^23^ Gardner, 2014,^24^ Milam, 2015^29^ Oeffinger, 2004^31^ Welch, 2017^36^	Favors those who are publicly insured: In adjusted models, publicly insured survivors were more likely to report a cancer‐related (OR 1.22; CI 1.11, 1.35) or a cancer center visit (OR 1.41; CI 1.18, 1.70) than privately insured survivors, when adjusting for age, sex, household income, highest level of educational attainment, race/ethnicity, and Grade 3 or 4 chronic condition.^31^, ^61^ In univariable analysis, use of a mental health professional was less likely to be associated with private insurance coverage, in comparison with those with public insurance or uninsured (*p* = 0.040).^24^ Does not favor those who are publicly insured: Those who had Medicaid at diagnosis (HR 0.77; CI 0.64, 0.92) were less likely than those who had private insurance to have had an initial survivor visit, after adjusting for sex, race/ethnicity, therapeutic modalities, current age, and distance from the clinic.^23^ At 5‐ and 10‐year post‐diagnosis, public insurance coverage was negatively associated with follow‐up care (5‐year follow‐up OR 0.58; CI 0.17, 1.0; 10‐year follow‐up OR 0.48; CI 0.22, 0.73), when compared to those with private or military insurance coverage.^36^ Does not favor those who are publicly insured or uninsured: In multivariable analysis, being insured by Medicaid or uninsured (OR 0.59; CI 0.36, 0.96) was related to greater likelihood of nonadherence, after adjusting for race, age, sex, and cost of recommended procedures.^20^ In comparison with those who were privately insured, those who were uninsured were 4.3 times as likely to have no regular provider for cancer‐related follow‐up care (CI 1.9, 9.4), 3.3 times as likely to lack a regular provider for non‐cancer care (CI 1.6, 6.9), 5.3 times as likely to lack both sources of care (CI 2.1, 13.5), 3.9 times as likely to have had no primary care visit (CI 1.8, 8.2), and 4.5 times as likely to have not seen a cancer specialist (CI 2.1, 9.5). In comparison with those who were privately covered, those with public insurance were 2.5 times as likely to report no regular source of primary care (CI 1.1, 5.4) and 2.8 times as likely not to have made a primary care visit in the past 2 years compared with those with private coverage. Both models adjusted for sex, ethnicity, age, socioeconomic status, and treatment intensity.^29^, ^48^		There was no systematic association of insurance type and survivorship care.	Low Reason for downgrading: Inconsistency^a^ Indirectness^c^
Time since diagnosis	3 Retrospective observational studies: Daly, 2019^23^ McBride, 2011^27^ Nathan, 2016^30^	Does not favor those with less time passed since diagnosis: In multivariable analysis, survivors who were 10–14 years since diagnosis (RR 0.53; CI 0.3, 0.9) and those ≥25 years since diagnosis (RR 0.31, CI 0.2, 0.6) were less likely to have a visit with an oncologist than those diagnosed 5–9 years prior were (controlling for socioeconomic status, residence in a metropolitan/large community/small community/rural area, current age, diagnosis, age at diagnosis, relapse status, second malignancy, and treatment).^27^ Decreased survivorship clinic attendance was associated with a diagnosis prior to 1999 (RR 0.74; CI 0.63, 0.86) compared to those diagnosed after 1999, after adjusting for sex, age at diagnosis, socioeconoomic status, cancer diagnosis, treatment intensity, cyclophosphamide equivalent dose, doxorubicin equivalent dose, radiation, secondary malignancy or relapse before index date, survivorship clinic model, distance to survivorship clinic, and complete history/physical exam by a PCP.^30^	Year of diagnosis was not significantly associated with an initial survivorship visit, after adjusting for sex, race/ethnicity, therapeutic modalities, insurance status, and distance from the clinic.^23^ Time since diagnosis (measured in years) was not significantly associated with ≥10 visits with a PCP or a visit with a specialist, after adjusting for sex, socioeconomic status, residence in a metropolitan/large community/small community/rural area, current age, diagnosis, age at diagnosis, relapse status, second malignancy, and treatment.^27^	Most of the evidence indicated that survivors with more time passed since diagnosis were less likely to receive survivorship care and general care engagement.	Low Reason for downgrading: Inconsistency^a^
Education	2 Retrospective observational studies: Michel, 2011^28^ Oeffinger, 2004^31^	Does not favor those with lower educational attainment: In comparison with those with primary school educational attainment (Swiss complementary school, no education), those with secondary school education (vocational training, high school, teacher's seminar; OR 0.52; CI 0.32, 0.85) were less likely to attend survivorship care, after adjusting for health beliefs (susceptibility, severity, benefits, barriers, health value, cues to action), demographics (sex, age at study, living in a relationship, employment, immigration status, language spoken), and medical variables (age at diagnosis, diagnostic category, treatment, SCT, relapse, medical report received, follow‐up checklist received).^28^	There was not a significant relationship between educational attainment and odds of reporting general care rather than risk‐based survivorship care, after adjusting for sex, race/ethnicity, current age, age at interview, annual household income, insurance coverage, poor emotional health, cancer‐related anxiety, cancer‐related pain, poor physical health, and grade of chronic disease.^31^, ^58^	There is some evidence that lower level of educational attainment is associated with a disparity in survivorship care engagement.	Low Reason for downgrading: Study limitation^b^
Income	6 Retrospective observational studies: Benedict, 2021^20^ Johnson, 2004^25^ McBride, 2011^27^	Favors those from higher income areas: Survivors from areas of higher socioeconomic status areas were more likely to attend survivorship clinic (RR 1.27; CI 1.06, 1.53), after adjusting for sex, age at diagnosis, cancer diagnosis, diagnosis prior to 1999, treatment intensity, cyclophosphamide equivalent dose, doxorubicin equivalent dose, radiation, secondary malignancy or relapse before index date, survivorship clinic model, distance to survivorship clinic, and complete history/physical exam by a PCP.^30^ Does not favor those from lower income areas: In the multivariable analysis, those in the least affluent socioeconomic status group were less likely to attend survivorship clinic in comparison with those in the most affluent group (OR 0.31, *p* = 0.009), when adjusting for age, time since end of treatment, and employment status.^25^	Socioeconomic status was not significantly associated with physician visits to ≥10 primary care visits, any specialist visits, or any oncologist visits, after adjusting for sex, rurality, current age, time since diagnosis, cancer diagnosis, age at diagnosis, relapse status, second cancer status, and type(s) of treatment received.^27^	It is unclear whether income is associated with disparities in survivorship care engagement.	Very low Reason for downgrading: Inconsistency^a^ Study limitation^b^
	Nathan, 2016^30^ Oeffinger, 2004^31^ Zheng, 2016^38^	In comparison with survivors reporting current annual household income more than $60,000, survivors who had income less than $20,000 (RR 1.6; CI 1.2, 2.3) or between $20,000 and $39,999 (RR 1.4; CI 1.0, 1.9) who reported some form of medical care at baseline (either survivor‐focused or general) experienced an increased risk of reporting no care at follow‐up, after adjusting for sex, race/ethnicity, chronic disease status, and education.^31^, ^59^ Does not favor adherence to higher cost procedures: In multivariable analysis, patients with recommended procedures that exceeded a median cost of $400 were less likely to be adherent than those with recommended procedures that cost less than $400 (OR 0.32; CI 0.22, 0.46), after adjusting for race, insurance type, age, and sex.^20^	There was not a significant relationship between annual household income and odds of reporting general care rather than risk‐based survivorship care, after adjusting for sex, race/ethnicity, current age, age at interview, insurance coverage, employment status, poor emotional health, cancer‐related anxiety, cancer‐related pain, poor physical health, and grade of chronic disease.^31^, ^58^ Survivors with incomes greater than $68,999 (in comparison with those with incomes less than $69,000) did not experience a significant association with survivorship clinic attendence, after adjusting for sex, age at diagnosis, and insurance.^38^		
Gender	0 Studies	Not applicable	Not applicable	Not applicable	Insufficient

Abbreviations: CI, 95% confidence interval; OR, odds ratio; PCP, primary care provider; RR, relative risk; SCT, stem cell transplant.

^a^ Reason for downgrading, inconsistency: studies report conflicting result or prospective study does not confirm effects reported in retrospective studies.

^b^ Reason for downgrading, study limitation: the evidence statement was exclusively based on retrospective studies.

^c^ Reason for downgrading, indirectness: the identified studies partially addressed the question of interest.

^d^ Three out of five studies found no association, but a prospective study found that those who were employed experienced less difficulty obtaining survivorship care.

^e^ The prospective study shows a negative association with female sex, where retrospective studies report positive associations with female sex with obtaining survivorship care.

### 3.2 Results of syntheses

The summary of evidence and the resulting GRADE category are documented in Table 1. The table is organized by the disparity categories and documents the presence and absence of disparities in all studies assessing the potential disparity source.

#### 3.2.1 High quality of evidence

A total of 11 studies (1 prospective, 10 retrospective) assessed potential disparities by insurance status (e.g., insured or uninsured) among CCS.^17^, ^21^, ^22^, ^23^, ^24^, ^26^, ^29^, ^31^, ^34^, ^36^, ^38^ Of those 11 studies, similar results were found across 9 studies, such that, being uninsured was associated with a disparity in survivorship care (high QOE).^17^, ^21^, ^22^, ^26^, ^29^, ^31^, ^34^, ^36^, ^38^ For example, a prospective study provided evidence that those who were currently insured experienced a decrease in the odds of difficulty obtaining care (OR 0.18; CI 0.12, 0.26) compared to those who were uninsured, after adjusting for sex, age at survey, marriage status, employment status, age at diagnosis, cancer diagnosis, and radiation therapy.^17^ In multivariable analysis adjusting for different variables in retrospective observational studies, being insured led to increased survivorship care engagement, with odds ranging from 1.7 to 4.2.^26^, ^29^, ^31^, ^36^ Similarly, in multivariable analysis of CCS who were uninsured, disparities in survivorship care engagement were demonstrated with odds ranging from 0.04 to 0.28.^21^, ^29^ However, two studies found contradicting results. In a subsequent publication of the prospective study, CCS who were privately insured were more likely to be nonattenders at survivorship clinics (OR 2.36; CI 1.98, 3.79) compared to CCS who were uninsured, after adjusting for age, socioeconomic status, race, years from diagnosis, additional cancer event, means of travel, distance from hospital, scheduled social work consultation, and type of clinic visit.^17^, ^39^ By contrast, in a retrospective study, use of a mental health professional was less likely to be associated with private insurance coverage upon univariable analysis, in comparison with CCS with public insurance or who were uninsured (*p* = 0.040).^24^

In total, nine observational studies (one prospective, eight retrospective) assessed potential disparities in survivorship care for CCS by race or ethnicity.^17^, ^19^, ^22^, ^23^, ^26^, ^29^, ^31^, ^36^, ^38^ Across studies, we found high QOE for disparities in survivorship care engagement for survivors of non‐White race and Hispanic ethnicity. Of the nine studies examining disparities by race or ethnicity, we found seven studies^17^, ^18^, ^20^, ^23^, ^26^, ^29^, ^31^ that identified a disparity experienced by those of non‐White race or Hispanic ethnicity with estimates of lack of engagement odds ranging from 0.47 to 0.79.^20^, ^29^, ^31^ A prospective study reported non‐Whites were nearly two times more likely to not attend survivorship clinic (OR 1.88; CI 1.19, 2.97) compared to Whites, after adjusting for age, socioeconomic status, years from diagnosis, additional cancer event, insurance status, means of travel, distance from hospital, scheduled social work consultation, and type of clinic visit.^17^ Notably, there was no systematic association of race or ethnicity with insurance type (private, public) although the result was based on low QOE. However, one retrospective observational study found favorable outcomes among CCS of non‐White race and Hispanic ethnicity.^31^ Publicly insured Hispanic survivors were more likely to report a cancer‐related survivorship visit (OR 1.41; CI 1.12, 1.78) and a cancer center visit (OR 1.88; CI 1.28, 2.77) compared to privately insured Hispanic survivors, after adjusting for age, sex, household income, highest level of educational attainment, and Grade 3 or 4 chronic condition.^31^, ^61^ Hispanic survivors were more likely to report a cancer center visit (females: OR 1.5; CI 1.1, 2.0; males: OR 1.7; CI 1.2, 2.3) in comparison with non‐Hispanic White survivors, and Hispanic males were more likely to report cancer‐related medical visit (OR 1.3; CI 1.0, 1.8) compared to non‐Hispanic White male survivors, after adjusting for age, cancer diagnosis, health insurance, household income, and highest level of educational attainment.^31^, ^61^

#### 3.2.2 Moderate quality of evidence

A total of 13 studies (1 prospective, 12 retrospective) explored the effect of current age on potential disparities among CCS.^17^, ^20^, ^21^, ^23^, ^25^, ^27^, ^28^, ^29^, ^31^, ^32^, ^33^, ^35^, ^36^ Of the 13 studies, nine indicated that older age is associated with a disparity in survivorship care and general care engagement (moderate QOE) among CCS.^17^, ^20^, ^21^, ^28^, ^29^, ^31^, ^32^, ^35^, ^36^ For example, a prospective study reported that the odds of difficulty obtaining care increased 3% (OR 1.03; CI 1.00, 1.06) for every year of age at survey, after adjusting for sex, marital status, employment, age at diagnosis, cancer diagnosis, and radiation therapy exposure.^17^

Across five studies (one prospective, four retrospective), being unemployed was not systematically associated with a disparity in survivorship care engagement and there were mixed results for other care outcomes (moderate QOE) among CCS.^17^, ^21^, ^25^, ^28^, ^31^ Overall, three of the five studies found no association between employment and disparities in survivorship care engagement among CCS.^21^, ^25^, ^28^ However, a prospective study found a significant association, in which CSS who were currently employed experienced a decrease in the odds of difficulty obtaining care (OR 0.52; CI 0.36, 0.75) compared to CCS who were unemployed, after adjusting for sex, marital status, age at survey, age at diagnosis, cancer diagnosis, and radiation therapy exposure.^17^

#### 3.2.3 Low quality of evidence

Overall, 14 studies (1 prospective, 13 retrospective) evaluated potential disparities by cancer treatment (by type or intensity) among CCS.^17^, ^19^, ^21^, ^23^, ^25^, ^26^, ^27^, ^28^, ^30^, ^31^, ^32^, ^33^, ^36^, ^38^ Across these studies, there was evidence that survivors who received less intense treatment (e.g., guided by the Intensity of Treatment Rating Scale^70^) or single modality regimens were less likely to be engaged in survivorship care and general care^21^, ^23^, ^27^, ^28^, ^30^, ^31^, ^38^; however, some conflicting evidence was identified, in which four studies did not find an association with treatment type or intensity (low QOE).^17^, ^27^, ^33^, ^36^

In total, 14 studies (1 prospective, 13 retrospective) reported on potential disparities by biologic sex, six of which reported that male sex was associated with lower survivorship engagement (compared to female sex)^19^, ^21^, ^27^, ^29^, ^30^, ^33^; however, six studies showed no association^20^, ^23^, ^28^, ^31^, ^36^, ^37^, ^38^ or a conflicting direction of effects^17^ (low QOE). For example, a prospective study found that the odds of experiencing difficulty obtaining care in the prior year decreased for males (OR 0.59; CI 0.41, 0.85) compared to females, after adjusting for age at survey, marital status, employment status, age at diagnosis, cancer diagnosis, and radiation therapy exposure.^17^

A total of 13 studies (1 prospective, 12 retrospective) evaluated potential disparities by cancer type, grade, or stage at diagnosis, and relapse status among CCS.^17^, ^19^, ^22^, ^23^, ^26^, ^27^, ^28^, ^30^, ^31^, ^32^, ^33^, ^36^, ^38^ Across studies, no clear pattern for these disparities was found. In accordance with one prospective study, cancer diagnosis (e.g., hematologic malignancies compared to solid tumors) may be associated with disparities, but the exact pattern remains unclear (low QOE).^14^

Eleven studies (1 prospective, 10 retrospective) addressed potential disparities in survivorship care engagement associated with age at diagnosis among CCS.^17^, ^26^, ^28^, ^29^, ^30^, ^31^, ^32^, ^34^, ^38^ The evidence primarily indicated that older age at diagnosis is associated with a disparity both in survivorship care and general care engagement among CCS, as reported in 6 studies.^26^, ^28^, ^32^, ^34^, ^37^ However, some inconsistent results were found in six studies (low QOE).^17^, ^27^, ^30^, ^32^, ^37^, ^38^

Overall, seven studies (1 prospective, 6 retrospective) explored the effect of geographic area on potential disparities among CCS.^17^, ^19^, ^23^, ^27^, ^30^, ^31^, ^38^ Some evidence of an association between being from an underserved or rural area and experiencing a disparity in survivorship care engagement was reported in four studies^19^, ^23^, ^30^, ^31^; however, conflicting evidence where other three studies indicated no association or a positive association exists (low QOE).^17^, ^27^, ^38^

Only six retrospective studies assessed potential disparities in survivorship care engagement by insurance type (low QOE).^20^, ^23^, ^24^, ^29^, ^31^, ^36^ There was some indication of disparities by insurance type identified in all six studies, but there was no systematic association with disparities in survivorship care engagement among CCS. For example, two studies identified a benefit for CCS who were publicly insured^24^, ^31^; while, two other studies identified disparities for those who were publicly insured^23^, ^36^ and two remaining studies identified disparities for those who were publicly insured or uninsured (all in comparison with those who were privately insured).^20^, ^29^

Only three retrospective studies evaluated time since diagnosis. Of those, two studies indicated that CCS farther from diagnosis were less likely to receive survivorship care and had lower general care engagement (low QOE) than more recently diagnosed CCS.^27^, ^30^ However, two studies showed no association and conflicting results.^23^, ^27^

Only two retrospective studies assessed disparities associated with educational attainment.^28^, ^31^ One study indicated that lower levels of educational attainment are associated with a disparity in survivorship care engagement among CCS,^28^ while another study did not find significant results (low QOE).^30^

Six retrospective studies assessed potential disparities by income.^20^, ^25^, ^27^, ^30^, ^31^, ^38^ However, conflicting results were found, with three studies finding a positive association with income,^25^, ^30^, ^31^ one study finding less adherence to higher cost procedures,^20^ and three studies finding no association or conflicting results.^27^, ^31^, ^38^ Across the available research it, remained unclear whether income was associated with disparities in survivorship care engagement among CCS (very low QOE).

No included studies that assessed survivorship care disparities examined those by self‐identified gender (insufficient evidence).

## 4 DISCUSSION

In this systematic review, we identified strong evidence of disparities associated with race, ethnicity, and insurance status and moderate evidence for employment status and current age. The review documents the current state of the disparities literature for CCS and opportunities for interventions to reduce disparities at multiple levels, and areas for future investigation.

There was high QOE of survivorship care disparities among CCS despite contemporary policy changes designed to propel the health care system toward achieving health equity (e.g., the US Patient Protection and Affordable Care Act's Medicaid expansion).^71^, ^72^ Among CCS, disparities in survivorship care engagement are particularly impactful for CCS who are people of color or who are uninsured.^17^, ^19^, ^21^, ^22^, ^23^, ^24^, ^26^, ^29^, ^31^, ^34^, ^36^, ^38^ There was high QoE for race, ethnicity, and insurance status, indicating that CCS who were of non‐White race, Hispanic ethnicity, or uninsured are less likely to engage in survivorship care.^17^, ^21^, ^22^, ^23^, ^24^, ^26^, ^29^, ^31^, ^34^, ^36^, ^38^ However, it is worth noting that existing studies adjust for different covariates, hence it is difficult to estimate effect size. Nevertheless, these results support future studies to further “unpack” potential underlying factors. For instance, to counteract these disparities, providers could target survivors who may experience insurance instability or churn in coverage (e.g., those covered by Medicaid as children) to ensure survivors and their caregivers understand the importance of continuous engagement in survivorship care and potential resources for where they can seek survivorship services when they lack insurance coverage or experience instability in insurance. Additionally, if in‐network survivorship care is not an option locally for a survivor, a primary care provider could provide the needed survivorship care in some cases, as clinically appropriate, with a comprehensive survivorship care plan and close communication with a survivorship care provider.

This review explored a variety of disparities in suvivorship care for CCS, but in most cases, the existing research base remains limited and shows conflicting results, which hinders our ability to make concluding statements for specific survivorship care disparities. For instance, although a substantial amount of research examined cancer treatment, type of cancer diagnosis, sex, age at diagonosis, and geographic area, the QOE is low. Disparities by income have also been examined in a small number of studies; however, the QOE is very low as it is unclear whether income is associated with disparities across studies. Even fewer studies have examined time since diagnosis, insurance type, and educational attainment, contributing to a low QOE assessment. There was insufficient evidence of disparities by gender because we did not identify studies that reported on self‐identified gender (rather than biological sex). Taken together, these results underscore the gaps in the field that need to be addressed. Coordinated efforts to study survivorship care disparities among CCS have been implemented through the Childhood Cancer Star Act and other policy changes.^73^ Future investigations should use prospective, multilevel study designs across independent research groups and study cohorts to understand drivers of disparities and to inform strong evidence‐based conclusions.

The review has several strengths and limitations. We reviewed a large quantity of empirical literature to identify relevant studies for this review. Our broad search was designed to identify studies that have evaluated potential disparities, regardless of the result of the evaluation, in order to objectively document the presence and the absence of disparities. This comprehensive review is nonetheless limited by the small number of studies that have empirically assessed disparities among CCS, our reliance on the methodological rigor of identified studies to detect and report disparities, and the restriction to studies reported in English. Furthermore, the vast majority of included studies are quantitative in nature. Qualitative or mixed methods studies would allow for more context about the intersectionality of identifies that contribute to or experience disparities. Future research should specifically prioritize exploring potential disparities among CCS to improve our understanding of health inequities and to inform how the diversity of CCS should best be supported.

## 5 CONCLUSION

Across identified empirical research, we found high QOE that non‐White race or Hispanic ethnicity and lack of insurance are associated with disparities in survivorship care among CCS. Nonetheless, multiple other variables, such as employment, income, insurance type, education, cancer diagnosis, age at diagnosis, time since diagnosis, cancer treatment, geographic area, sex, and self‐identified gender, have been suggested in individual studies and reported effects warrant further investigation. Furthermore, CCS face many complex and multifactorial challenges, including those that interplay with social determinants of health, such as socioeconomic hardship (e.g., changes in income, increasing out‐of‐pocket medical expenses, rising debt), decreased educational attainment, detrimental employment factors (e.g., being unemployed, lack of benefits, limited or no paid time off and/or sick leave), inadequate insurance coverage (e.g., being under or uninsured, barriers due to type of insurance, transitions or changes in insurance coverage), and being from an underserved or minority group (e.g., racial or ethnic minorities, urban, rural, nonmetropolitan regions). These factors may lead to barriers to care, and exacerbate disparities, or, could independently act as disparate groups. Future prospective research is needed to fully understand the myriad of disparities faced by CCS, particularly using a multilevel approach, to understand how we may design studies to intervene to counteract these disparities.

## AUTHOR CONTRIBUTIONS

**Erin M. Mobley:** Conceptualization (equal); data curation (equal); formal analysis (equal); investigation (equal); methodology (equal); project administration (equal); supervision (equal); validation (equal); visualization (equal); writing – original draft (lead); writing – review and editing (lead). **Diana J. Moke:** Investigation (equal); writing – review and editing (equal). **Joel Milam:** Investigation (equal); writing – review and editing (equal). **Carol Y. Ochoa‐Dominguez:** Data curation (equal); formal analysis (equal); investigation (equal); validation (equal); writing – review and editing (equal). **Julia Stal:** Data curation (equal); formal analysis (equal); investigation (equal); validation (equal); writing – review and editing (equal). **Halle Mitchell:** Data curation (equal); formal analysis (equal); investigation (equal); validation (equal); writing – review and editing (equal). **Naghmeh Aminzadeh:** Formal analysis (equal); investigation (equal); validation (equal); writing – review and editing (equal). **Maria Bolshakova:** Data curation (equal); formal analysis (equal); investigation (equal); validation (equal); writing – review and editing (equal). **Raymond B. Mailhot Vega:** Investigation (equal); writing – review and editing (equal). **Jennifer Dinalo:** Data curation (equal); resources (equal); writing – review and editing (equal). **Aneesa Motala:** Data curation (equal); project administration (equal); resources (equal); software (equal); visualization (equal); writing – review and editing (equal). **Susanne Hempel:** Conceptualization (equal); data curation (equal); formal analysis (equal); funding acquisition (equal); investigation (equal); methodology (equal); project administration (equal); resources (equal); software (equal); supervision (equal); validation (equal); visualization (equal); writing – original draft (equal); writing – review and editing (equal).

## FUNDING INFORMATION

This manuscript builds on work supported by the Agency for Healthcare Research and Quality (AHRQ), U.S. Department of Health and Human Services (HHS) under Contract No. 75Q80120D00009/Task Order 75Q80120F32001. The authors of this document are responsible for its content, and the content does not necessarily represent the official views of or imply endorsement by AHRQ or HHS. EMM and CYO were supported by 5T32CA009492‐35, EMM is supported by 5R33AG056540 and 2L40CA253827, RBMV is supported by 3UL1TR001427‐07S1, and CYOD is supported by 4K00CA264294‐02.

## CONFLICT OF INTEREST STATEMENT

None of the authors have any affiliations or financial involvement that conflicts with the material presented in this manuscript.

## ETHICS STATEMENT

Determined to be exempt by the Institutional Review Board at the University of Southern California (HS‐20‐00483).

## ROLE OF THE FUNDER

The authors of this document are responsible for its content. The content does not necessarily represent the official views of or imply endorsement by the Agency for Healthcare Research and Quality, the Department of Health and Human Services, or any other funders.

## AUTHOR DISCLOSURE STATEMENT

The listed authors fulfill all authorship requirements as stated in the Recommendations for the Conduct, Reporting, Editing, and Publication of Scholarly work in Medical Journals and have no competing financial interests that exist.

## Supporting information


Appendix S1:

## Data Availability

The data are available in the Appendix.
