# Supplementary material for: Disparities in pediatric cancer survivorship care: A systematic review
Source: Cancer Med. 2023 Aug 8;12(17):18281–305. doi: 10.1002/cam4.6426 (PMC10524017; doi:10.1002/cam4.6426)
Supplement: Supplementary file 1 — Appendix S1: [file CAM4-12-18281-s001.docx]

**APPENDIX**

**Search Strategies and Sources**

This appendix summarizes the methods used for this systematic review. We searched a variety of sources and applied several measures to reduce potential reviewer errors and bias. This section describes the search strategies for published and unpublished studies. The search strategies for the individual databases were developed, executed, and documented by an experienced librarian and were peer-reviewed by an experienced methodologist. We searched the research databases PubMed, CINAHL, and PsycINFO using the specified terminology below for each guiding question. PubMed indexes biomedical literature, CINAHL includes nursing literature, and PsycINFO is a resource for behavioral and social science research.

**PubMed**

Date: 06/22/2022

Terms:

(“Neoplasms”[Mesh] OR “Medical Oncology”[Mesh] OR “Oncology Service, Hospital”[Mesh] OR “Oncology Nursing”[Mesh] OR “Cancer Care Facilities”[Mesh] OR “National Cancer Institute (U.S.)”[Mesh] OR “American Cancer Society”[Mesh] OR “antineoplastic*” OR “anti-neoplastic*” OR “anti neoplastic*” OR “oncolog*” OR “neoplasm” OR “neoplasms” OR “tumor” OR “tumors” OR “cancer” OR “cancers” OR “malignan*” OR “carcinoma” OR “carcinomas”)

AND

(“child”[MeSH] OR “adolescent”[MeSH] OR "Minors"[Mesh] OR "Pediatrics"[Mesh] OR "Pediatricians"[Mesh] OR "Hospitals, Pediatric"[Mesh] OR "Intensive Care Units, Pediatric"[Mesh] OR "Intensive Care, Neonatal"[Mesh] OR “neonat*“ OR “newborn“ OR “newborns“ OR “infan*“ OR “baby“ OR “babies“ OR “nursery“ OR “nurseries“ OR “toddler“ OR “toddlers“ OR “preschool*“ OR “pre school*” OR “child*“ OR “kid“ OR “kids“ OR “juvenile“ OR “juveniles“ OR “minor“ OR “minors“ OR “youth“ OR “youths“ OR “youngster“ OR “youngsters“ OR “girl“ OR “girls“ OR “boy“ OR “boys“ OR “elementary school*” OR “grade school*” OR “preadolescen*“ OR “pre adolescen*” OR “preteen*“ OR “pre teen*” OR “middle school*” OR “adolescen*“ OR “teen*“ OR “high school*” OR “pediatric*“ OR “PICU“ OR “NICU“ OR “young adult” OR “young adults”)

AND

(“Social Determinants of Health”[MeSH] OR “Health Status Disparities”[MeSH] OR “Sociology, Medical”[MeSH] OR “Healthcare Disparities”[MeSH] OR “Sociological Factors”[MeSH] OR “social determinants of health” OR “socioeconomic” OR “access to healthcare” OR “Barriers to healthcare” OR ((“Black” OR “African American” OR “Alaskan Native” OR “native American” OR “white” OR “Asian” OR “Native Hawaiian” OR “Pacific Islander” OR “Hispanic” OR “Hispanics” OR “Latino” OR “Latina” OR “LatinX” OR “Latinos” OR “Latinas” OR “Blacks” OR “African Americans” OR “Alaskan Natives” OR “native Americans” OR “whites” OR “Asians” OR “Native Hawaiians” OR “Pacific Islanders” OR “health*” OR “medic*” OR “insurance” OR “insurances” OR “education*”) AND (“inequit*” OR “disparit*” OR “inequal*”)) OR ((“social*” OR “sociolog*” OR “sociology*”) AND (“factor” OR “factors” OR “trait” OR “traits” OR “attribute” OR “attributes” OR “characteristic” OR “characteristics” OR “phenomen*”)))

AND

(“Survivors”[Mesh] OR “Survivorship”[Mesh] OR "Population Surveillance"[Mesh] OR "Aftercare"[Mesh] OR ”Survivor” OR ”survivors” OR ”survivorship” OR “surveillance” OR “aftercare” OR “post-treatment” OR “post treatment” OR “post-treatments” OR “post treatment” OR “follow up care” OR “follow-up care” OR “Long term follow up” OR “long-term follow-up”)

**CINAHL**

Date: 06/22/2022

Terms:

(MH "Neoplasms+" OR MH "Oncology+" OR MH "Oncology Care Units" OR MH "Oncologic Nursing+" OR MH "Oncologic Care+" OR MH “Cancer Care Facilities” OR MH "National Cancer Institute (U.S.)" OR MH "American Cancer Society" OR “antineoplastic*” OR “anti-neoplastic*” OR “anti neoplastic*” OR “oncolog*” OR “neoplasm” OR “neoplasms” OR “tumor” OR “tumors” OR “cancer” OR “cancers” OR “malignan*” OR “carcinoma” OR “carcinomas”)

AND

(MH "Child+" OR MH "Minors (Legal)" OR MH "Adolescence+"OR MH "Pediatrics+" OR MH "Hospitals, Pediatric" OR MH "Intensive Care Units, Pediatric+" OR MH "Pediatric Units+" OR MH "Pediatricians" OR MH "Pediatric Nursing+” OR MH "Neonatal Intensive Care Nursing" OR MH "Intensive Care Units, Neonatal" OR MH "Intensive Care, Neonatal+" OR “neonat*“ OR “newborn“ OR “newborns“ OR “infan*“ OR “baby“ OR “babies“ OR “nursery“ OR “nurseries“ OR “toddler“ OR “toddlers“ OR “preschool*“ OR “pre school*” OR “child*“ OR “kid“ OR “kids“ OR “juvenile“ OR “juveniles“ OR “minor“ OR “minors“ OR “youth“ OR “youths“ OR “youngster“ OR “youngsters“ OR “girl“ OR “girls“ OR “boy“ OR “boys“ OR “elementary school*” OR “grade school*” OR “preadolescen*“ OR “pre adolescen*” OR “preteen*“ OR “pre teen*” OR “middle school*” OR “adolescen*“ OR “teen*“ OR “high school*” OR “pediatric*“ OR “PICU“ OR “NICU“ OR “young adult” OR “young adults”)

AND

(MH "Social Determinants of Health" OR MH "Health Status Disparities" OR MH "Healthcare Disparities" OR “social determinants of health” OR “socioeconomic” OR “access to healthcare” OR “Barriers to healthcare” OR ((“Black” OR “African American” OR “Alaskan Native” OR “native American” OR “white” OR “Asian” OR “Native Hawaiian” OR “Pacific Islander” OR “Hispanic” OR “Hispanics” OR “Latino” OR “Latina” OR “LatinX” OR “Latinos” OR “Latinas” OR “Blacks” OR “African Americans” OR “Alaskan Natives” OR “native Americans” OR “whites” OR “Asians” OR “Native Hawaiians” OR “Pacific Islanders” OR “health*” OR “medic*” OR “insurance” OR “insurances” OR “education*”) AND (“inequit*” OR “disparit*” OR “inequal*”)) OR ((“social*” OR “sociolog*” OR “sociology*”) AND (“factor” OR “factors” OR “trait” OR “traits” OR “attribute” OR “attributes” OR “characteristic” OR “characteristics” OR “phenomen*”)))

AND

(MH “Survivors” OR MH “Survivorship” OR MH "Population Surveillance" OR MH "After care" OR ”Survivor” OR ”survivors” OR ”survivorship” OR “surveillance” OR “aftercare” OR “post-treatment” OR “post treatment” OR “post-treatments” OR “post treatment” OR “follow up care” OR “follow-up care” OR “Long term follow up” OR “long-term follow-up”)

**PsycINFO**

Date: 06/22/2022

Terms:

(MAINSUBJECT.EXACT.EXPLODE("Neoplasms") OR MAINSUBJECT.EXACT.EXPLODE("Oncology") OR “antineoplastic*” OR “anti-neoplastic*” OR “anti neoplastic*” OR “oncolog*” OR “neoplasm” OR “neoplasms” OR “tumor” OR “tumors” OR “cancer” OR “cancers” OR “malignan*” OR “carcinoma” OR “carcinomas”)

AND

(MAINSUBJECT.EXACT("Adolescent Development") OR MAINSUBJECT.EXACT.EXPLODE("Pediatrics") OR MAINSUBJECT.EXACT.EXPLODE("Pediatricians") OR MAINSUBJECT.EXACT.EXPLODE("Neonatal Intensive Care") OR “neonat*“ OR “newborn“ OR “newborns“ OR “infan*“ OR “baby“ OR “babies“ OR “nursery“ OR “nurseries“ OR “toddler“ OR “toddlers“ OR “preschool*“ OR “pre school*” OR “child*“ OR “kid“ OR “kids“ OR “juvenile“ OR “juveniles“ OR “minor“ OR “minors“ OR “youth“ OR “youths“ OR “youngster“ OR “youngsters“ OR “girl“ OR “girls“ OR “boy“ OR “boys“ OR “elementary school*” OR “grade school*” OR “preadolescen*“ OR “pre adolescen*” OR “preteen*“ OR “pre teen*” OR “middle school*” OR “adolescen*“ OR “teen*“ OR “high school*” OR “pediatric*“ OR “PICU“ OR “NICU“ OR “young adult” OR “young adults”)

AND

(MAINSUBJECT.EXACT("Social Discrimination") OR MAINSUBJECT.EXACT.EXPLODE("Health Disparities") OR “social determinants of health” OR “socioeconomic” OR “access to healthcare” OR “Barriers to healthcare” OR ((“Black” OR “African American” OR “Alaskan Native” OR “native American” OR “white” OR “Asian” OR “Native Hawaiian” OR “Pacific Islander” OR “Hispanic” OR “Hispanics” OR “Latino” OR “Latina” OR “LatinX” OR “Latinos” OR “Latinas” OR “Blacks” OR “African Americans” OR “Alaskan Natives” OR “native Americans” OR “whites” OR “Asians” OR “Native Hawaiians” OR “Pacific Islanders” OR “health*” OR “medic*” OR “insurance” OR “insurances” OR “education*”) AND (“inequit*” OR “disparit*” OR “inequal*”)) OR ((“social*” OR “sociolog*” OR “sociology*”) AND (“factor” OR “factors” OR “trait” OR “traits” OR “attribute” OR “attributes” OR “characteristic” OR “characteristics” OR “phenomen*”)))

AND

(MAINSUBJECT.EXACT.EXPLODE("Survivors") OR MAINSUBJECT.EXACT.EXPLODE("Aftercare") OR ”Survivor” OR ”survivors” OR ”survivorship” OR “surveillance” OR “aftercare” OR “post-treatment” OR “post treatment” OR “post-treatments” OR “post treatment” OR “follow up care” OR “follow-up care” OR “Long term follow up” OR “long-term follow-up”)

**Inclusion and exclusion of studies**

After completing the searches described above, we used the criteria specified in the table below to determine whether studies would be included or excluded.

**Table A-1. Criteria for inclusion and exclusion of studies**

| PICOTSS | Inclusion | Exclusion |
| --- | --- | --- |
| Population | Childhood cancer survivors (CCS) of all ages. We accepted the authors’ definition of CCS. Mixed samples were eligible where studies include at least 50% CCS or report a subgroup analysis. In studies not self-identifying as CCS research, we applied the following criteria: diagnosed before age 21, received primary acute treatment for any cancer, currently in remission, and currently receiving or eligible to receive survivorship care services, care plans, and/or models of follow-up care. | Studies that predominantly included populations other than CCS, that included patients diagnosed predominantly after the age of 20, that had other conditions than cancer, or that were currently undergoing treatment for cancer. |
| Independent variables and interventions | Survivorship care. We included studies addressing healthcare approaches aimed at the health and wellbeing of cancer survivors. | Studies without reference to survivorship care and studies not addressing care disparities. |
| Comparators | We accepted the authors’ choice of a participant characteristic comparator. Studies could compare subgroups to the general population of CCS or compare multiple participant subgroups defined by participant characteristics (e.g., race/ethnicity, socioeconomic status, gender, rural residence, educational attainment or patient or their parents, other populations that experience health disparities). | Studies not addressing patient or intervention characteristics. |
| Outcomes | Disparities in any patient outcomes related to utilization of survivorship care services, care plans, or models of care; intermediate health outcomes and adverse events; mortality; late effects and morbidity (including psychosocial); quality of life, wellbeing, and satisfaction with care; and cost and resource utilization. | Studies that did not address disparities to survivorship care for pediatric survivors. |
| Timing | No timing restriction applied. Studies may have addressed CCS who recently or long in the past experienced pediatric cancer now in remission. | No exclusions applied. |
| Setting(s) | All care settings applicable to US settings were eligible, including primary, secondary, and tertiary care; inpatient and outpatient care; pediatric and adult care context. | Studies in resource-limited settings such as developing countries were reviewed for comparability with US settings. |
| Study design and other limiters | English-language publications.  Primary studies reporting empirical data (including both quantitative and qualitative data).  Studies may either report on distinct subgroups (e.g., dividing the sample by geographic characteristic and reporting data separately for rural and for urban participants) or studies may report associations with participant characteristics (e.g., reporting correlations with a factor of interest such as differences by sex). | Evaluations reported only in abbreviated format (e.g., in a conference abstract) with the exception of trial records.  Studies exclusively reported in non-English publications.  Systematic reviews were retained for reference mining but are not eligible for inclusion. |

CCS, Childhood Cancer Survivor

**Table A-2. Evidence table**

| **Author, Year**  **Related Publications**  ***Study Name/Trial ID***  **Study size**  **Analysis type** | **Country** | **Proportion of CCS**  **Cancer origin** | **Disparities analyzed and findings** | **Assessed outcome** |
| --- | --- | --- | --- | --- |
| **Baedke, 2022^1^**  Hudson, 2013^2^; Howell, 2021^3^  *St. Jude LIFE*  Study size: 3310^1^; 1713^2^; 13087^3^  Analysis type: Survey, analytic study | USA | Proportion of CCS: 100%  Multiple cancer origin | **Race/ethnicity:** In multivariable analysis and in comparison to privately insured Whites, privately insured Blacks (OR 1.43; CI 1.01, 2.01), uninsured Blacks (OR 4.41; CI 2.93, 6.64) and uninsured Whites (OR 3.53; CI 2.75, 4.55) were more likely to forgo needed care, and publicly insured Whites (OR 0.69; CI 0.53, 0.89) were less likely to forgo needed care, after adjusting for current age, sex, treatment era, cancer diagnosis, treatment received, major surgery, self-reported health status, income, and education.^1^  Not significant: In multivariable analysis and in comparison to privately insured Whites, publicly insured Blacks and uninsured, privately insured, or publicly insured Hispanic/Latinxs were not significantly associated with forgoing care, after adjusting for current age, sex, treatment era, cancer diagnosis, treatment received, major surgery, self-reported health status, income, and education.^1^ | **Health services/ economics:**  Primary care, specialty care, or other care utilization (forgone care due to finances)  Financial hardship, costs, and resource utilization |
| **Barakat, 2012^4^**  Study size: 173^4^  Analysis type: Analytic study | USA | Proportion of CCS: 100%  Multiple cancer origin | **Race/ethnicity:** In univariable analysis, patients of non-white race were more likely to have no shows for survivorship visits (OR 0.27, p=0.001).^4^  **Underserved or rural:** In univariable analysis, patients who lived farther than 57.6 km to the hospital were less likely (OR 0.24, p=0.003) to attend a follow-up or survivorship visit as patients who lived closer.^4^  **Sex:** In univariable analysis, females were twice as likely as males to attend a survivorship clinic visit 5 years post-diagnosis (OR 2.20, p=0.077).^4^  **Treatment:** Patients who were off treatment for a longer period of time were about half as likely (OR 0.44, p=0.004) to attend a follow-up or survivorship visit in comparison to those who more recently completed treatment.^4^  **Cancer diagnosis:** In univariate analysis, those diagnosed with brain tumors (OR 0.24, p=0.055) were less likely to attend a survivorship visit compared to patients with leukemia or lymphoma.^4^ | **Survivorship care:** Utilization of survivorship care services, care plans, or models of care (survivorship visits, no shows to visits) |
| **Benedict, 2021^5^**  Study size: 286  Analysis type: Analytic study | USA | Proportion of CCS: 100%  Multiple cancer origin | **Race/ethnicity:** In multivariable analysis, patients who identified as black (OR 0.47; CI 0.23, 0.90) were associated with greater likelihood of nonadherence, after adjusting for insurance type, age, sex, and cost of recommended procedures.^5^  **Income:** In multivariable analysis, patients with recommended procedures that exceeded a median cost of $400 were less likely to be adherent than those with recommended procedures that cost less than $400 (OR 0.32; CI 0.22, 0.46), after adjusting for race, insurance type, age, and sex.^5^  **Sex:** Not significant: In multivariable analysis, male sex was not significantly related to nonadherence, after adjusting for race, age, insurance type, and cost of recommended procedures.^5^  **Insurance:** In multivariable analysis, being insured by Medicaid or uninsured (OR 0.59; CI 0.36, 0.96) was related to greater likelihood of nonadherence, after adjusting for race, age, sex, and cost of recommended procedures.^5^  **Age at study or enrollment, current age:** In multivariable analysis, older age (OR 0.97; CI 0.94, 1.0) was related to greater likelihood of nonadherence, after adjusting for race, insurance type, sex, and cost of recommended procedures.^5^ | **Survivorship care:** Utilization of survivorship care services, care plans, or models of care (adherence to survivorship screening procedures)  **Health services/ economics:**  Financial hardship, costs, and resource utilization |
| **Berg, 2016^6^**  Study size: 106^6^  Analysis type: Survey | USA | Proportion of CCS: 100%  Multiple cancer origin | **Sex:** In the multivariate regression, male sex (OR 0.09; CI 0.03, 0.35) was associated with less than annual healthcare provider visits, after adjusting for current age, insurance coverage, and chemotherapy exposure.^6^  **Employment**: Not significant: Employment status was not significantly associated with annual healthcare provider visits, after adjusting for current age, insurance coverage, and chemotherapy exposure.^6^  **Insurance:** In multivariable analysis, lack of health insurance was associated with less than annual healthcare provider visits (OR 0.04; CI 0.01, 0.33), after adjusting for current age, sex, and chemotherapy exposure.^6^  **Treatment:** In the multivariable model, exposure to chemotherapy was associated with less than annual healthcare provider visits (OR 5.73; CI 0.98, 40.30), after adjusting for current age, sex, and insurance coverage.^6^  **Age at diagnosis year of diagnosis, or time since diagnosis:**  **Age at study or enrollment, current age:** In the multivariable model, older current age was associated with less than annual healthcare provider visits (OR 1.35; CI 1.11-1.63), after adjusting for chemotherapy exposure, sex, and insurance coverage.^6^ | **Survivorship care:** Utilization of survivorship care services, care plans, or models of care |
| **Berkman, 2019^7^**  Study size: 198^7^  Analysis type: Analytic study | USA | Proportion of CCS: 100%  Brain/CNS | **Race/ethnicity:** In the multivariable model, African American race was a significant predictor of missed visits (+6.90%, p<0.001) when adjusting for tumor grade and insurance status.^7^  **Insurance:** In multivariable analysis, participants with self-pay status or uninsured were significantly more likely to have missed visits (+8.46%, p=0.005) when adjusting for tumor grade and race.^7^  **Cancer diagnosis:** Not significant: Tumor grade was not significantly associated with missed visits, when adjusting for race and insurance.^7^ | **Survivorship care:** Utilization of survivorship care services, care plans, or models of care  **Health services/ economics:**  Primary care, specialty care, or other care utilization (missed visits, time from symptom onset to presentation for visit) |
| **Crom, 2007^8^**  Klosky, 2008^9^; Hudson, 2002^10^  *After Completion of Therapy (ACT) Clinic*  Study size: 1437 survivors^8^; 941 survivors^9^; 251 survivors^10^  Analysis type: Analytic study | USA | Proportion of CCS: 100%  Multiple cancer origin | **Race/ethnicity:** Non-whites were nearly two times more likely to be non-attenders at survivorship clinic (OR 1.88; 1.19, 2.97) compared to whites, after adjusting for age, socioeconomic status, race, years from diagnosis, additional cancer event, insurance status, means of travel, distance from hospital, scheduled social work consultation, and type of clinic visit.^8,9^  **Underserved or rural:** Those who traveled by a car were more likely to be non-attenders at survivorship clinic (OR 12.74; 3.97, 40.86) compared to those who traveled by bus, after adjusting for age, socioeconomic status, race, years from diagnosis, additional cancer event, insurance coverage, distance from hospital, scheduled social work consultation, and type of clinic visit.^8,9^  Not significant: Those who traveled a specific distance from hospital did not experience a significant association with non-attendance at survivorship clinic, after adjusting for age, socioeconomic status, race, years from diagnosis, additional cancer event, insurance coverage, distance from hospital, scheduled social work consultation, and type of clinic visit.^8,9^  **Sex:** The odds of difficulty obtaining care decreased for males in the prior year (OR 0.59; CI 0.41, 0.85) compared to females, after adjusting for age at survey completion, marriage, employment, age at diagnosis, cancer diagnosis, and radiation therapy.^8^  **Employment**: Those who were currently employed experienced a decrease in the odds of difficulty obtaining care (OR 0.52; CI 0.36, 0.75) compared to those who were unemployed, after adjusting for sex, age at survey, marriage, age at diagnosis, cancer diagnosis, and radiation therapy.^8^  **Insurance:** Those who were currently insured experienced a decrease in the odds of difficulty obtaining care (OR 0.18; CI 0.12, 0.26) compared to those who were uninsured, after adjusting for sex, age at survey, marriage, employment status, age at diagnosis, cancer diagnosis, and radiation therapy.^8^  Those who were privately insured were more likely to be non-attenders at survivorship clinic (OR 2.36; CI 1.98, 3.79) compared to those who were uninsured, after adjusting for age, socioeconomic status, race, years from diagnosis, additional cancer event, means of travel, distance from hospital, scheduled social work consultation, and type of clinic visit.^8,9^  Not significant: Those who were publicly insured did not experience a significant association with non-attendance at survivorship clinic, after adjusting for age, socioeconomic status, race, years from diagnosis, additional cancer event, means of travel, distance from hospital, scheduled social work consultation, and type of clinic visit.^8,9^  **Treatment:** Not significant: Treatment with radiation did not have a significant association with difficulty obtaining care, after adjusting for sex, age at survey, marriage, employment status, insurance coverage, cancer diagnosis, and age at diagnosis.^8^  **Age at diagnosis year of diagnosis, or time since diagnosis:** Not significant: Age at diagnosis did not have a significant association with difficulty obtaining care, after adjusting for sex, age at survey, marriage, employment status, insurance coverage, cancer diagnosis, and radiation therapy.^8^  Year of diagnosis was not significantly associated with non-attendance at survivorship clinic, after adjusting for age, socioeconomic status, race, years from diagnosis, additional cancer event, insurance coverage, travel distance from hospital, scheduled social work consultation, and type of clinic visit.^8,9^  **Age at study or enrollment, current age:** The odds of difficulty obtaining care increased 3% (OR 1.03, 1.00, 1.06) for every year of age at survey, after adjusting for sex, marriage, employment, age at diagnosis, cancer diagnosis, and radiation therapy.^8^  **Cancer diagnosis:** Those who were diagnosed with a solid tumor experienced a increase in the odds of difficulty obtaining care (OR 1.84, 1.25, 2.71) compared to those who were diagnosed with a hematologic malignancy, after adjusting for sex, age at survey, marriage, employment status, insurance coverage, age at diagnosis, and radiation therapy.^8^  Not significant: Those who were diagnosed with a CNS tumor did not have a significant association with difficulty obtaining care compared to those who were diagnosed with a hematologic malignancy, after adjusting for sex, age at survey, marriage, employment status, insurance coverage, age at diagnosis, and radiation therapy.^8^  Those who experienced a cancer event did not have a significant association with non-attendance at survivorship clinic, after adjusting for age, socioeconomic status, race, years from diagnosis, insurance coverage, means of travel, distance from hospital, scheduled social work consultation, and type of clinic visit.^8,9^  **Other:** Those who were currently married experienced a decrease in the odds of difficulty obtaining care (OR 0.63, 0.42, 0.92), after adjusting for sex, age at survey, employment, age at diagnosis, cancer diagnosis, and radiation therapy.^8^  Not significant: Scheduled social work consultation and type of clinic visit were not significantly associated with non-attendance at survivorship clinic, after adjusting for age, socioeconomic status, race, years from diagnosis, additional cancer event, insurance coverage, travel distance from hospital, and year of diagnosis.^8,9^ | **Survivorship care:** Utilization of survivorship care services, care plans, or models of care (attenders versus non-attenders at survivorship visits, knowledge of late effects)  **Health services/ economics:**  Primary care, specialty care, or other care utilization: (difficulty obtaining care)  Financial hardship, costs, and resource utilization |
| **Daly, 2019^11^**  Study size: 866^11^  Analysis type: Analytic study | USA | Proportion of CCS: 100%  Multiple cancer origin | **Race/ethnicity:** As compared to non-Hispanic white survivors, those of non-Hispanic black (HR 0.64; 0.52, 0.79) or other races (HR 0.70; CI 0.49, 0.99) were less likely to have an initial survivorhip visit, when adjusting for sex, therapeutic modalities, current age, insurance status, and distance from the clinic.^11^  Not significant: There was not a significant relationship for those of Hispanic ethnicity in predicting an initial survivorship visit, after adjusting for sex, therapeutic modalities, current age, insurance status, and distance from the clinic.^11^  **Underserved or rural:** Those who lived 25-50 miles from the clinic (HR 0.76; CI 0.63, 0.93) or those who lived more than 50 miles from the clinic (HR 0.67; CI 0.54, 0.82) were significantly less likely to have an initial survivor clinic visit compared to those who lived less than 25 miles from the clinic, after adjusting for sex, race/ethnicity, therapeutic modalities, current age, and insurance status.^11^  **Sex:** Not significant: There was not a significant relationship by sex in predicting an initial survivorship visit, after adjusting for race/ethnicity, therapeutic modalities, current age, insurance status, and distance from the clinic.^11^  **Insurance:** Those who had Medicaid at diagnosis (HR 0.77, 0.64-0.92) were less likely than those who had private insurance to have had an initial survivor visit, after adjusting for sex, race/ethnicity, therapeutic modalities, current age, and distance from the clinic.^11^  Not significant: There was not a significant relationship for who were uninsured in predicting an initial survivorship visit, after adjusting for sex, race/ethnicity, therapeutic modalities, current age, and distance from the clinic, in comparison to those who were privately insured.^11^  **Treatment:** Those who received surgery only (HR 0.04, 0.02-0.08) or radiation only (0.24, 0.15-0.39) were less likely to have an initial survivor clinic visit compared to those who received chemotherapy only, after adjusting for sex, race/ethnicity, therapeutic modalities, current age, insurance status, and distance from the clinic.^11^  Not significant: There was not a significant relationship for those who received chemotherapy and radiation in predicting an initial survivorship visit, after adjusting for sex, race/ethnicity, therapeutic modalities, current age, insurance status, and distance from the clinic, in comparison to those who received chemotherapy only.^11^  **Age at diagnosis year of diagnosis, or time since diagnosis:** Not significant: Year of diagnosis was not significantly associated with an initial survivorship visit, after adjusting for sex, race/ethnicity, therapeutic modalities, insurance status, and distance from the clinic.^11^  **Age at study or enrollment, current age:** Those who were ages 6-11 (HR 1.55, 1.24-1.93) or 12-17 (HR 1.44, 1.14-1.83) were significantly more likely to have an initial survivor clinic visit compared to those who were between the ages of 2-5, after adjusting for sex, race/ethnicity, therapeutic modalities, insurance status, and distance from the clinic.^11^  Not significant: There was not a significant relationship for those age 18 or older in predicting an initial survivorship visit, after adjusting for sex, race/ethnicity, therapeutic modalities, insurance status, and distance from the clinic, in comparison to those who were between the ages of 2-5.^11^  **Cancer diagnosis:** Not significant: Type of cancer diagnosis and an additional cancer event (e.g., any recorded relapse, progression, or subsequent malignancy following initial cancer diagnosis) were not significantly associated with an initial survivorship visit, after adjusting for sex, race/ethnicity, therapeutic modalities, insurance status, distance from the clinic, and current age.^11^ | **Survivorship care:** Utilization of survivorship care services, care plans, or models of care (survivorship clinic attendence) |
| **Gardner, 2014^12^**  Study size: 36^12^  Analysis type: Survey | USA | Proportion of CCS: 100%  Multiple cancer origin | **Insurance:** In univariable analysis, use of a mental health professional was less likely to be associated with private insurance coverage, in comparison to those with public insurance or uninsured (chi-square: -0.34, p = 0.040).^12^ | **Psychosocial:** Psychological  **Health services/ economics:**  Primary care, specialty care, or other care utilization (psychosocial support services use) |
| **Johnson, 2004^13^**  Study size: 385^13^  Analysis type: Analytic study | UK | Proportion of CCS: 100%  Multiple cancer origin | **Income:** In the multivariable analysis, those in the least affluent socioeconomic status group were less likely to attend survivorship clinic in comparison to those in the most affluent group (OR 0.31, p=0.009), when adjusting for age, time since end of treatment, and employment status.^13^  **Employment**: Not significant: In the multivariable analysis, employment status was not significantly associated with attending survivorship visits in the multivariable model adjusting for current age, time since treatment completion, and socioeconomic status.^13^  **Treatment:** Not significant: In the multivariable analysis, time since treatment completion was not significantly associated with attending survivorship visits, when adjusting for current age, employment, and socioeconomic status.^13^  **Age at study or enrollment, current age:** Not significant: In the multivariable analysis, current age was not significantly associated with attending survivorship visits, when adjusting for current age, employment, and socioeconomic status.^13^ | **Survivorship care:** Utilization of survivorship care services, care plans, or models of care (survivorship visits) |
| **May, 2017^14^**  Study size: 442^14^  Analysis type: Analytic study | USA | Proportion of CCS: 100%  Multiple cancer origin | **Race/ethnicity:** In multivariable analyses and in comparison to white participants, individuals who were black (OR 2.0, 0.9-4.4) or of other races (OR 2.0, 0.4-9.2) were more likely to be lost to follow-up for more than 1000 days and not attend survivorship clinic, where Hispanic individuals (OR 0.7, 0.4-1.1) were less likely to be lost to follow-up for more than 1,000 days and not attend survivorship clinic, when adjusting for diagnosis, treatment exposure, stem cell transplant exposure, age group at diagnosis, and insurance status (global overall p-value=0.03).^14^  **Insurance:** In multivariable analyses and in comparison to those with insurance, those who were uninsured (OR 3.4, 1.2-9.2) were more likely to be lost to follow-up for more than 1,000 days and not attend survivorship clinic, when adjusting for diagnosis, treatment exposure, stem cell transplant exposure, age group at diagnosis, and race (global overall p-value=0.02).^14^  **Treatment:** In multivariable analyses, treatment with surgery alone (OR 6.70; CI 3.10, 14.90; in comparison to chemotherapy alone) and prior stem cell transplantation (OR 2.0; CI 1.04, 3.70; in comparison to no stem cell transplant) were more likely to be lost to follow-up for more than 1,000 days and not attend survivorship clinic, when adjusting for diagnosis, race, insurance, and age at diagnosis (treatment global overall p-value<0.001; stem cell transplant global overall p-value=0.04).^14^  **Age at diagnosis year of diagnosis, or time since diagnosis:** In multivariable analyses, older age at diagnosis (ages 5 to 9 OR 1.8; CI 1.1, 3.0; ages 10 to 14 OR 3.3; CI 1.8, 6.1; and ages 15 and above: OR 4.8; CI 2.1, 11.7; in comparison to those ages 0-4) was more likely to be lost to follow-up for more than 1,000 days and not attend survivorship clinic, when adjusting for diagnosis, race, insurance, treatment exposure, and stem cell transplant exposure (global overall p-value<0.001).^14^  **Cancer diagnosis:** Not significant: Cancer diagnosis (leukemia, lymphoma, CNS tumor, solid tumor) was not significantly associated with lack of survivorship care in the multivariable model, which controlled for treatment (chemotherapy, radiation, surgery, chemotherapy/radiation, chemotherapy/surgery, radiation/surgery, chemotherapy/radiation/surgery), stem cell transplant, race/ethnicity (white, black, Hispanic, other), and lack of insurance at last visit, which were all significant.^14^ | **Survivorship care:** Utilization of survivorship care services, care plans, or models of care (lost to follow-up for survivorship care) |
| **McBride, 2011^15^**  *Childhood, Adolescent, and Young Adult Cancer Survivors (CAYACS) Research Program*  Study size: 1157^15^  Analysis type: Analytic study | Canada | Proportion of CCS: 100%  Multiple cancer origin | **Income:** Not significant: Socioeconomic status was not significantly associated with physician visits to >/= 10 primary care visits, any specialist visits, or any oncologist visits, after adjusting for sex, rurality, current age, time since diagnosis, cancer diagnosis, age at diagnosis, relapse status, second cancer status, and type(s) of treatment received.^15^  **Underserved or rural:** In multivariable analysis, survivors from a small community (in comparison to a metropolitan designated area) were more likely to have a visit with an oncologist (RR=1.45; CI 1.0, 2.0; controlling for sex, socioeconomic status, sex, current age, time since diagnosis, diagnosis, age at diagnosis, relapse status, second malignancy, and treatment).^15^  Not significant: Residence in a metropolitan, large community, small community, or rural community was not significantly associated with physician visits to >/= 10 primary care visits or any specialist visits, after adjusting for sex, socioeconomic status, current age, time since diagnosis, cancer diagnosis, age at diagnosis, relapse status, second cancer status, and type(s) of treatment received.^15^  **Sex:** In multivariable analysis, female survivors were more likely to have 10 or more GP visits (RR 1.78; CI 1.5, 2.1), more likely to visit specialists (RR 1.15; CI 1.0, 1.3), and more likely to have seen an oncologist (RR 1.40; CI 1.1, 1.7) than male survivor  **Treatment:** In multivariable analysis, survivors who received any chemotherapy (RR 1.85; CI 1.0, 3.3), chemotherapy/surgery (RR 2.30; CI 1.3, 4.1), chemotherapy/radiation (RR=2.43, CI 1.3 to 4.0), radiation/surgery (RR 3.5; CI 2.0, 6.0), or chemotherapy/radiation/surgery (RR 3.95; CI 2.2, 7.1) were significantly more likely to have an oncologist visit compared with survivors who received only surgery (adjusting for sex, socioeconomic status, residence in a metropolitan/large community/small community/rural area, current age, time since diagnosis, diagnosis, age at diagnosis, relapse status, and second malignancy).^15^  Not significant: In multivariable analysis, type of treatment received was not significantly associated with >/= 10 primary care provider visits or specialist visits, when adjusting for sex, socioeconomic status, residence in a metropolitan/large community/small community/rural area, time since diagnosis, current age, age at diagnosis, cancer diagnosis, relapse status, and second malignancy.^15^  **Age at diagnosis year of diagnosis, or time since diagnosis:** In multivariable analysis, survivors who were 10-14 years since diagnosis (RR 0.53; CI 0.3, 0.9) and those ≥ 25 years since diagnosis (RR 0.31; CI 0.2, 0.6) were less likely to have a visit with an oncologist than those diagnosed 5-9 years prior were (controlling for socioeconomic status, residence in a metropolitan/large community/small community/rural area, current age, diagnosis, age at diagnosis, relapse status, second malignancy, and treatment).^15^  Not significant: Time since diagnosis (measured in years) was not significantly associated with >/= 10 visits with a primary care provider or a visit with a specialist, after adjusting for sex, socioeconomic status, residence in a metropolitan/large community/small community/rural area, current age, diagnosis, age at diagnosis, relapse status, second malignancy, and treatment. Age at diagnosis was not associated with >/= 10 primary care provider visits, specialist visits, or oncologists visits, when adjusting for sex, socioeconomic status, residence in a metropolitan/large community/small community/rural area, time since diagnosis, current age, age at diagnosis, cancer diagnosis, relapse status, second cancer diagnosis, and treatment.^15^  **Age at study or enrollment, current age:** In multivariable analysis, survivors ages 20-34 (RR 1.40; CI 1.2, 1.7) and those ≥ 35 years of age (RR 1.40; CI 1.1, 1.8) were more likely to have 10 or more GP visits than younger survivors ages 5-19 were, with a significant trend with increasing age (p=.003). In multivariable analysis, survivors ages 20-34 (RR 4.57; CI 3.3, 6.1) and those ≥ 35 years of age (RR 3.01; CI 1.9, 4.6) were more likely to have a visit with an oncologist than younger survivors ages 5-19 were, with a significant trend with increasing age (p<.001). Both models controlled for sex, socioeconomic status, residence in a metropolitan/large community/small community/rural area, time since diagnosis, diagnosis, age at diagnosis, relapse status, second malignancy, and treatment.^15^  Not significant: In multivariable analysis predicting a visit with a specialist, a non-significant association with age in 2000 was demonstrated, after controlling for sex, socioeconomic status, residence in a metropolitan/large community/small community/rural area, time since diagnosis, diagnosis, age at diagnosis, relapse status, second malignancy, and treatment.^15^  **Cancer diagnosis:** In multivariable analysis, survivors of bone tumors (RR 3.89; CI 1.9, 7.8) and those who had relapsed (RR 2.11; CI 1.4, 3.3) had an increased likelihood of oncologist visits compared with survivors of acute lymphoblastic leukemia; controlled for sex, socioeconomic status, residence in a metropolitan/large community/small community/rural area, time since diagnosis, current age, age at diagnosis, relapse status, second malignancy, and treatment).^15^  Not significant: Cancer diagnosis was not associated with >/= 10 primary care provider visits or specialist visits, when adjusting for sex, socioeconomic status, residence in a metropolitan/large community/small community/rural area, time since diagnosis, current age, age at diagnosis, relapse status, second malignancy, and treatment. Second malignancy was not associated with >/= 10 primary care provider visits, specialist visits, or oncologists visits, when adjusting for sex, socioeconomic status, residence in a metropolitan/large community/small community/rural area, time since diagnosis, current age, age at diagnosis, cancer diagnosis, relapse status, and treatment.^15^ | **Survivorship care:** Utilization of survivorship care services, care plans, or models of care (visit with an oncologist)  **Health services/ economics:**  Primary care, specialty care, or other care utilization (visit with a general practitioner, specialist, or other non-oncologist physician) |
| **Michel, 2011^16^**  Michel, 2016^17^; Lupatsch, 2016^18^; Gianinazzi, 2014^19^; Vetsch, 2016^20^; Michel, 2016^17^; Michel, 2017^21^; Hendriks, 2021^22^  *Swiss Childhood Cancer Survivor Study*  Study size: 1075^16^; 314^17^; 1602 survivors, 703 siblings^19^; 410^18^; 189^20^; 183 physicians^21^; 28 survivors, 3 experts^22^  Analysis type: Survey, interview | Switzerland | Proportion of CCS: 100%  Multiple cancer origin | **Sex:** Not significant: Sex was not significantly associated with survivorship care, after adjusting for health beliefs (susceptibility, severity, benefits, barriers, health value, cues to action), demographics (age at study, living in a relationship, education, employment, immigration status, language spoken), and medical variables (age at diagnosis, diagnostic category, treatment, bone marrow transplant, relapse, medical report received, follow-up checklist received).^16^  Sex was not significantly associated with attending survivorship care visits, after adjusting for health beliefs (susceptibility, severity, benefits, barriers, health value, cues to action), demographics (born abroad, language spoken, age at study), and medical characteristics (time since diagnosis, cancer diagnosis, treatment received, and bone marrow transplant).^16,18^  **Education:** In comparison to those with primary school educational attainment (Swiss complementary school, no education), those with secondary school education (vocational training, high school, teacher’s seminar; OR 0.52; CI 0.32, 0.85) were less likely to attend survivorship care, after adjusting for health beliefs (susceptibility, severity, benefits, barriers, health value, cues to action), demographics (sex, age at study, living in a relationship, employment, immigration status, language spoken), and medical variables (age at diagnosis, diagnostic category, treatment, SCT, relapse, medical report received, follow-up checklist received).^16^  **Employment**: Not significant: Employment status was not significantly associated with survivorship care, after adjusting for health beliefs (susceptibility, severity, benefits, barriers, health value, cues to action), demographics (sex, age at study, living in a relationship, education, immigration status, language spoken, age at study), and medical variables (age at diagnosis, diagnostic category, treatment, bone marrow transplant, relapse, medical report received, follow-up checklist received).^16^  **Treatment:** In comparison to those treated with chemotherapy only, those treated with surgery only (OR 0.44; CI 0.20, 0.99) were less likely to attend survivorship care, after adjusting for health beliefs (susceptibility, severity, benefits, barriers, health value, cues to action), demographics (sex, age at study, living in a relationship, education, employment, immigration status, language spoken), and medical variables (age at diagnosis, diagnostic category, treatment, bone marrow transplant, relapse, medical report received, follow-up checklist received). In comparison to those who did not have a bone marrow transplant, those who did have a bone marrow transplant (OR 2.83; CI 1.21, 6.58) were more likely to attend survivorship care, after adjusting for health beliefs (susceptibility, severity, benefits, barriers, health value, cues to action), demographics (sex, age at study, living in a relationship, education, employment, immigration status, language spoken), and medical variables (age at diagnosis, diagnostic category, treatment, relapse, medical report received, follow-up checklist received).^16^  **Age at diagnosis year of diagnosis, or time since diagnosis:** In comparison to those ages 0-4 at diagnosis, those of ages 8-11 (OR 2.06; 1.18, 3.61) or 12+ years of age (OR 4.41; CI 2.56, 7.60) were more likely to attend survivorship care, after adjusting for health beliefs (susceptibility, severity, benefits, barriers, health value, cues to action), demographics (sex, age at study, living in a relationship, education, employment, immigration status, language spoken), and medical variables (diagnostic category, treatment, bone marrow transplant, relapse, medical report received, follow-up checklist received).^16^  In comparison to those who were 5-9 years post-diagnosis, those who were 15+ years post-diagnosis were less likely (OR 0.10; CI 0.05, 0.23) to attend survivorship care visits, after adjusting for health beliefs (susceptibility, severity, benefits, barriers, health value, cues to action), demographics (sex, born abroad, language spoken, age at study), and medical characteristics (cancer diagnosis, treatment received, and bone marrow transplant).^16,18^  In the multivariable regression, longer time since diagnosis (OR 1.20; CI 1.01, 1.42) was associated with non-attendance at survivorship visits, after adjusting for age at study and parent response to question regarding how much survivorship care can help your child (measured treatment control).^16,20^  **Age at study or enrollment, current age:** In comparison to those currently under the age of 25, those of ages 25-29 (OR 0.66; CI 0.42, 1.03), 30-34 (OR 0.27; CI 0.15, 0.49), and 35+ (OR 0.24; CI 0.12, 0.49) were less likely to attend survivorship care, after adjusting for health beliefs (susceptibility, severity, benefits, barriers, health value, cues to action), demographics (sex, living in a relationship, education, employment, immigration status, language spoken), and medical variables (age at diagnosis, diagnostic category, treatment, bone marrow transplant, relapse, medical report received, follow-up checklist received).^16^  In the multivariable regression, older age at study (OR 1.32; CI 1.03, 1.69) was associated with non-attendance at survivorship visits, after adjusting for time since diagnosis and parent response to question regarding how much survivorship care can help your child (measured treatment control).^16,20^  Not significant: Age at study was not significantly associated with attending survivorship care visits, after adjusting for health beliefs (susceptibility, severity, benefits, barriers, health value, cues to action), demographics (sex, born abroad, language spoken), and medical characteristics (time since diagnosis, cancer diagnosis, treatment received, and bone marrow transplant).^16,18^  **Cancer diagnosis:** In comparison to those who did not experience a relapse, those who did experience a relapse (OR 2.78, 1.70, 4.56) were more likely to attend survivorship care, after adjusting for health beliefs (susceptibility, severity, benefits, barriers, health value, cues to action), demographics (sex, age at study, living in a relationship, education, employment, immigration status, language spoken), and medical variables (age at diagnosis, diagnostic category, treatment, bone marrow transplant, medical report received, follow-up checklist received).^16^  Not significant: Cancer diagnosis was not significantly associated with survivorship care, after adjusting for health beliefs (susceptibility, severity, benefits, barriers, health value, cues to action), demographics (sex, age at study, living in a relationship, education, employment, immigration status, language spoken), and medical variables (age at diagnosis, treatment, bone marrow transplant, relapse, medical report received, follow-up checklist received).^16^  Cancer diagnosis was not significantly associated with attending survivorship care visits, after adjusting for health beliefs (susceptibility, severity, benefits, barriers, health value, cues to action), demographics (sex, born abroad, language spoken, age at study), and medical characteristics (time since diagnosis, treatment received, and bone marrow transplant).^16,18^  **Other:** In multivariable analysis among psychologically distressed survivors, self-reported late effects (OR 3.33; CI 1.20, 9.27) and distress severity measured by the Global Severity Index of the Brief Symptom Inventory 18 (OR 1.14; CI 1.03, 1.26) were associated with utilization of mental health care services, after adjusting for current age, sex, language spoken, residing in rural region, cancer diagnosis, relapse, treatment (surgery, chemotherapy, radiotherapy, bone marrow transplant), time since diagnosis, and parental education.^16,19^  Not significant: Living in a relationship (marital status), immigration status (being Swiss born), language spoken (German, French, Italian), medical report received, and follow-up checklist received were not significantly associated with survivorship care, after adjusting for health beliefs (susceptibility, severity, benefits, barriers, health value, cues to action), demographics (sex, age at study, education, employment, immigration status, language spoken), and medical variables (age at diagnosis, diagnostic category, treatment, bone marrow transplant, relapse, medical report received, follow-up checklist received).^16^ | **Survivorship care:**  Utilization of survivorship care services, care plans, or models of care (survivorship care versus non-survivorship/no care; preference of survivorship care model; survivorship care versus non-survivorship care)  **Psychosocial:**  Psychological  **Health services/ economics:**  Primary care, specialty care, or other care utilization (specialist or mental health services) |
| **Milam, 2015^23^**  Miller, 2018^24^; Miller, 2017^25^; Cousineau, 2019^26^; Slaughter, 2020^27^; Tobin, 2020^28^; Sleight, 2019^29^; Slaughter, 2020^30^; Ochoa, 2022^31^; Ochoa, 2022^32^; Wojcik, 2021^33^; Milam, 2021^34^; Mobley, 2021^35^  *Project Forward*  Study size: 1106^34,35^; 193^23-25,29^; 235^26^; 129 survivor/parent dyads^30^; 160 survivor/parent dyads^31,32^; 128 melanoma survivors^33^  Analysis type: Survey, analytic study | USA | Proportion of CCS: 100%  Multiple cancer origin | **Race/ethnicity:** Hispanic survivors were less likely to access survivorship care in comparison to white, non-Hispanic survivors (OR 0.33; CI 0.11, 0.96), after adjusting for sex, health insurance, treatment intensity, family influence on healthcare decisions, having a regular doctor, healthcare self-efficacy, and post traumatic growth.^23^  In comparison to those who were white, survivors of other races/ethnicities were less likely (OR 0.67; CI 0.43, 1.06) to access survivorship care, after adjusting for socioeconomic status at diagnosis, socioeconomic status at survey, age at diagnosis, education, treatment intensity, sex, age at survey, and insurance.^23,28^  In multivariable models and in comparison to non-Hispanic white survivors, those who were Hispanic (OR 0.69; CI 0.51, 0.95) or of other race/ethnicity: (OR 0.69; 0.48, 0.99) were less likely to access survivorship care, after adjusting for years since diagnosis, current age, sex, socioeconomic status, health insurance coverage, high levels of depressive symptoms, number of late effects, treatment intensity, received a written treatment summary, reported having a doctor for regular (non-cancer) care, discussed cancer-related follow-up care with a doctor in the last two years, knowledge of the need for life-long survivorship care, health care self-efficacy, and family influence of health care decisions.^23,34^  **Income:** Not significant: Socioeconomic status at diagnosis, socioeconomic status at survey, and average socioeconomic status from diagnosis to survey was not significantly associated with access survivorship care, after adjusting for age at diagnosis, education, treatment intensity, sex, age at survey, and insurance.^23,28^  In the multivariable model, socioeconomic status was not significantly associated with access to survivorship care, after adjusting for years since diagnosis, current age, sex, race and ethnicity, health insurance, high levels of depressive symptoms, number of late effects, treatment intensity, received a written treatment summary, reported having a doctor for regular (non-cancer) care, discussed cancer-related follow-up care with a doctor in the last two years, knowledge of the need for life-long survivorship care, health care self-efficacy, and family influence of health care decisions.^23,34^  **Sex:** Female survivors were more likely (OR 1.34; CI 1.11, 1.62) to access survivorship care, after adjusting for socioeconomic status at diagnosis, socioeconomic status at survey, age at diagnosis, education, treatment intensity, race/ethnicity, age at survey, and insurance.^23,28^  Female survivors were more likely to have a recent survivorship visit (OR 4.26; CI 1.60, 11.40), after adjusting for current age, time since diagnosis, treatment intensity, health insurance status, Spanish-speaking Hispanic parent, English-speaking Hispanic parent, and English-speaking non-Hispanic parent.^23,31^  Not significant: There was not a significant association between sex and survivorship care, after adjusting for race/ethnicity, health insurance, treatment intensity, family influence on healthcare decisions, having a regular doctor, healthcare self-efficacy, and post traumatic growth.^23^  **Education:** Not significant: Education did not have a significant relationship with survivorship care, after adjusting for socioeconomic status at diagnosis, socioeconomic status at survey, age at diagnosis, treatment intensity, sex, race/ethnicity, age at survey, and insurance.^23,28^  **Insurance:** Survivors who had any health insurance were more likely (OR 3.40; CI 1.10, 8.41) to access survivorship care in comparison to those who were uninsured, after adjusting for sex, race/ethnicity, treatment intensity, family influence on healthcare decisions, having a regular doctor, healthcare self-efficacy, and post traumatic growth.^23^  Those who were uninsured (in comparison to those who were privately covered) were less likely to have survivorship care (OR 0.28; CI 0.16, 0.49), after adjusting for socioeconomic status at diagnosis, socioeconomic status at survey, age at diagnosis, education, treatment intensity, sex, race/ethnicity, and age at survey. In comparison to those who were privately insured at diagnosis, survivors who were uninsured were less likely to access survivorship care (OR 0.26; CI 0.14, 0.48), after adjusting for average socioeconomic status from diagnosis to survey, age at diagnosis, treatment intensity, sex, race/ethnicity, and age at survey.^23,28^  In comparison to those who were privately insured, those who were uninsured were 4.3 times as likely to have no regular provider for cancer-related follow-up care (CI 1.9, 9.4), 3.3 times as likely to lack a regular provider for non-cancer care (CI 1.6, 6.9), 5.3 times as likely to lack both sources of care (CI 2.1, 13.5), 3.9 times as likely to have had no primary care visit (CI 1.8, 8.2), and 4.5 times as likely to have not seen a cancer specialist (CI 2.1, 9.5). In comparison to those who were privately covered, those with public insurance were 2.5 times as likely to report no regular source of primary care (CI 1.1, 5.4) and 2.8 times as likely not to have made a primary care visit in the past 2 years compared with those with private coverage. Both models adjusted for sex, ethnicity, age, socioeconomic status, and treatment intensity.^23,26^  Survivors who were insured were more likely to have had a recent survivorship visit (OR 4.20; CI 1.65, 10.67), after adjusting for current age, time since diagnosis, sex, treatment intensity, Spanish-speaking Hispanic parent, English-speaking Hispanic parent, and English-speaking non-Hispanic parent.^23,31^  In the multivariable model and in comparison to survivors who were not insured, those who were insured (OR 2.06; CI 1.28, 3.32) were more likely to access survivorship care, after adjusting for years since diagnosis, current age, sex, race and ethnicity, socioeconomic status, high levels of depressive symptoms, number of late effects, treatment intensity, received a written treatment summary, reported having a doctor for regular (non-cancer) care, discussed cancer-related follow-up care with a doctor in the last two years, knowledge of the need for life-long survivorship care, health care self-efficacy, and family influence of health care decisions.^23,34^  In the multivariable model, survivors who experienced a change in insurance coverage from diagnosis to survivorship resulting in a gain in coverage (b -0.05; SE 0.02) or a loss in coverage (b -0.15; SE 0.04) had a decrease in the predicted probability of having a survivorship care visit in the prior two years; however, those covered by Medicaid and/or Medicare at diagnosis had an increase in the probability of having a survivorship care visit in the prior two years (b 0.05; SE 0.02); all models were adjusted for current age, sex, race and ethnicity, marital status, educational attainment, receipt of supplemental income, insurance at diagnosis, insurance understanding, socioeconomic status, treatment intensity, children, employment status, difficulty with a referral to a specialist, ability to see a doctor when needed, friend/family influence of health decisions, cancer diagnosis, and self-rated health.^23,34^  Not significant: In comparison to those who were privately insured, those who were uninsured did not significantly differ regarding having a regular source of cancer-related follow-up care, after adjusting for sex, ethnicity, age, socioeconomic status, and treatment intensity. In comparison to those who were privately insured, those who were publicly insured did not significantly differ regarding having a regular source of cancer-related follow-up care, no regular source of both cancer and noncancer care, no cancer visit within the past two years, and at least one emergency department visit in the prior two years, after adjusting for sex, ethnicity, age, socioeconomic status, and treatment intensity.^23,26^  Those who were publicly covered or covered by other insurance types (in comparison to those who were privately covered) did not have a significant association with survivorship care, after adjusting for socioeconomic status at diagnosis, socioeconomic status at survey, age at diagnosis, education, treatment intensity, sex, race/ethnicity, and age at survey. In comparison to those who were privately insured at diagnosis, survivors who were publicly covered or had other types of coverage did not have a significant relationship with survivorship care, after adjusting for average socioeconomic status from diagnosis to survey, age at diagnosis, treatment intensity, sex, race/ethnicity, and age at survey.^23,28^  **Treatment:** For each increase in the level of treatment intensity (from 1-4), survivors were more likely (OR 1.83; CI 1.09, 3.06) to access survivorship care, after adjusting for sex, race/ethnicity, health insurance, family influence on healthcare decisions, having a regular doctor, healthcare self-efficacy, and post traumatic growth.^23^  Greater treatment intensity was associated with increased odds of having a recent survivorship visit (OR 1.89; CI 1.07, 3.31), after adjusting for current age, time since diagnosis, sex, health insurance status, Spanish-speaking Hispanic parent, English-speaking Hispanic parent, and English-speaking non-Hispanic parent.^23,31^  **Age at diagnosis year of diagnosis, or time since diagnosis:** For each year increase in age at diagnosis, survivors were more likely (OR 1.12; CI 1.08, 1.16) to access survivorship care, after adjusting for socioeconomic status at diagnosis, socioeconomic status at survey, education, treatment intensity, sex, race/ethnicity, age at survey, insurance.^23,28^  For each year increase in age at diagnosis, survivors were less likely (OR 0.88; CI 0.84, 0.92) to access survivorship care, after adjusting for current age, sex, race and ethnicity, socioeconomic status, health insurance coverage, high levels of depressive symptoms, number of late effects, treatment intensity, received a written treatment summary, reported having a doctor for regular (non-cancer) care, discussed cancer-related follow-up care with a doctor in the last two years, knowledge of the need for life-long survivorship care, health care self-efficacy, and family influence of health care decisions.^23,34^  **Age at study or enrollment, current age:** For each year increase in current age at survey, survivors were less likely (OR 0.83; CI 0.79, 0.86) to access survivorship care, after adjusting for socioeconomic status at diagnosis, socioeconomic status at survey, age at diagnosis, education, treatment intensity, sex, race/ethnicity, insurance.^23,28^  Survivors 21 years of age or older were associated with decreased odds of having a recent survivorship visit (OR 0.32; CI 0.13, 0.79), after adjusting for treatment intensity, time since diagnosis, sex, health insurance status, Spanish-speaking Hispanic parent, English-speaking Hispanic parent, and English-speaking non-Hispanic parent.^23,31^  In the multivariable model and in comparison to those currently 18-20 years of age, survivors ages 21-25 (OR 0.65; CI 0.50, 0.85), 26-30 (OR 0.32; CI 0.22, 0.48), and 31-39 (OR 0.35; CI 0.24, 0.50) were less likely to access survivorship care, after adjusting for years since diagnosis, sex, race and ethnicity, socioeconomic status, health insurance coverage, high levels of depressive symptoms, number of late effects, treatment intensity, received a written treatment summary, reported having a doctor for regular (non-cancer) care, discussed cancer-related follow-up care with a doctor in the last two years, knowledge of the need for life-long survivorship care, health care self-efficacy, and family influence of health care decisions.^23,34^  **Other:** In the multivariable model and in comparison to survivors who did not report any late effects, those who did report one late effect (OR 1.41; CI 1.08, 1.81) or two or more late effects (OR 1.54; CI 1.23, 1.92) were more likely to access survivorship care, after adjusting for years since diagnosis, current age, sex, race and ethnicity, socioeconomic status, health insurance coverage, high levels of depressive symptoms, treatment intensity, received a written treatment summary, reported having a doctor for regular (non-cancer) care, discussed cancer-related follow-up care with a doctor in the last two years, knowledge of the need for life-long survivorship care, health care self-efficacy, and family influence of health care decisions.^23,34^  Not significant: There was not a significant association between family influence of health decisions, regular non-cancer provider, healthcare self efficacy, and post-traumatic growth with access survivorship care, after adjusting for sex, race/ethnicity, health insurance, treatment intensity, family influence on healthcare decisions, having a regular doctor, healthcare self-efficacy, and post traumatic growth.^23^  In the multivariable model, family influence of health care decisions was not significantly associated with access to survivorship care, after adjusting for years since diagnosis, current age, sex, race and ethnicity, socioeconomic status, health insurance, high levels of depressive symptoms, number of late effects, treatment intensity, received a written treatment summary, reported having a doctor for regular (non-cancer) care, discussed cancer-related follow-up care with a doctor in the last two years, knowledge of the need for life-long survivorship care, and health care self-efficacy.^23,34^ | **Survivorship care:** Utilization of survivorship care services, care plans, or models of care (intent to seek survivorship care, knowledge of the need for survivorship care)  **Psychosocial:** Psychological  **Health services/ economics:**  Financial hardship, costs, and resource utilization (non-cancer related regular source of care, PCP visit in prior two years, emergency department use) |
| **Nathan, 2016^36^**  Marr, 2017^37^; Sutradhar, 2015^38^  *Pediatric Oncology Group of Ontario Networked Information System (POGONIS)*  Study size: 3912 survivors^36^; 1811 survivors^37^; 3912 survivors^38^  Analysis type: Analytic study | Canada | Proportion of CCS: 100%  Multiple cancer origin | **Income:** Survivors from areas of higher socioeconomic status areas were more likely to attend survivorship clinic (Relative Rate, RR 1.27; CI 1.06, 1.53), after adjusting for sex, age at diagnosis, cancer diagnosis, diagnosis prior to 1999, treatment intensity, cyclophosphamide equivalent dose, doxorubicin equivalent dose, radiation, secondary malignancy or relapse before index date, survivorship clinic model, distance to survivorship clinic, and complete history/physical exam by a primary care provider.^36^  Not significant: Neighborhood deprivation quintile was not significantly associated with an increased rate of echocardiogram surveillance adherence at 1-year, 2-years, and 5-years, when controlling for survivorship clinic attendance, age at diagnosis, sex, year of diagnosis, cancer diagnosis, doxorubicin-equivalent dose, radiotherapy to chest, intensity of treatment, bone marrow transplant, prior recurrence of disease, distance from survivorship clinic, and annual physical exam with a primary care provider.^36,37^  **Underserved or rural:** Those who traveled 25-49 kilometers (RR 0.88; CI 0.76, 1.01), 50-99 kilometers (RR 0.77; CI 0.65, 0.91), >100 kilometers (RR 0.48; CI 0.39, 0.60) had a decreased likelihood of survivorship clinic attendance, after adjusting for sex, age at diagnosis, socioeconomic status, cancer diagnosis, diagnosis prior to 1999, treatment intensity, cyclophosphamide equivalent dose, doxorubicin equivalent dose, radiation, secondary malignancy or relapse before index date, survivorship clinic model, and complete history/physical exam by a primary care provider.^36^  Living in a rural area was associated with an increased echocardiogram adherence for survivors whose surveillance occured every 2- and 5-years (2-year: RR 2.31; CI 1.27, 4.18 and 5-year: RR 2.03; CI 1.24, 3.32), when controlling for survivorship clinic attendance, age at diagnosis, sex, year of diagnosis, cancer diagnosis, doxorubicin-equivalent dose, radiotherapy to chest, intensity of treatment, bone marrow transplant, prior recurrence of disease, neighborhood socioeconomic status, distance from survivorship clinic, and annual physical exam with a primary care provider.^36,37^  Not significant: Living in a non-major urban area (1-, 2-, and 5-years) or a rural area (1-year) was not significantly associated with an increased rate of echocardiogram surveillance adherence for survivors at the specified interval, and distance travelled from home to the survivorship clinic was not significantly associated with the rate of echocardiogram surveillance adherence for survivors at 1-, 2-, and 5-years. All models adjusted for survivorship clinic attendance, age at diagnosis, sex, year of diagnosis, cancer diagnosis, doxorubicin-equivalent dose, radiotherapy to chest, intensity of treatment, bone marrow transplant, prior recurrence of disease, neighborhood socioeconomic status, distance from survivorship clinic, and annual physical exam with a primary care provider.^36,37^  **Sex:** Females were more likely to attend survivorship clinic (RR 1.18; CI 1.07, 1.31), after adjusting for age at diagnosis, socioeconomic status, cancer diagnosis, diagnosis prior to 1999, treatment intensity, cyclophosphamide equivalent dose, doxorubicin equivalent dose, radiation, secondary malignancy or relapse before index date, survivorship clinic model, distance to survivorship clinic, and complete history/physical exam by a primary care provider.^36^  Survivors who were female were more likely to be up-to-date for scheduled echocardiogram screening occuring every 1-year (RR 1.76; CI 1.10, 2.82), 2-years (RR 1.41; CI 1.06, 1.88), and 5-years (RR 1.31; CI 1.03, 1.68) in comparison to males, after adjusting for rate of survivor clinic attendance (per 10-year period), age at diagnosis, year of diagnosis, cancer diagnosis, diagnosis from 2003-2005, doxorubicin equivalent dose, radiation, treatment intensity, bone marrow transplant, prior recurrence, neighborhood deprivation quintile, rurality, distance to survivorship clinic, and complete history/physical exam by a primary care provider.^36,37^  **Treatment:** Increased survivorship clinic attendance was associated with treatment intensity score of 2 (RR 1.68; CI 1.26, 2.25), score of 3 (RR 2.21; CI 1.65, 2.96), or score of 4 (RR 1.69; CI 1.10, 2.58), compared to those with a score of 1 (least intense treatment); higher cyclophosphamide equivalent doses as those who received 4000-7999mg/m2 (RR 1.29; CI 1.08, 1.54) or those who received >8000mg/m2 (RR 1.47; CI 1.25, 1.73), compared to those who did not receive cyclophosphamide; and radiation to the brain only (RR 1.44; CI 1.23, 1.67), chest only (RR 1.59; CI 1.28, 1.97), or other sites (RR 1.31; CI 1.13, 1.53), compared to those who did not receive treatment with radiation. All models adjusted for sex, age at diagnosis, socioeconoomic status, cancer diagnosis, cyclophosphamide equivalent dose, doxorubicin equivalent dose, radiation, secondary malignancy or relapse before index date, survivorship clinic model, distance to survivorship clinic, and complete history/physical exam by a primary care provider.^36^  For the echocardiogram surveillance group occuring every 2-years, those who received a doxorubicin-equivalent dose of >250mg/m2 had an increased rate of echocardiogram surveillance adherence (RR 2.28; CI 1.53, 3.39), in comparison to those who received a doxorubicin-equivalent dose of 1-249mg/m2, when adjusting for survivorship clinic attendance, age at diagnosis, sex, year of diagnosis, cancer diagnosis, radiotherapy to chest, intensity of treatment, bone marrow transplant, prior recurrence of disease, neighborhood socioeconomic status, living in an urban or rural area, distance from survivorship clinic, and annual physical exam with a primary care provider.^36,37^  Not significant: Survivorship clinic attendance was not statistically associated with treatment with doxorubicin, after adjusting for sex, age at diagnosis, socioeconoomic status, cancer diagnosis, cyclophosphamide equivalent dose, radiation, secondary malignancy or relapse before index date, survivorship clinic model, distance to survivorship clinic, and complete history/physical exam by a primary care provider.^36^  Receiving radiation to the chest, intensity of treatment, and bone marrow transplantation were not significantly associated with the rate of echocardiogram surveillance adherence for survivors at 1-, 2-, and 5-years of screening, when controlling for survivorship clinic attendance, age at diagnosis, sex, year of diagnosis, cancer diagnosis, doxorubicin-equivalent dose, radiation to the chest, intensity of treatment, bone marrow transplant, prior recurrence of disease, neighborhood deprivation quintile, urban/rural location, distance from survivorship clinic, and annual physical exam with a primary care provider.^36,37^  **Age at diagnosis year of diagnosis, or time since diagnosis:** Decreased survivorship clinic attendance was associated with a diagnosis prior to 1999 (RR 0.74; CI 0.63, 0.86) compared to those diagnosed after 1999, after adjusting for sex, age at diagnosis, socioeconoomic status, cancer diagnosis, treatment intensity, cyclophosphamide equivalent dose, doxorubicin equivalent dose, radiation, secondary malignancy or relapse before index date, survivorship clinic model, distance to survivorship clinic, and complete history/physical exam by a primary care provider.^36^  Survivors who were diagnosed between 2003-2005 had an increase in the rate of echocardiogram surveillance adherence for those occuring at 1-year (RR 0.20; CI 0.08, 0.51) and 2-years (RR 0.54; CI 0.31, 0.93), when adjusting for survivorship clinic attendance, age at diagnosis, sex, cancer diagnosis, doxorubicin-equivalent dose, radiation to the chest, intensity of treatment, bone marrow transplant, prior recurrence of disease, neighborhood deprivation quintile, urban/rural location, distance from survivorship clinic, and annual physical exam with a primary care provider.^36,37^  Not significant: Survivorship clinic attendance was not statistically associated with age at diagnosis, after adjusting for sex, socioeconoomic status, cancer diagnosis, cyclophosphamide equivalent dose, doxorubicin equivalent dose, radiation, secondary malignancy or relapse before index date, survivorship clinic model, distance to survivorship clinic, and complete history/physical exam by a primary care provider.^36^  Age at diagnosis was not significantly associated with the rate of echocardiogram surveillance adherence for survivors at 1-, 2-, and 5-years, when controlling for survivorship clinic attendance, age at diagnosis, sex, year of diagnosis, cancer diagnosis, doxorubicin-equivalent dose, radiation to the chest, intensity of treatment, bone marrow transplant, prior recurrence of disease, neighborhood deprivation quintile, urban/rural location, distance from survivorship clinic, and annual physical exam with a primary care provider.^36,37^  **Cancer diagnosis:** Decreased survivorship clinic attendance was associated with a diagnosis of a brain tumor (RR 0.63; CI 0.50, 0.77) or other cancer (RR 0.67; 0.54, 0.84) compared to ALL, after adjusting for sex, age at diagnosis, socioeconoomic status, diagnosis prior to 1999, treatment intensity, cyclophosphamide equivalent dose, doxorubicin equivalent dose, radiation, secondary malignancy or relapse before index date, survivorship clinic model, distance to survivorship clinic, and complete history/physical exam by a primary care provider.^36^  Survivors who were diagnosed with AML (2-year surveillance: RR 2.74; CI 1.44, 5.19) and lymphoma (2-year surveillance: RR 1.56; CI 1.06, 2.30 and 5-year surveillance: RR 1.34; CI 1.00-1.79) were more likely to be up-to-date for scheduled screening compared to those who were diganosed with ALL, when controlling for survivorship clinic attendance, age at diagnosis, sex, year of diagnosis, doxorubicin-equivalent dose, radiation to the chest, intensity of treatment, bone marrow transplant, prior recurrence of disease, neighborhood deprivation quintile, urban/rural location, distance from survivorship clinic, and annual physical exam with a primary care provider.^36,37^  Not significant: Survivorship clinic attendance was not statistically associated with diagnosis with a secondary malignancy or relapse for index date, after adjusting for sex, age at diagnosis, socioeconoomic status, cancer diagnosis, cyclophosphamide equivalent dose, doxorubicin equivalent dose, radiation, secondary malignancy or relapse before index date, survivorship clinic model, distance to survivorship clinic, and complete history/physical exam by a primary care provider.^36^  At the 1-year screening interval, there was not a significant relationship between adherence to echocardiogram screening surveillance and cancer diagnosis, when controlling for age at diagnosis, sex, year of diagnosis, doxorubicin-equivalent dose, radiation to the chest, intensity of treatment, bone marrow transplant, prior recurrence of disease, neighborhood deprivation quintile, urban/rural location, distance from survivorship clinic, and annual physical exam with a primary care provider.^36,37^  **Other:** Survivors seen in a clinic with a combined pediatric/adult program (RR 0.85; CI 0.76, 0.96) were less likely to have a survivorship clinic visit, after adjusting for sex, age at diagnosis, socioeconomic status, cancer diagnosis, diagnosis prior to 1999, treatment intensity, cyclophosphamide equivalent dose, doxorubicin equivalent dose, radiation, secondary malignancy or relapse before index date, distance traveled to survivorship clinic, and complete history/physical exam by a primary care provider. Those who had a complete history/physical exam by a primary care provider (RR 1.16; CI 1.07, 1.26) were more likely to have a survivorship clinic visit in comparison to those who did not have a complete history/physical exam conducted by their primary care provider, after adjusting for sex, age at diagnosis, socioeconomic status, cancer diagnosis, diagnosis prior to 1999, treatment intensity, cyclophosphamide equivalent dose, doxorubicin equivalent dose, radiation, secondary malignancy or relapse before index date, distance traveled to survivorship clinic, and survivorship clinic model.^36^  Survivors who had survivorship clinic visits occuring 1-2 times (RR 3.36; CI 1.82, 6.22), 3-4 times (RR 6.96; CI 3.54, 13.69), or >5 times (RR 10.55; CI 5.71, 19.49) per 10-year period were more likely to be up-to-date for scheduled echocardiogram screening occuring every 1-year in comparison to those who had no survivorship clinic visits. Those who had survivorship clinic visits occuring 1-2 times (RR 2.38; CI 1.56, 3.62), 3-4 times (RR 2.10; CI 1.39, 3.18), or >5 times (RR 3.32; CI 2.29, 4.81) per 10-year period were more likely to be up-to-date for scheduled echocardiogram screening occuring every 2-years in comparison to those who had no survivorship clinic visits. Survivors who had survivorship clinic visits occuring 1-2 times (RR 1.81; CI 1.28, 2.55), 3-4 times (RR 1.91; CI 1.34, 2.72), or >5 times (RR 2.30; CI 1.66, 3.18) per 10-year period were more likely to be up-to-date for scheduled echocardiogram screening occuring every 5-years in comparison to those who had no survivorship clinic visits. All models were adjusted for age at diagnosis, sex, year of diagnosis, cancer diagnosis, doxorubicin equivalent dose, radiation to the chest, treatment intensity, bone marrow transplant, prior recurrence, neighborhood deprivation quintile, rural/urban residence, and distance to survivorship clinic. Survivors who had an annual physical exam with a primary care provider (RR 2.78; CI 1.67, 4.76) were more likely to be up-to-date for scheduled echocardiogram screening occuring every 1-year in comparison to those who had no annual physical exam with a primary care provider, after adjusting for rate of survivorship clinic attendance and the variables listed above. ^36,37^  Not significant: Survivors who had an annual physical exam with a primary care provider did not have a statistically significant change in the rate of scheduled echocardiogram screening occuring every 2- and 5-years in comparison to those who had no annual physical exam with a primary care provider, after adjusting for rate of survivorship clinic attendance, age at diagnosis, sex, year of diagnosis, cancer diagnosis, doxorubicin equivalent dose, radiation to the chest, treatment intensity, bone marrow transplant, prior recurrence, neighborhood deprivation quintile, rural/urban residence, and distance to survivorship clinic.^36,37^ | **Survivorship care:** Utilization of survivorship care services, care plans, or models of care (survivorship visit; adherence to risk-based cardiovascular screening)  **Health services/ economics:**  Primary care, specialty care, or other care utilization (emergency department visits) |
| **Oeffinger, 2004^39^**  Kirchhoff, 2013^40^; Casillas, 2015^41^; Nathan, 2008^42^; Caplin, 2017^43^; Casillas, 2011^44^; Castellino, 2005^45^; Geller, 2019^46^; Kaste, 2009^47^; Kirchhoff, 2018^48^; Nipp, 2017^49^; Recklitis, 2010^50^; Yeazel, 2004^51^  *Childhood Cancer Survivor Study*  Study size: 9434^39^; 32^40^; 6176^41^; 8522^42^; 7899^43^; 8425^44^; 8767^45^; 728^46^; 9308^47^; 394 survivors, 128 siblings^48^; 580 survivors, 173 siblings^49^; 9126 survivors, 2968 siblings^50^; 9434 survivors, 3858 siblings^51^  Analysis type: Survey, interview, analytic study | Multiple countries | Proportion of CCS: 100%  Multiple cancer origin | **Race/ethnicity:** In comparison to non-Hispanic white survivors, minority survivors were less likely (OR 0.79; CI 0.64, 0.96) to report absence of a cancer center medical visit, after adjusting for age at study, sex, educational attainment, health insurance, health status, concern for future health, and high-risk treatment.^39^  In comparison to white non-Hispanic CCS, survivors who were black (OR 2.1; CI 1.3, 3.3) were more likely to report general care rather than risk-based survivorship care and those who were of other races (OR 0.8; CI 0.7, 1.0) were less likely to report general care versus risk-based survivorship care, after adjusting for sex, current age, age at interview, annual household income, educational attainment, employment status, insurance coverage, poor emotional health, cancer-related anxiety, cancer-related pain, poor physical health, and grade of chronic disease.^39,42^  Survivors who were from the other race/ethnicity group who reported some form of medical care at baseline (either survivor-focused or general) experienced an increased risk of reporting no care at follow-up (OR 2.1; CI 1.2, 3.7) compared to white non-Hispanic CCS, after adjusting for sex, annual household income, chronic disease status, and education.^39,41^  Uninsured non-Hispanic white survivors were less likely to report survivor focused-care for a cancer-related visit (OR 0.82, 0.74-0.90) and a cancer center visit (OR 0.79; 0.66-0.94) compared to privately-insured non-Hispanic white survivors. Publicly insured non-Hispanic white survivors were more likely to report a cancer-related visit (OR 1.20, 1.08-1.34) and a cancer center visit (OR 1.31, 1.06-1.62) compared to privately-insured non-Hispanic white survivors. Publicly insured Hispanic survivors were more likely to report a cancer-related visit (OR 1.41, 1.12-1.78) and a cancer center visit (OR 1.88, 1.28-2.77) compared to privately-insured Hispanic survivors. All models adjusted for age, sex, household income, highest level of educational attainment, and grade 3 or 4 chronic condition.^39,44^  After adjusting for age, cancer diagnosis, health insurance, household income, and highest level of educational attainment, Hispanic survivors were more likely to report a cancer center visit (females: OR 1.5, 1.1-2.0; males: OR 1.7, 1.2 -2.3) comparison to non-Hispanic white survivors, and Hispanic males were more likely to report cancer-related medical visit (OR 1.3, 1.0-1.8) compared to non-Hispanic white survivors.^39,45^  In multivariable analysis, survivors who were non-white (0.67; 0.52, 0.86) were less likely to report a skin exam, after adjusting for sex, age at interview, educational attainment, concern about future health, cancer-related pain, having a cancer treatment summary, having medical care within the past two years, and having a cancer-related visit within the past two years.^39,52^  Not significant: Race/ethnicity was not significantly associated with absence of a cancer-related medical visit, after adjusting for age at study, sex, educational attainment, health insurance, health status, concern for future health, and high-risk treatment.^39^  **Income:** In comparison to survivors' reporting current annual household income more than $60,000, survivors who had income less than $20,000 (RR 1.6, 1.2-2.3) or between $20,000-$39,999 (RR 1.4, 1.0-1.9) who reported some form of medical care at baseline (either survivor-focused or general) experienced an increased risk of reporting no care at follow-up, after adjusting for sex, race/ethnicity, chronic disease status, and education.^39,41^  After adjusting for individual factors (sex, race/ethnicity, age, household income, education, employment status, insurance status, poor emotional health, cancer-related anxiety, cancer-related pain, poor physical health, and chronic medical condition status), those from areas with population-based higher median income (OR 1.05, 1.01-1.09) were associated with increased risk-based survivor-focused care versus general care among all participants.^39,43^  Not significant: There was not a significant relationship between annual household income and odds of reporting general care rather than risk-based survivorship care, after adjusting for sex, race/ethnicity, current age, age at interview, insurance coverage, employment status, poor emotional health, cancer-related anxiety, cancer-related pain, poor physical health, and grade of chronic disease.^39,42^  **Underserved or rural:** In multivariable regression and after adjusting for individual factors, the number of Childhood Cancer Survivor Study centers (OR 1.12; CI 1.04, 1.20) and the number of physicians/surgeons (OR 1.06; CI 1.01, 1.11) within the geographic area was associated with greater odds of receiving risk-based survivor-focused medical care among U.S. residents.^39,43^  **Sex:** In comparison to female survivors, males were more likely to report were more likely to report absence of a cancer related medical visit (1.18, 1.06, 1.30), after adjusting for age at study, ethnicity, educational attainment, health insurance, health status, concern for future health, and high-risk treatment. In comparison to female survivors, males were more likely to report were more likely to report absence of a cancer center medical visit (OR 1.15; CI 1.01, 1.30), after adjusting for age at study, ethnicity, educational attainment, health insurance, health status, concern for future health, and high-risk treatment.^39^  In multivariable regression models, female survivors were more likely to report general survivor care (OR 1.48; CI 1.35, 1.62) and risk based survivor care (OR 1.35; CI 1.25, 1.46) compared to male survivors, after adjusting for age, sex, insurance coverage, household income, body mass index, cancer diagnosis, neurocognitive problems, and antidepressant use.^39,53^  In multivariable analysis, males treated with cranial radiation (OR 1.27; CI 1.12, 1.44) were 27 percent more likely to report a recent dental visit than male cancer survivors not treated with cranial radiation, after adjusting for age, ethnicity, education, health insurance status, concern for future health, and cranial radiation therapy.^39,51^  Not significant: The multivariable model predicting general care (in comparison to risk-based survivor-focused care) was not significantly associated with male sex, after adjusting for race/ethnicity, current age, annual household income, educational attainment, employment status, health insurance, poor emotional health, cancer-related anxiety, cancer-related pain, poor physical health, and chronic disease status.^39,42^  **Education:** In comparison to survivors who graduated from college, those with a high school degree or some college were more likely to report absence of a cancer related medical visit (OR 1.16; CI 1.03, 1.31), after adjusting for age at study, sex, ethnicity, health insurance, health status, concern for future health, and high-risk treatment. In comparison to survivors who graduated from college, those with a high school degree or some college were more likely to report absence of a cancer related medical visit (OR 1.25; CI 1.08, 1.44), after adjusting for age at study, sex, ethnicity, health insurance, health status, concern for future health, and high-risk treatment.^39^  In comparison to survivors' who were college graduates, survivors who had less than a high school education (OR 2.5; CI 1.6, 3.8) or who were high school graduates (OR 2.0; CI 1.5, 2.7) who reported some form of medical care at baseline (either survivor-focused or general) experienced an increased risk of reporting no care at follow-up, after adjusting for sex, race/ethnicity, annual household income, and chronic disease status.^39,41^  In comparison to survivors' who were high school or less graduates, survivors who had postgraduate education (OR 3.63; CI 1.24, 10.66) experienced an increased risk of reporting physician skin exam, after sex, education, age (years), race/ethnicity, skin type, age at diagnosis, diagnosis, chemotherapy (yes/no), highest CTCAE grade chronic condition, maximum radiotherapy dose, patient activation.^39,46^  In multivariable analysis, having a high school education or less (OR 0.87; CI 0.77, 0.98) were associated with a decreased likelihood of Papanicolaou smear adherence, after adjusting for race, age at interview, marital status, education, insurance status, and emotional health.^39,52^  In multivariable analysis, survivors having a college education or higher (OR 1.24; CI 1.08, 1.42) were associated with an increased likelihood of reporting a skin exam, after adjusting for sex, race, age at interview, concern about future health, cancer-related pain, having a cancer treatment summary, having medical care within the past two years, and having a cancer-related visit within the past two years.^39,52^  Not significant: There was not a significant relationship between educational attainment and odds of reporting general care rather than risk-based survivorship care, after adjusting for sex, race/ethnicity, current age, age at interview, annual household income, insurance coverage, poor emotional health, cancer-related anxiety, cancer-related pain, poor physical health, and grade of chronic disease.^39,42^  **Employment**: In comparison to employed survivors, unemployed survivors were less likely to report general care (OR 0.7; CI 0.6, 0.8) rather than risk-based survivorship care, after adjusting for sex, race/ethnicity, current age, age at interview, annual household income, educational attainment, insurance coverage, poor emotional health, cancer-related anxiety, cancer-related pain, poor physical health, and grade of chronic disease.^39,42^  **Insurance:** In comparison to survivors who had insurance, those who were uninsured were more likely to report absence of a cancer related medical visit (OR 1.64; CI 1.40, 1.92) or a cancer center medical visit (OR 1.41; CI 1.16, 1.70), after adjusting for age at study, sex, ethnicity, educational attainment, health status, concern for future health, and high-risk treatment.^39^  In comparison to survivors who had insurance from the U.S., those who were U.S.-based and uninsured experienced an increased odds (OR 1.7; 1.3, 2.2) of having general care (rather than risk-based survivorship care) and those who were Canadian residents were less likely to have general care (versus risk-based survivorship care; OR 0.8; CI 0.6, 1.0), after adjusting for sex, race/ethnicity, current age, age at interview, annual household income, educational attainment, employment status, poor emotional health, cancer-related anxiety, cancer-related pain, poor physical health, and grade of chronic disease.^39,42^  In adjusted models, uninsured survivors were less likely than privately insured to report a cancer-related (OR 0.83; CI 0.75, 0.91) or a cancer center visit (OR 0.83; CI 0.71, 0.98) and, in contrast, publicly insured survivors were more likely to report a cancer-related (OR 1.22; CI 1.11, 1.35) or a cancer center visit (OR 1.41; CI 1.18, 1.70) than privately insured survivors, when adjusting for age, sex, household income, highest level of educational attainment, race/ethnicity, and grade 3 or 4 chronic condition.^39,44^  In multivariable regression models, insured survivors were more likely to report general survivor care (OR 2.24; CI 1.82, 2.75) and risk based survivor care (OR 2.65; CI 2.13, 3.30) compared to uninsured survivors, after adjusting for age, sex, insurance coverage, household income, body mass index, cancer diagnosis, neurocognitive problems, and antidepressant use.^39,53^  In multivariable analysis, being uninsured (OR 0.85; CI 0.74, 0.97) was associated with a decreased likelihood of Papanicolaou smear adherence, after adjusting for race, age at interview, marital status, education, insurance status, and emotional health.^39,52^  **Treatment:** In comparison to survivors who did not receive high-risk treatment, those who did receive high-risk treatment were less likely to report absence of cancer-related medical visits (OR 0.59; CI 0.52, 0.65) or cancer center medical visits (OR 0.45; CI 0.39, 0.51), after adjusting for age at study, sex, ethnicity, educational attainment, insurance coverage, health status, and concern for future health.^39^  In comparison to survivors who did not received radiation therapy, those who did to the brain (OR 0.5; CI 0.4, 0.6), chest (OR 0.3; CI 0.2, 0.4), other (OR 0.5; CI 0.4, 0.6), or unknown (OR 0.5; CI 0.4, 0.8) sites were less likely to report general care versus risk-based survivorship care, after adjusting for sex, current age, and age at interview. In comparison to survivors who did not receive treatment with anthracyclines, those who received chest radiation (OR 0.4; CI 0.3, 0.6) and those who received anthracyclines and chest radiation (OR 0.5; CI 0.4, 0.6) were less likely to report general care versus risk-based survivorship care, after adjusting for sex, current age, and age at interview. In comparison to survivors who did not receive treatment with alkylating agents, those who received the highest dose in the third tertile (OR 0.6; CI 0.4, 0.9) were less likely to report general care versus risk-based survivorship care, after adjusting for sex, current age, and age at interview.^39,42^  **Age at diagnosis year of diagnosis, or time since diagnosis:** For each one-year increase in age at diagnosis, survivors experienced a decreased odds (OR 0.97; CI 0.95, 0.97) of having general care (rather than risk-based survivorship care), after adjusting for sex, race/ethnicity, current age, age at interview, annual household income, educational attainment, employment status, insurance coverage, poor emotional health, cancer-related anxiety, cancer-related pain, poor physical health, and grade of chronic disease.^39,42^  **Age at study or enrollment, current age:** In comparison to survivors 18-19 years of age at study, those who were ages 25-29 (OR 1.67; CI 1.38, 2.02), 30-34 (OR 1.74; CI 1.42, 2.14), and 35 years of age or more (OR 2.29; CI 1.83, 2.87) were more likely to report absence of a cancer related medical visit, after adjusting for sex, ethnicity, age at interview, educational attainment, health insurance, health status, concern for future health, and high-risk treatment. In comparison to survivors 18-19 years of age at study, those who were ages 20-24 (OR 1.31; CI 1.07, 1.61), 25-29 (OR 1.88; CI 1.51, 2.34), 30-34 (OR 2.79; CI 2.19, 3.56), and 35 years of age or more (OR 3.43; CI 2.61, 4.51) were more likely to report absence of a cancer center medical visit, after adjusting for sex, ethnicity, educational attainment, health insurance, health status, concern for future health, and high-risk treatment.^39^  For each one-year increase in age at study, survivors experienced an increased odds (OR 1.03; CI 1.02, 1.04) of having general care (rather than risk-based survivorship care), after adjusting for sex, race/ethnicity, annual household income, educational attainment, employment status, insurance coverage, poor emotional health, cancer-related anxiety, cancer-related pain, poor physical health, and grade of chronic disease.^39,42^  In comparison to survivors' who were <35 years of age, survivors who were 40-44 (OR 5.44; CI 1.07, 27.61), 45-49 (OR 12.97; CI 2.34, 71.84), 50-54 (OR 18.41; CI 2.87, 118.23), and 55+ (OR 28.33; CI 3.7, 217.15) experienced an increased likelihood of reporting physician-based skin exam, after adjusting for sex, education, age (years), race/ethnicity, skin type, age at diagnosis, diagnosis, chemotherapy (yes/no), highest CTCAE grade chronic condition, maximum radiotherapy dose, patient activation.^39,46^  In multivariable analysis, older age at interview was associated with an increased likelihood of reporting a mammogram (OR 1.08; CI 1.05, 1.11; after adjusting for race, marital status, educational attainment, insurance status, having medical care within the past two years, and having a cancer-related visit within the past two years) and colonoscopy (OR 1.07; CI 1.02, 1.12) after adjusting for sex, race, concern about future health, chronic disease status, poor emotional health, poor physical function, having a cancer treatment summary, having medical care within the past two years, and having a cancer-related visit within the past two years).^39,52^  **Cancer diagnosis:** In comparison to survivors' who were diagnosed with bone cancer, survivors who were diagnosed with central nervous system (OR 7.25; CI 1.25, 42.23), Wilms tumor (OR 6.67; CI 1.24, 36.02), or neuroblastoma (OR 8.30; CI 1.33, 51.96) experienced an increased likelihood of reporting physician-based skin exam, after adjusting for sex, education, age (years), race/ethnicity, skin type, age at diagnosis, diagnosis, chemotherapy (yes/no), highest CTCAE grade chronic condition, maximum radiotherapy dose, patient activation.^39,46^  **Other:** In comparison to survivors who reported good self-rated health, those who reported fair/poor self-rated health were less likely to report absence of a cancer related medical visit (OR 0.62; CI 0.52, 0.74), after adjusting for age at study, sex, ethnicity, educational attainment, insurance coverage, concern for future health, and high-risk treatment.^39^  In comparison to survivors who reported being concerned about their future health, those who reported not being concerned about their future health were more likely to report absence of cancer-related medical visits (OR 1.51; CI 1.33, 1.71) or cancer center medical visits (OR 1.53; CI 1.31, 1.79), after adjusting for age at study, sex, ethnicity, educational attainment, insurance coverage, health status, and high-risk treatment.^39^  In multivariable analysis, being married or living as married (OR 1.15; CI 1.06, 1.24) was associated with an increased likelihood of Papanicolaou smear adherence, after adjusting for race, age at interview, marital status, education, insurance status, and emotional health.^39,52^  Not significant: Survivors' self-rated health was not associated with absence of a cancer center medical visit, after adjusting for age at study, sex, ethnicity, educational attainment, insurance coverage, concern for future health, and high-risk treatment.^39^ | **Survivorship care:** Utilization of survivorship care services, care plans, or models of care (general medical contact, general medical physical exam, cancer-related medical visit, cancer center medical visit; general care versus risk-based survivor-focused care; survivor-focused versus general care versus preventive care; survivorship care in prior two years, planning cancer related visit within next two years; mammography screening for female CCS at risk for breast cancer; screening mammography; general health care versus general survivorship care versus risk-based survivorship care versus dental care; cancer screening in survivors at average risk of developing cervical or breast cancer and cancer surveillance in survivors at high-risk for breast, colorectal or skin cancer; accurate knowledge of diagnosis and treatment)  **Biomedical:**  Intermediate health outcomes and adverse events  Late effects and morbidity  **Psychosocial:** Psychological  Education attainment/ employment  **Health services/ economics:**  Primary care, specialty care, or other care utilization (dental services; screening mammography; planned utilization; worry about the cost leading to skipped medical test/ treatment/follow-up, no primary care provider, postponed preventive care; non-obstetric hospitalization)  Financial hardship, costs, and resource utilization  Other: dermatology care in prior two years; job lock |
| **Reppucci, 2017^54^**  *SuRFF Program at the Cohen Children’s Medical Center of New York*  Study size: 286^54^  Analysis type: Analytic study | USA | Proportion of CCS: 100%  Multiple cancer origin | **Treatment:** Those who received radiation therapy had greater odds of DXA scan adherence (OR 2.60; CI 1.39, 4.88) as compared with subjects who did not receive RT, after adjusting for sex, anthracycline exposure, transplant, age at diagnosis, and age at procedure recommendation.^54^  Not significant: Treatment with anthracycline or transplant were not significantly associated with recommendation adherence, after adjusting for sex, anthracycline exposure, transplant, age at diagnosis, and age at procedure recommendation.^54^  **Age at diagnosis year of diagnosis, or time since diagnosis:** As age at diagnosis increased, adherence with echocardiogram recommendations decreased (OR for a 10-year increase: 0.59; CI 0.37, 0.95), after adjusting for sex, radiation exposure, anthracycline exposure, transplant, and age at procedure recommendation.^54^  Not significant: Age at diagnosis was not significantly associated with adherence, after adjusting for sex, radiation exposure, anthracycline exposure, transplant, and age at procedure recommendation.^54^  **Age at study or enrollment, current age:** Those of older current age had reduced odds of adherence as compared with younger subjects (OR for a 10-year increase: 0.66; CI 0.50, 0.89) and those <18 years of age had greater odds of adherence as compared with subjects 18 years of age or older (OR 1.53; CI 1.04, 2.25). Survivors younger than 18 years of age had greater odds of echocardiogram adherence as compared with those 18 years and over (OR: 2.06, 95% CI: 1.02, 4.16). Both used multivariable models adjusting for sex, treatment with radiation, treatment with an anthracycline, treatment with a transplant, and age at diagnosis.^54^  **Cancer diagnosis:** Not significant: Cancer diagnosis was not significantly associated with adherence, after adjusting for sex, radiation exposure, anthracycline exposure, transplant, age at procedure recommendation, age at diagnosis.^54^ | **Survivorship care:** Utilization of survivorship care services, care plans, or models of care (adherence to survivorship care plan and surveillance) |
| **Streefkerk, 2019^55^**  *DCOG-LATER*  Study size: 602 survivors, 1204 controls^55^  Analysis type: Analytic study | Netherlands | Proportion of CCS: 100%  Multiple cancer origin | **Sex:** Females had more contacts with their PCP (contact ratio ratio 1.96; CI 1.72, 2.23) in comparison to males, after adjusting for sex, attained age, and treatment received.^55^  **Treatment:** Those treated with surgery only (contact rate ratio 1.23; 1.00, 1.51) and with radiotherapy only (contact rate ratio 1.49; 1.15, 1.93) had more PCP contact during follow-up compared with those treated with chemotherapy only, when adjusting for sex and attained age.^55^  **Age at study or enrollment, current age:** In comparison to those <20 years, survivors aged 20 years or older had a significantly higher number of contacts with their PCP (20-29 years contact rate ratio 1.23; CI 1.00, 1.53; 30-39 years 1.53; 1.23, 1.90; >/= 40 years 1.58; CI 1.23, 2.04), after adjusting for sex, attained age, and treatment received.^55^  **Cancer diagnosis:** Not significant: Cancer diagnosis was not significantly associated with the number of contacts with the primary care physician, when adjusting for sex and current age.^55^ | **Health services/ economics:**  Primary care, specialty care, or other care utilization (number of contacts with PCP per year of follow-up) |
| **Szalda, 2016^56^**  Szalda, 2017^57^  *Cancer Survivorship Program at CHOP*  Study size: 80^56,57^  Analysis type: Survey, analytic study | USA | Proportion of CCS: 100%  Multiple cancer origin | **Insurance:** In univariate analysis, having insurance was the only significant demographic associate of engagement with adult-oriented follow-up care (p<005); none of the patients without insurance had follow-up care).^56,57^  **Age at diagnosis year of diagnosis, or time since diagnosis:** Survivors who were older ages at diagnosis (OR 0.90; CI 0.82, 0.99) were less likely to access survivorship care for each year of age increase, after adjusting for knowledge of risk for second cancer, comfort discussing concerns, and motivation to take care of health.^56,57^ | **Survivorship care:** Utilization of survivorship care services, care plans, or models of care (no survivorship care versus subspecialty follow-up care versus PCP follow-up)  **Health services/ economics:**  Primary care, specialty care, or other care utilization  Quality of life/ satisfaction with care |
| **vanLaar, 2013^58^**  Study size: 143^58^  Analysis type: Survey | UK | Proportion of CCS: 100%  Multiple cancer origin | **Age at study or enrollment, current age:** Older participants were less likely to attend all of their clinic appointments (p<0.05).^58^ | **Survivorship care:** Utilization of survivorship care services, care plans, or models of care (attendence and satisfaction)  **Health services/ economics:**  Quality of life/ satisfaction with care |
| **Welch, 2017^59^**  *Consortium for New England Childhood Cancer Survivors*  Study size: 317^59^  Analysis type: Analytic study | USA | Proportion of CCS: 100%  Leukemia | **Race/ethnicity:** Not significant: Race was not a significant predictor of follow-up at 5 and 10 years (adjusting for clustering of institutions).^59^  **Sex:** Not significant: Sex was not a significant predictor of follow-up at 5 and 10 years (adjusting for clustering of institutions).^59^  **Insurance:** At 5 years from diagnosis, patients were more likely to participate in follow-up if they had insurance at diagnosis (OR 3.4, 95% CI 1.2, 9.9), controlling for institutional clustering. At 10 years after diagnosis, insurance status at diagnosis remained a strong predictor (OR 3.7, 95% CI 1.0, 9.1) for follow-up care, controlling for institutional clustering. At 5- and 10- years post-diagnosis, public insurance coverage was negatively associated with follow-up care (5-year follow-up: OR 0.58, 95% CI 0.17, 1.0; 10-year follow-up: OR 0.48, 95% CI 0.22, 0.73), when compared to those with private or military insurance coverage. At 5- and 10- years post-diagnosis, being uninsured was negatively associated with follow-up care (5-year follow-up: OR 0.24, 95% CI 0.026, 0.46; 10-year follow-up: OR 0.28, 95% CI 0.11, 0.71), when compared to those with private or military insurance coverage.^59^  **Treatment:** Not significant: Clinical trial participation (enrolled on a treatment study) was not significantly associated with 5- or 10- year follow-up (adjusting for clustering of institutions).^59^  **Age at study or enrollment, current age:** At 5 years from diagnosis, patients were less likely to participate in follow-up if they were over age 18 at the time of follow-up (OR 0.37, 95% CI 0.15, 0.91; controlling for clustering of institutions).^59^  Not significant: Current age at the time of follow-up was not significantly associated with 10-year follow-up (adjusting for clustering of institutions).^59^  **Cancer diagnosis:** At 10 years post-diagnosis, leukemia with CNS involvement was negatively associated (OR 0.26, 95% CI 0.081, 0.82) with follow-up care, after controlling for clustering of institutions.^59^  Not significant: CNS involvement of leukemia was not significantly associated with 5-year follow-up. High-risk leukemia and relapsed leukemia were not significantly associated with 5- or 10- year follow-up (after controlling for clustering of institutions).^59^ | **Survivorship care:** Utilization of survivorship care services, care plans, or models of care (survivorship visits at treating institution at 5 and 10 years post-diagnosis)  **Health services/ economics:**  Primary care, specialty care, or other care utilization |
| **Zanetti, 2022^60^**  *DOD MHS Data Repository*  Study size: 144  Analysis type: Analytic study | USA | Proportion of CCS: 100%  Leukemia | **Sex:** Not significant: In multivariable analysis, sex was not a significant predictor of echocardiogram screening or mental health visit, after adjusting for age, sponsor rank group, and type of care (military versus civilian).  **Age at diagnosis year of diagnosis, or time since diagnosis:** In multivariable analysis, being age 10-19 years at diagnosis was associated with decreased odds of receiving a dual-energy X-ray absorptiometry (DEXA) scan (OR 0.32; CI 0.11, 0.95) and mental health visits (OR 0.28; CI 0.11-0.70), respectively, after adjusting for sex, sponsor rank group, and type of care (military versus civilian).  Not significant: In multivariable analysis, age group was not a significant predictor of echocardiogram screening, after adjusting for sex, sponsor rank group, and type of care (military versus civilian).  **Other:** In multivariable analysis, being diagnosed with anxiety was associated with increased odds of receiving a dual-energy X-ray absorptiometry (DEXA) scan (OR 4.62; CI 1.68-12.66), after adjusting for age, sex, sponsor rank group, and type of care (military versus civilian). In multivariable analysis, having a composite measure of one or more mental health diagnoses was associated with increased odds of receiving a mental health visits (OR 2.67; 1.19, 5.99), after adjusting for age, sex, sponsor rank group, and type of care (military versus civilian).  Not significant: In multivariable analysis, sponsor rank group, type of care (military versus civilian), and cardiovascular late effects were not significant predictors of receiving a dual-energy X-ray absorptiometry (DEXA) scan, after adjusting for age and sex. | **Survivorship care:** Utilization of survivorship care services, care plans, or models of care (dual-energy X-ray absorptiometry, DEXA, scan, echocardiogram  **Health services/ economics:**  Primary care, specialty care, or other care utilization (mental health visit) |
| **Zheng, 2016^61^**  Study size: 489^61^  Analysis type: Analytic study | USA | Proportion of CCS: 100%  Multiple cancer origin | **Race/ethnicity:** Not significant: Survivors who were non-Hispanic black, Hispanic, or other races/ethnicities (in comparison to those who were non-Hispanic white) did not experience a significant association with survivorship clinic attendence, after adjusting for sex, age at diagnosis, and insurance.^61^  **Income:** Not significant: Survivors with incomes greater than $68,999 (in comparison to those with incomes less than $69,000) did not experience a significant association with survivorship clinic attendence, after adjusting for sex, age at diagnosis, and insurance.^61^  **Underserved or rural:** Not significant: Survivors who traveled more than 15 minutes to the hospital (in comparison to those with travel times less than 15 minutes) did not experience a significant association with survivorship clinic attendence, after adjusting for sex, age at diagnosis, and insurance.^61^  **Sex:** Not significant: There was not a significant association with survivorship clinic attendence by sex, after adjusting for age at diagnosis and insurance.^61^  **Insurance:** After adjusting for sex and age at diagnosis, survivors covered by private insurance (HR 2.90; 1.75, 4.81) or public insurance (HR 2.05; CI 1.14, 3.71) were more likely to attend survivorship clinic in comparison to those without insurance.^61^  **Treatment:** Survivors who received treatment with surgery only (HR 0.02; CI 0.00, 0.13) or surgery and chemotherapy (HR 0.54; 0.30, 0.97) were less likely to attend survivorship clinic in comparison to those who received treatment with surgery, chemotherapy, and radiation. Those who received radiation exposure to the head/neck (HR 1.88; CI 1.28, 2.77), chest (HR 2.72; CI 1.71, 4.34), or abdomen/pelvis (HR 1.78; 1.01, 3.13) were more likely to attend survivorship clinic in comparison to those who received no radiation. Survivors who received alkylating agents (HR 2.28; CI 1.58, 3.30), anthracyclines (HR 3.05; CI 2.09, 4.44) , or lung toxic therapies (HR 1.89; 1.19. 3.00) were more likely to attend survivorship clinic in comparison to those who received no chemotherapy. All models adjusted for sex, age at diagnosis, and insurance status.^61^  Not significant: Those treated with chemotherapy only or radiation +/- chemotherapy did not have a significant association with attendence at survivorship clinic, in comparison to those treated with surgery, chemotherapy, and radiation. In comparison to those who received no radiation, survivors who received full body radiation did not have a significant association with survivorship clinic attendence. Survivors who enrolled on a clinical trial (in comparison to did not enroll) or who did not experience a relapse (in comparison to those who did relapse) did not experience a significant association with survivorship clinic attendence. All models were adjusted for sex, age at diagnosis, and insurance status.^61^  **Age at diagnosis year of diagnosis, or time since diagnosis:** Not significant:  There was not a significant association with survivorship clinic attendence by age at diagnosis, after adjusting for sex and insurance.^61^  **Cancer diagnosis:** After adjusting for sex, age at diagnosis, and insurance status, survivors diagnosed with leukemia (HR 3.36, 1.65, 6.83), lymphoma (HR 3.99, 1.87, 8.53), and sarcoma (HR 3.30 , 1.47, 7.42) were more likely to attend survivorship clinic in comparison to those diagnosed with central nervous system tumors.^61^  Not significant: Those diagnosed with thyroid cancer/melanoma or other solid tumors did not have a significant association with attendence at survivorship clinic, after adjusting for sex, age at diagnosis, and insurance, in comparison to those diagnosed with central nervous system tumors.^61^ | **Survivorship care:** Utilization of survivorship care services, care plans, or models of care (survivorship clinic attendence) |

**Table A-3. Risk of bias assessment**

| **Author, year** | **Study participation** | **Study attrition** | **Prognostic factor measurement** | **Outcome measurement** | **Study confounding** | **Statistical analysis and reporting** | **Other sources of bias** | **Overall risk of bias** |
| --- | --- | --- | --- | --- | --- | --- | --- | --- |
| Baedke, 2022^1^ | Moderate/unclear | Low | Low | Moderate/unclear | Low | Low | Moderate/unclear | Neutal/moderate |
| Barakat, 2012^4^ | Moderate/unclear | Moderate/unclear | Moderate/unclear | Moderate/unclear | Moderate/unclear | Low | Moderate/unclear | Neutal/moderate |
| Benedict, 2021^5^ | Moderate/unclear | Moderate/unclear | Low | Low | Moderate/unclear | Low | Moderate/unclear | Low |
| Berg, 2016^6^ | Moderate/unclear | Moderate/unclear | Low | Low | Moderate/unclear | Low | Moderate/unclear | Low |
| Berkman, 2019^7^ | Moderate/unclear | Moderate/unclear | Low | Low | Low | Low | Moderate/unclear | Low |
| Crom, 2007^8^ | Moderate/unclear | Moderate/unclear | Low | Moderate/unclear | Low | Low | Moderate/unclear | Neutal/moderate |
| Daly, 2019^11^ | Moderate/unclear | Low | Low | Low | Low | Low | Moderate/unclear | Low |
| Gardner, 2014^12^ | Low | Moderate/unclear | Moderate/unclear | Low | Moderate/unclear | Low | Moderate/unclear | Low |
| Johnson, 2004^13^ | Low | Low | Low | Moderate/unclear | Moderate/unclear | Low | Moderate/unclear | Neutal/moderate |
| May, 2017^14^ | Moderate/unclear | Low | Low | Moderate/unclear | Moderate/unclear | Low | Moderate/unclear | Neutal/moderate |
| McBride, 2011^15^ | Moderate/unclear | Moderate/unclear | Moderate/unclear | Moderate/unclear | Moderate/unclear | Low | Moderate/unclear | Neutal/moderate |
| Michel, 2011^16^ | Low | Low | Moderate/unclear | Low | Low | Low | Moderate/unclear | Low |
| Milam, 2015^23^ | Moderate/unclear | Low | Low | Moderate/unclear | Low | Low | Moderate/unclear | Neutal/moderate |
| Nathan, 2016^36^ | Moderate/unclear | Moderate/unclear | Moderate/unclear | Moderate/unclear | Moderate/unclear | Low | Moderate/unclear | Neutal/moderate |
| Oeffinger, 2004^39^ | Moderate/unclear | Moderate/unclear | Low | Moderate/unclear | Moderate/unclear | Low | Moderate/unclear | Neutal/moderate |
| Reppucci, 2017^54^ | Moderate/unclear | Moderate/unclear | Low | Low | Moderate/unclear | Low | Moderate/unclear | Low |
| Streefkerk, 2019^55^ | Moderate/unclear | Moderate/unclear | Moderate/unclear | Moderate/unclear | Low | Low | Moderate/unclear | Neutal/moderate |
| Szalda, 2016^56^ | Moderate/unclear | Low | Low | Moderate/unclear | Low | Low | Moderate/unclear | Neutal/moderate |
| vanLaar, 2013^58^ | Moderate/unclear | Moderate/unclear | Low | Low | Moderate/unclear | Low | Moderate/unclear | Low |
| Welch, 2017^59^ | Low | Moderate/unclear | Low | Moderate/unclear | Moderate/unclear | Low | Moderate/unclear | Neutal/moderate |
| Zanetti, 2022^60^ | Low | Low | Moderate/unclear | Moderate/unclear | Low | Low | Moderate/unclear | Neutal/moderate |
| Zheng, 2016^61^ | Low | Low | Moderate/unclear | Moderate/unclear | Low | Low | Moderate/unclear | Neutal/moderate |

Notes:

Study participation: The study should report a. Adequate participation in the study by eligible persons, b. Description of the source population or population of interest, c. Description of the baseline study sample, d. Adequate description of the sampling frame, and recruitment, e. Adequate description of the period and place of recruitment, f. Adequate description of inclusion and exclusion criteria. High risk: The relationship between the predictor variable and outcome is very likely to be different for participants and eligible nonparticipants; Moderate/unclear bias: The relationship between the predictor variable and outcome may be different for participants and eligible nonparticipants; Low bias: The relationship between the predictor variable and outcome is unlikely to be different for participants and eligible nonparticipants.

Study attrition: a. Adequate response rate for study participants, b. Description of attempts to collect information on participants who dropped out, c. Reasons for loss to follow-up are provided, d. Adequate description of participants lost to follow-up, e. There are no important differences between participants who completed the study and those who did not. High bias: The relationship between the predictor variable and outcome is very likely to be different for completing and non-completing participants; Moderate/unclear bias: The relationship between the predictor variable and outcome may be different for completing and non-completing participants; Low bias: The relationship between the predictor variable and outcome is unlikely to be different for completing and non-completing participants.

Prognostic factor measurement: The study should report: a. A clear definition or description of the predictor variable is provided, b. Method of predictor variable measurement is adequately valid and reliable, c. Continuous variables are reported or appropriate cut points are used, d. The method and setting of measurement of predictor variable is the same for all study participants, e. Adequate proportion of the study sample has complete data for the predictor variable, f. Appropriate methods of imputation are used for missing predictor variable data. High bias: The measurement of the predictor variable is very likely to be different for different levels of the outcome of interest; Moderate/unclear bias: The measurement of the predictor variable may be different for different levels of the outcome of interest, this is the default; Low bias: The measurement of the predictor variable is unlikely to be different for different levels of the outcome of interest, state why in the free text field; this point was given if the study used the entire sample to predict survivorship care with predictors of interest such as race, sex, gender, education; i.e., social, economic, and/or environmental disadvantages (not subgroups within subgroups, e.g., there was an effect for African-American but not for Hispanic participants [the question the review wants to answer is ‘was there an effect of race’]).

Outcome measurement: The study should report: a. clear definition of the outcome is provided, b. Method of outcome measurement used is adequately valid and reliable, c. The method and setting of outcome measurement is the same for all study participants. The review evaluated receiving care, in particular survivorship care. High bias: The measurement of the outcome is very likely to be different related to the baseline level of the predictor variable; Moderate/unclear bias: The measurement of the outcome may be different related to the baseline level of the predictor variable, this is the default; Low bias: The measurement of the outcome is unlikely to be different related to the baseline level of the predictor variable; studies predicting survivorship care specifically were classified as low risk of bias.

Study confounding: The study should report: a. All important confounders are measured, b. Clear definitions of the important confounders measured are provided, c. Measurement of all important confounders is adequately valid and reliable, d. The method and setting of confounding measurement are the same for all study participants, e. Appropriate methods are used if imputation is used for missing confounder data, f. Important potential confounders are accounted for in the study design, g. Important potential confounders are accounted for in the analysis. High bias: The observed effect of the predictor variable on the outcome is very likely to be distorted by another factor related to predictor variable and outcome; Moderate/unclear bias: The observed effect of the predictor variable on outcome may be distorted by another factor related to predictor variable and outcome; Low bias: The observed effect of the predictor variable on outcome is unlikely to be distorted by another factor related to predictor variable and outcome; this point was given if the study discussed confounders or explicitly mentioned possible confounders and how these were adjusted for.

Statistical analysis and reporting: The study should report: a. Sufficient presentation of data to assess the adequacy of the analytic strategy, b. Strategy for model building is appropriate and is based on a conceptual framework or model, c. The selected statistical model is adequate for the design of the study, d. There is no selective reporting of results. High bias: The reported results are very likely to be spurious or biased related to analysis or reporting, state why in the free text field. Studies only reporting on univariate analyses rather than multivariate analyses of competing socioeconomic predictors, and studies not reporting statistical tests (p-values or confidence intervals), were graded as high risk. Moderate bias: The reported results may be spurious or biased related to analysis or reporting; Low bias: The reported results are unlikely to be spurious or biased related to analysis or reporting.

**REFERENCES**

1. Baedke JL, Lindsey LA, James AS, et al. Forgoing needed medical care among long-term survivors of childhood cancer: racial/ethnic-insurance disparities. *J Cancer Surviv*. Jun 2022;16(3):677-687. doi:10.1007/s11764-021-01061-3

2. Hudson MM, Ness KK, Gurney JG, et al. Clinical ascertainment of health outcomes among adults treated for childhood cancer. *JAMA*. Jun 12 2013;309(22):2371-2381. doi:10.1001/jama.2013.6296

3. Howell CR, Bjornard KL, Ness KK, et al. Cohort Profile: The St. Jude Lifetime Cohort Study (SJLIFE) for paediatric cancer survivors. *Int J Epidemiol*. Mar 3 2021;50(1):39-49. doi:10.1093/ije/dyaa203

4. Barakat LP, Schwartz LA, Szabo MM, Hussey HM, Bunin GR. Factors that contribute to post-treatment follow-up care for survivors of childhood cancer. *J Cancer Surviv*. Jun 2012;6(2):155-62. doi:10.1007/s11764-011-0206-6

5. Benedict C, Wang J, Reppucci M, Schleien CL, Fish JD. Cost of survivorship care and adherence to screening-aligning the priorities of health care systems and survivors. *Transl Behav Med*. Feb 11 2021;11(1):132-142. doi:10.1093/tbm/ibz182

6. Berg CJ, Stratton E, Esiashvili N, Mertens A. Young Adult Cancer Survivors' Experience with Cancer Treatment and Follow-Up Care and Perceptions of Barriers to Engaging in Recommended Care. *J Cancer Educ*. Sep 2016;31(3):430-42. doi:10.1007/s13187-015-0853-9

7. Berkman JM, Dallas J, Lim J, et al. Social determinants of health affecting treatment of pediatric brain tumors. *J Neurosurg Pediatr*. May 24 2019:1-7. doi:10.3171/2019.4.Peds18594

8. Crom DB, Lensing SY, Rai SN, Snider MA, Cash DK, Hudson MM. Marriage, employment, and health insurance in adult survivors of childhood cancer. *J Cancer Surviv*. Sep 2007;1(3):237-45. doi:10.1007/s11764-007-0026-x

9. Klosky JL, Cash DK, Buscemi J, et al. Factors influencing long-term follow-up clinic attendance among survivors of childhood cancer. *J Cancer Surviv*. Dec 2008;2(4):225-32. doi:10.1007/s11764-008-0063-0

10. Hudson MM, Tyc VL, Srivastava DK, et al. Multi-component behavioral intervention to promote health protective behaviors in childhood cancer survivors: the protect study. *Med Pediatr Oncol*. Jul 2002;39(1):2-1; discussion 2. doi:10.1002/mpo.10071

11. Daly A, Lewis RW, Vangile K, et al. Survivor clinic attendance among pediatric- and adolescent-aged survivors of childhood cancer. *J Cancer Surviv*. Feb 2019;13(1):56-65. doi:10.1007/s11764-018-0727-3

12. Gardner MH, Barnes MJ, Bopanna S, et al. Barriers to the Use of Psychosocial Support Services Among Adolescent and Young Adult Survivors of Pediatric Cancer. *Journal of Adolescent & Young Adult Oncology*. 2014;3(3):112-116. doi:10.1089/jayao.2013.0036

13. Johnson R, Horne B, Feltbower RG, Butler GE, Glaser AW. Hospital attendance patterns in long term survivors of cancer. *Archives of disease in childhood*. Apr 2004;89(4):374-7. doi:10.1136/adc.2002.021816

14. May L, Schwartz DD, Frugé E, et al. Predictors of Suboptimal Follow-up in Pediatric Cancer Survivors. *J Pediatr Hematol Oncol*. Apr 2017;39(3):e143-e149. doi:10.1097/mph.0000000000000723

15. McBride ML, Lorenzi MF, Page J, et al. Patterns of physician follow-up among young cancer survivors: report of the Childhood, Adolescent, and Young Adult Cancer Survivors (CAYACS) research program. *Can Fam Physician*. Dec 2011;57(12):e482-90.

16. Michel G, Kuehni CE, Rebholz CE, et al. Can health beliefs help in explaining attendance to follow-up care? The Swiss childhood cancer survivor study. *Psychooncology*. Oct 2011;20(10):1034-43. doi:10.1002/pon.1823

17. Michel G, Gianinazzi ME, Eiser C, et al. Preferences for long-term follow-up care in childhood cancer survivors. *Eur J Cancer Care (Engl)*. Nov 2016;25(6):1024-1033. doi:10.1111/ecc.12560

18. Lupatsch JE, Wengenroth L, Rueegg CS, et al. Follow-up care of adolescent survivors of childhood cancer: The role of health beliefs. *Pediatr Blood Cancer*. Feb 2016;63(2):318-25. doi:10.1002/pbc.25755

19. Gianinazzi ME, Rueegg CS, von der Weid NX, Niggli FK, Kuehni CE, Michel G. Mental health-care utilization in survivors of childhood cancer and siblings: the Swiss childhood cancer survivor study. *Support Care Cancer*. Feb 2014;22(2):339-49. doi:10.1007/s00520-013-1976-3

20. Vetsch J, Rueegg CS, Mader L, et al. Follow-up care of young childhood cancer survivors: attendance and parental involvement. *Support Care Cancer*. Jul 2016;24(7):3127-38. doi:10.1007/s00520-016-3121-6

21. Michel G, Gianinazzi ME, Vetsch J, et al. Physicians' experience with follow-up care of childhood cancer survivors - challenges and needs. *Swiss Med Wkly*. 2017;147:w14457. doi:10.4414/smw.2017.14457

22. Hendriks MJ, Harju E, Roser K, Ienca M, Michel G. The long shadow of childhood cancer: a qualitative study on insurance hardship among survivors of childhood cancer. *BMC Health Serv Res*. May 25 2021;21(1):503. doi:10.1186/s12913-021-06543-9

23. Milam JE, Meeske K, Slaughter RI, et al. Cancer-related follow-up care among Hispanic and non-Hispanic childhood cancer survivors: The Project Forward study. *Cancer*. 2015;121(4):605-613. doi:10.1002/cncr.29105

24. Miller KA, Ramirez CN, Wojcik KY, et al. Prevalence and correlates of health information-seeking among Hispanic and non-Hispanic childhood cancer survivors. *Support Care Cancer*. Apr 2018;26(4):1305-1313. doi:10.1007/s00520-017-3956-5

25. Miller KA, Wojcik KY, Ramirez CN, et al. Supporting long-term follow-up of young adult survivors of childhood cancer: Correlates of healthcare self-efficacy. *Pediatr Blood Cancer*. Feb 2017;64(2):358-363. doi:10.1002/pbc.26209

26. Cousineau MR, Kim SE, Hamilton AS, Miller KA, Milam J. Insurance Coverage, and Having a Regular Provider, and Utilization of Cancer Follow-up and Noncancer Health Care Among Childhood Cancer Survivors. *Inquiry*. Jan-Dec 2019;56:46958018817996. doi:10.1177/0046958018817996

27. Slaughter RI. *Childhood cancer survivorship: Parental factors associated with survivor's follow-up care behavior and mental health*. University of Southern California; May 2018.

28. Tobin JL. *Multilevel Sociodemographic Correlates of the Health and Healthcare Utilization of Childhood Cancer Survivors*. University of Southern California; 2020.

29. Sleight AG, Ramirez CN, Miller KA, Milam JE. Hispanic Orientation and Cancer-Related Knowledge in Childhood Cancer Survivors. *J Adolesc Young Adult Oncol*. Jun 2019;8(3):363-367. doi:10.1089/jayao.2018.0099

30. Slaughter RI, Hamilton AS, Cederbaum JA, Unger JB, Baezconde-Garbanati L, Milam JE. Relationships between parent and adolescent/young adult mental health among Hispanic and non-Hispanic childhood cancer survivors. *Journal of Psychosocial Oncology*. 2020:1-15. doi:10.1080/07347332.2020.1815924

31. Ochoa CY, Cho J, Miller KA, et al. The Impact of Hispanic Ethnicity and Language on Communication Among Young Adult Childhood Cancer Survivors, Parents, and Medical Providers and Cancer-Related Follow-Up Care. *JCO Oncol Pract*. May 2022;18(5):e786-e796. doi:10.1200/op.22.00005

32. Ochoa CY, Miller KA, Baezconde-Garbanati L, Slaughter RI, Hamilton AS, Milam JE. Parental Cancer-related Information Seeking, Health Communication and Satisfaction with Medical Providers of Childhood Cancer Survivors: Differences by Race/Ethnicity and Language Preference. *J Health Commun*. Feb 1 2021;26(2):83-91. doi:10.1080/10810730.2021.1895919

33. Wojcik KY, Miller KA, Wysong A, et al. Barriers to Physician-Based Skin Examinations for Adolescent and Young Adult Survivors of Melanoma in the Project Forward Study. *JAMA Dermatol*. Jul 1 2021;157(7):874-876. doi:10.1001/jamadermatol.2021.1850

34. Milam J, Freyer DR, Miller KA, et al. Project Forward: A Population-Based Cohort Among Young Adult Survivors of Childhood Cancers. *JNCI Cancer Spectr*. Oct 2021;5(5)doi:10.1093/jncics/pkab068

35. Mobley EM, Kim SE, Cousineau M, et al. Insurance coverage change and survivorship care among young adult survivors of childhood cancer. *Health Serv Res*. Feb 2022;57(1):159-171. doi:10.1111/1475-6773.13868

36. Nathan PC, Agha M, Pole JD, et al. Predictors of attendance at specialized survivor clinics in a population-based cohort of adult survivors of childhood cancer. *J Cancer Surviv*. Aug 2016;10(4):611-8. doi:10.1007/s11764-016-0522-y

37. Marr KC, Agha M, Sutradhar R, et al. Specialized survivor clinic attendance increases adherence to cardiomyopathy screening guidelines in adult survivors of childhood cancer. *J Cancer Surviv*. Oct 2017;11(5):614-623. doi:10.1007/s11764-017-0634-z

38. Sutradhar R, Agha M, Pole JD, et al. Specialized survivor clinic attendance is associated with decreased rates of emergency department visits in adult survivors of childhood cancer. *Cancer*. Dec 15 2015;121(24):4389-97. doi:10.1002/cncr.29679

39. Oeffinger KC, Mertens AC, Hudson MM, et al. Health Care of Young Adult Survivors of Childhood Cancer: A Report from the Childhood Cancer Survivor Study. *Annals of Family Medicine*. 2004;2(1):61-70.

40. Kirchhoff AC, Kuhlthau K, Pajolek H, et al. Employer-sponsored health insurance coverage limitations: results from the Childhood Cancer Survivor Study. journal article. *Supportive Care in Cancer*. February 01 2013;21(2):377-383. doi:10.1007/s00520-012-1523-7

41. Casillas JN, Oeffinger KC, Hudson MM, et al. Identifying Predictors of Longitudinal Decline in the Level of Medical Care Received by Adult Survivors of Childhood Cancer: A Report from the Childhood Cancer Survivor Study. *Health Services Research*. 2015;50(4):1021-1042.

42. Nathan PC, Greenberg ML, Ness KK, et al. Medical care in long-term survivors of childhood cancer: a report from the Childhood Cancer Survivor Study. *Journal of Clinical Oncology*. 2008;26(27):4401-4409.

43. Caplin DA, Smith KR, Ness KK, et al. Effect of Population Socioeconomic and Health System Factors on Medical Care of Childhood Cancer Survivors: A Report from the Childhood Cancer Survivor Study. *J Adolesc Young Adult Oncol*. Mar 2017;6(1):74-82. doi:10.1089/jayao.2016.0016

44. Casillas J, Castellino SM, Hudson MM, et al. Impact of insurance type on survivor-focused and general preventive health care utilization in adult survivors of childhood cancer: the Childhood Cancer Survivor Study (CCSS). *Cancer*. May 1 2011;117(9):1966-75. doi:10.1002/cncr.25688

45. Castellino SM, Casillas J, Hudson MM, et al. Minority adult survivors of childhood cancer: a comparison of long-term outcomes, health care utilization, and health-related behaviors from the childhood cancer survivor study. *J Clin Oncol*. Sep 20 2005;23(27):6499-507. doi:10.1200/jco.2005.11.098

46. Geller AC, Keske RR, Haneuse S, et al. Skin Cancer Early Detection Practices among Adult Survivors of Childhood Cancer Treated with Radiation. *J Invest Dermatol*. Sep 2019;139(9):1898-1905.e2. doi:10.1016/j.jid.2019.02.033

47. Kaste SC, Goodman P, Leisenring W, et al. Impact of radiation and chemotherapy on risk of dental abnormalities: a report from the Childhood Cancer Survivor Study. *Cancer*. Dec 15 2009;115(24):5817-27. doi:10.1002/cncr.24670

48. Kirchhoff AC, Nipp R, Warner EL, et al. "Job Lock" Among Long-term Survivors of Childhood Cancer: A Report From the Childhood Cancer Survivor Study. *JAMA Oncol*. May 1 2018;4(5):707-711. doi:10.1001/jamaoncol.2017.3372

49. Nipp RD, Kirchhoff AC, Fair D, et al. Financial Burden in Survivors of Childhood Cancer: A Report From the Childhood Cancer Survivor Study. *J Clin Oncol*. Oct 20 2017;35(30):3474-3481. doi:10.1200/jco.2016.71.7066

50. Recklitis CJ, Diller LR, Li X, Najita J, Robison LL, Zeltzer L. Suicide ideation in adult survivors of childhood cancer: a report from the Childhood Cancer Survivor Study. *J Clin Oncol*. Feb 1 2010;28(4):655-61. doi:10.1200/jco.2009.22.8635

51. Yeazel MW, Gurney JG, Oeffinger KC, et al. An examination of the dental utilization practices of adult survivors of childhood cancer: a report from the Childhood Cancer Survivor Study. *J Public Health Dent*. Winter 2004;64(1):50-4. doi:10.1111/j.1752-7325.2004.tb02726.x

52. Nathan PC, Ness KK, Mahoney MC, et al. Screening and surveillance for second malignant neoplasms in adult survivors of childhood cancer: a report from the childhood cancer survivor study. *Ann Intern Med*. Oct 5 2010;153(7):442-51. doi:10.7326/0003-4819-153-7-201010050-00007

53. Krull KR, Annett RD, Pan Z, et al. Neurocognitive functioning and health-related behaviours in adult survivors of childhood cancer: a report from the Childhood Cancer Survivor Study. *Eur J Cancer*. Jun 2011;47(9):1380-8. doi:10.1016/j.ejca.2011.03.001

54. Reppucci ML, Schleien CL, Fish JD. Looking for trouble: Adherence to late-effects surveillance among childhood cancer survivors. *Pediatr Blood Cancer*. Feb 2017;64(2):353-357. doi:10.1002/pbc.26205

55. Streefkerk N, Heins MJ, Teepen JC, et al. The involvement of primary care physicians in care for childhood cancer survivors. *Pediatric Blood & Cancer*. 2019;66(8):1-9. doi:10.1002/pbc.27774

56. Szalda D, Pierce L, Hobbie W, et al. Engagement and experience with cancer-related follow-up care among young adult survivors of childhood cancer after transfer to adult care. *J Cancer Surviv*. Apr 2016;10(2):342-50. doi:10.1007/s11764-015-0480-9

57. Szalda D, Piece L, Brumley L, et al. Associates of Engagement in Adult-Oriented Follow-Up Care for Childhood Cancer Survivors. *J Adolesc Health*. Feb 2017;60(2):147-153. doi:10.1016/j.jadohealth.2016.08.018

58. van Laar M, Glaser A, Phillips RS, Feltbower RG, Stark DP. The impact of a managed transition of care upon psychosocial characteristics and patient satisfaction in a cohort of adult survivors of childhood cancer. *Psychooncology*. Sep 2013;22(9):2039-45. doi:10.1002/pon.3248

59. Welch JJG, Kenney LB, Hirway P, et al. Understanding predictors of continued long-term pediatric cancer care across the region: A report from the Consortium for New England Childhood Cancer Survivors. *Pediatr Blood Cancer*. Oct 2017;64(10)doi:10.1002/pbc.26564

60. Zanetti R, Warwick A, Sicignano N, Feldman B. Late Effects Screening of Acute Lymphoblastic Leukemia Survivors in the Military Healthcare System. *Mil Med*. Mar 21 2022;doi:10.1093/milmed/usac069

61. Zheng DJ, Sint K, Mitchell HR, Kadan-Lottick NS. Patterns and predictors of survivorship clinic attendance in a population-based sample of pediatric and young adult childhood cancer survivors. *J Cancer Surviv*. Jun 2016;10(3):505-13. doi:10.1007/s11764-015-0493-4
